# A pre-metazoan origin of the *CRK* gene family and co-opted signaling network

**DOI:** 10.1038/srep34349

**Published:** 2016-09-30

**Authors:** Yoko Shigeno-Nakazawa, Takuma Kasai, Sewon Ki, Elina Kostyanovskaya, Jana Pawlak, Junya Yamagishi, Noriaki Okimoto, Makoto Taiji, Mariko Okada, Jody Westbrook, Yoko Satta, Takanori Kigawa, Akira Imamoto

**Affiliations:** 1 The Ben May Department for Cancer Research, The University of Chicago, USA; 2 RIKEN Quantitative Biology Center (QBiC), Yokohama, Kanagawa, Japan; 3 RIKEN Center for Integrative Medical Sciences (IMS), Yokohama, Kanagawa, Japan; 4 RIKEN Quantitative Biology Center (QBiC), Suita, Osaka, Japan; 5 Department of Molecular and Cell Biology, University of California Berkeley, Berkeley, CA, USA; 6 Department of Evolutionary Studies of Biosystems, The Graduate University of Advanced Studies, Miura-gunn, Kanagawa, Japan

## Abstract

CRK and CRKL adapter proteins play essential roles in development and cancer through their SRC homology 2 and 3 (SH2 and SH3) domains. To gain insight into the origin of their shared functions, we have investigated their evolutionary history. We propose a term, *crk/crkl ancestral (crka*), for orthologs in invertebrates before the divergence of *CRK* and *CRKL* in the vertebrate ancestor. We have isolated two orthologs expressed in the choanoflagellate *Monosiga brevicollis*, a unicellular relative to the metazoans. Consistent with its highly-conserved three-dimensional structure, the SH2 domain of *M. brevicollis* crka1 can bind to the mammalian CRK/CRKL SH2 binding consensus phospho-YxxP, and to the SRC substrate/focal adhesion protein BCAR1 (p130^CAS^) in the presence of activated SRC. These results demonstrate an ancient origin of the CRK/CRKL SH2-target recognition specificity. Although BCAR1 orthologs exist only in metazoans as identified by an N-terminal SH3 domain, YxxP motifs, and a C-terminal FAT-like domain, some pre-metazoan transmembrane proteins include several YxxP repeats in their cytosolic region, suggesting that they are remotely related to the BCAR1 substrate domain. Since the tyrosine kinase SRC also has a pre-metazoan origin, co-option of BCAR1-related sequences may have rewired the crka-dependent network to mediate adhesion signals in the metazoan ancestor.

The oncogene v-*CRK* was originally identified in CT10 avian sarcoma virus. Despite the fact that CRK does not encode a tyrosine kinase, v-CRK can increase phosphotyrosine contents in the cell^1^. The name CRK was given as an abbreviation for *C*hicken Tumor 10 *R*egulator of *K*inases. In humans, the *CRK* gene family has two paralogous members, *CRK* and *CRKL (CRK-Like)*, on chromosomes 17p13 and 22q11, respectively. *CRKL* is likely associated with DiGeorge syndrome^2^,^3^, whereas it is yet unclear whether *CRK* has a direct involvement in congenital disorders. Overexpression of CRK or CRKL has been linked to a subset of some cancer types such as ovarian and non-small cell lung cancer^4^. Both *Crk* and *Crkl* are broadly expressed, and are required for normal development in mice^2^,^5^. CRK and CRKL share some functions while also having distinct properties^5^,^6^,^7^,^8^,^9^. One can hypothesize that while functional and structural differences likely resulted from co-option upon their divergence, their shared functions were inherited from their common ancestral gene during evolution.

Both *CRK* and *CRKL* encode adapter proteins consisting of an N-terminal SRC homology 2 (SH2) domain followed by two SH3 domains, SH3n and SH3c^1^. The *CRK* gene generates a full-length product (isoform a; also known as CRK-II) and a shorter product (isoform b or CRK-I lacking the SH3c domain similar to v-CRK). *CRKL* generates only a full-length form in humans. The SH2 domain binds to protein tyrosine kinase (PTK) substrates such as BCAR1 as well as receptor tyrosine kinases (RTK) such as PDGFRA and receptor-associated scaffold proteins such as GAB and DOK^1^. The SH3n domain binds to proline-rich motifs in C3G (RAPGEF1), DOCK180 (DOCK1), and SOS, guanine nucleotide exchange factors (GEFs) for the small G-proteins RAP1, RAC1, and RAS, respectively^1^. These GEFs become activated upon their translocation to the membrane^10^, and loss of *Crk* or *Crkl* impairs activation of corresponding small G-proteins^5^,^6^,^11^,^12^. The protein tyrosine kinase ABL (and the leukemogenic BCR-ABL) is known to bind the CRKL SH3n domain^1^. The tyrosine kinase ABL phosphorylates the CRK/CRKL internal YxxP motif, which inhibits their adapter functions by isolating the SH2 domain^1^,^13^. Unlike SH3n, however, SH3c is a non-canonical SH3 domain that does not bind proline-rich targets^4^. Through these domains, CRK and CRKL can relay PTK-activated signals to downstream signaling mediators, and regulate cell proliferation, migration, and adhesion in response to growth factors as well as cell-matrix interactions^4^.

Some components of the metazoan PTK network initially evolved in unicellular ancestors. The choanoflagellate *Monosiga brevicollis* as well as the filasterea *Capsaspora owczarzaki* may have an elaborate network of PTK-activated signaling, evidenced by an expansive repertoire of tyrosine-specific protein kinases as well as proteins with phosphotyrosine-binding domains such as SH2 and PTB (Phospho-Tyrosine-Binding) domains^14^,^15^,^16^. Inventions of SH2 or SH3 domains individually, however, appear to predate the emergence of PTKs in these pre-metazoan species^17^,^18^.

FAK, SRC, ABL, and their paralogous members play essential roles in integrin-induced signal transduction in mammalian cells^19^,^20^,^21^. Among known PTK families, only the SRC subgroup (TEC, CSK, SRC and ABL) have orthologs in *M. brevicollis*^15^. An FAK ortholog was predicted in *Capsaspora*, but not in choanoflagellates^22^. Integrins and focal adhesion proteins such as paxillin and vinculin were possibly evolved earlier than these kinases^22^. Since communications between the cell and extracellular matrix are vital in multicellular organisms, one important question is how intracellular signaling components have become an integral part of cell-matrix adhesions in the signaling network during pre and post-metazoan evolution.

Among numerous PTK substrates in vertebrate cells, BCAR1 protein, widely known as p130^CAS^ (CRK-associated substrate), was first discovered as a major v-CRK and SRC binding protein^23^. BCAR1 is a focal adhesion protein and has been linked to cell migration, survival, and mechanosensing^24^,^25^,^26^,^27^. BCAR1 has an N-terminal SH3 domain, followed by the substrate domain, Ser-rich domain, and C-terminal FAT-like domain^23^,^26^. Both the SH3 and FAT-like domains are necessary for its localization to focal adhesions as well as for tyrosine phosphorylation of the substrate domain^26^. Src phosphorylates ten or more tyrosine residues out of the 15 YxxP motifs that exist in the substrate domain^25^. Importantly, phosphorylation of multiple tyrosine residues, rather than a single important one, is required for cell migration^25^,^28^. Perhaps as expected, Bcar1 is required for Src-induced cellular transformation^29^, while FAK and ABL also participate in phosphorylation of Bcar1^13^.

Evolution of the signaling network requires co-evolution of interactions after inventions of the primal network components. Our studies demonstrate that the *CRK/CRKL* ancestral gene evolved before the divergence of the metazoan ancestor, and that the SH2 domain of the CRK/CRKL ancestral protein already had the ability to recognize YxxP motifs, an SH2 binding consensus initially discovered with human CRK^30^. While the extant pre-metazoan species do not have a Bcar1 ortholog, we have identified several transmembrane proteins that include many YxxP repeats similar to the metazoan Bcar1 substrate domain. These results suggest a paradigm in which co-option of crk/crkl SH2 binding proteins may have rewired the network connections of highly conserved crk/crkl orthologs in the metazoan ancestor. Since Bcar1 is a focal adhesion protein, our findings present an important implication in the evolution of integrin-based adhesion complex at the dawn of the metazoans.

## Results

### Identification of CRK/CRKL Orthologs

**Figure 1:**
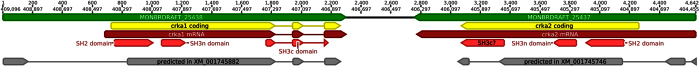
Two Monosiga Genomic Loci and Transcripts Related to CRK and CRKL. The diagram illustrates two loci, MONBRDRAFT_25438 and 25437, in the converging orientation (green bars). The yellow bars indicate the coding sequences of the two mRNA transcripts *crka1* and *crka2* (red bars). The pink bars indicate the predicted SH2 and SH3 domains. The gray bars indicate the exons incorrectly predicted in the provisional models XM_001745882 and XM_001745746. Note that *M. brevicollis crka1* mRNA had a splicing pattern different from XM_001745882 at the 5′ end, and that *M. brevicollis crka2* mRNA was generated without splicing unlike the predicted model XM_001745746. The pattern of exons and introns in *crka1* is supported by the presence of the GT-AG consensus splice site at the boundaries of each intron. Note that *M. brevicollis crka2* does not have an intact SH3c domain, since lack of splicing results in a long intervening sequence that divides conserved sequences and also introduces a stop codon (hence, “SH3c?”; see Supplementary Figs S1 and S6 for more information).

A previous analysis of the *M. brevicollis* genome (release 1.0) predicted that two genetic loci, MONBRDRAFT_25437 and MONBRDRAFT_25438, may encode for *M. brevicollis* orthologs of *CRK*^15^,^31^. These loci are separated by approximately 500 nucleotides in the converging orientation (Fig. 1). We have isolated mRNA transcripts that correspond to these two genetic loci by oligo dT-primed RT-PCR. Our targeted cDNA sequencing revealed two mRNA transcripts. Based on reasoning we will detail in the next section, we termed these transcripts *crka1* and *crka2 (crk/crkl ancestral* 1 and 2). One of the two transcripts identified (*crka1*, MONBRDRAFT_25438) is a product consistent with splicing (Fig. 1). Conversely, the other sequence (*crka2*, MONBRDRAFT_25437) is a product without splicing. Sequence alignments demonstrate that *crka1* and *crka2* differ from the mRNA models posted at NCBI, XM_001745882 and XM_001745746, respectively, in particular in the 5’ end sequences extending into the SH2 domain (Supplementary Figure S1a). In addition, *M. brevicollis crka2* differs in the region corresponding to the SH3c domain due to lack of splicing (Fig. 1; Supplementary Figure S1b).

We have extended our search for orthologous sequences to include several additional species (Supplementary Table S1). To obtain more reliable information than predicted sequences, we have isolated mRNA transcripts or EST plasmids homologous to *CRK/CRKL* from some of these species. Our sequencing results indicate that the predicted sequences posted at NCBI and other public databases frequently include substantial prediction errors. In addition, we have identified a *crk/crkl* ortholog in the genome of the placozoa *Trichoplax adhaerens*. Details are provided in Supplementary Figures S2–S5 as well as in the Methods section.

### Phylogenetic Analysis of CRK and CRKL

**Figure 2:**
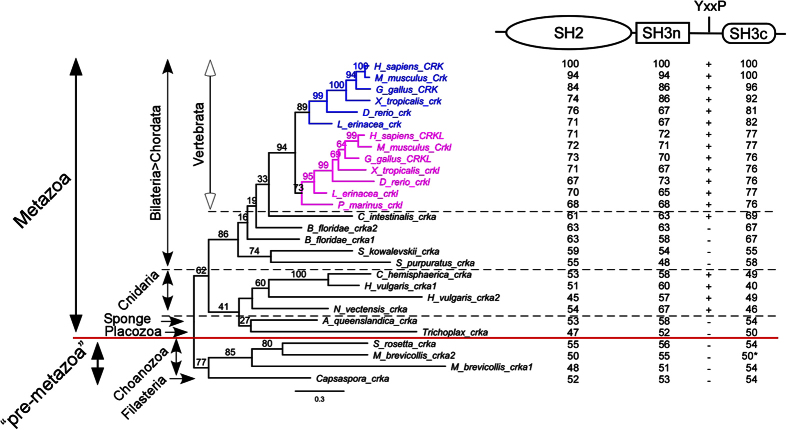
Phylogeny of CRK, CRKL, and Their Orthologs. The maximum likelihood tree was built using RAxML program based on the nucleotide alignment of CRK/CRKL homologs shown in Supplementary Figure S6a. Vertebrate *CRK* and *CRKL* clades are colored blue or magenta, respectively. A bootstrap value is shown at the base of each clade. The scale bar indicates the rate of substitutions. Note that all invertebrate orthologs cannot be distinguished as either CRK or CRKL. Vertebrate *crk, crkl* and their invertebrate orthologs (*CRK-CRKL ancestral; crka*) form distinct clades consistent with their known phyla and subphyla indicated on left. The red line separates the metazoa and premetazoa. The diagram shown at the top right is a simplified structure based on three conserved SRC homology domains. Numbers below each domain indicate the percent identity of pairwise comparisons with human CRK, after all gaps are removed from the nucleotide alignment. YxxP indicates a motif of the internal SH2 binding site. The + or – symbols below YxxP indicates the presence or absence of this motif. The percent identity of *M. brevicollis crka2* SH3c (asterisk) was much lower before removal of gaps (13%), compared with after gap removal (50%). In contrast, the percent identities of *M. brevicollis crka1* SH3c was 44% before gap removal. Details of the alignment as well as additional phylogenetic trees are provided in Supplementary Figures S6 and S7.

Using 28 nucleotide sequences from 19 pre-metazoan and metazoan species, we have generated a confidence score-based multiple sequence alignment (Supplementary Figure S6). All sequences show high levels of nucleotide identities/similarities in the three main regions that correspond to the one SH2 and two SH3 domains (Fig. 2; Supplementary Figure S6b,c). Notably, the reading frame in the alignment was perfectly conserved in the SH2 and SH3n domains across all species. While mammalian CRK SH2 domain includes a proline-rich protruded DE loop^32^, we note that there is a conserved exon-exon junction at the end of the DE loop in vertebrate CRK SH2 domains, with the exception of *L. erineacea crk* (Supplementary Fig. S6b). Interestingly, the DE loop is proline-rich only in mammalian CRK SH2 domains. Contrary to CRK, the CRKL SH2 domain is encoded by a single exon in all vertebrates. The SH3n domain is highly homologous among all species, while the SH3c domain is the most divergent of the three SRC homology domains (Supplementary Figure S6c,d). It is noteworthy that most species including *S. rosetta crka, M. brevicollis crka1,* and *C. owczarzaki crka* have a highly conserved exon-exon junction near the start of the SH3c domain (Supplementary Figure S6d). As *M. brevicollis crka2* is not spliced and lacks this boundary, it is poorly aligned to the SH3c domain of the other species.

These results indicated that our nucleotide sequence alignments were reliable and offered important information such as conserved exon-exon junctions. We therefore proceeded to build phylogenetic trees from the nucleotide alignment in order to further analyze *CRK, CRKL* and their orthologs (Fig. 2; Supplementary Figure S7). Figure 2 shows a best-scored maximum likelihood (ML) tree generated by RAxML along % identities of each SRC homology domains and the presence or absence of an internal YxxP motif (Fig. 2). The tree topology is in general agreement with known evolutionary relationships of the phyla and subphyla. The YxxP motif, a negative regulatory site as a key feature of mammalian Crk^32^, is conserved not only in all vertebrates including *P. marinus*, but also in *C. intestinalis* and Cnidarian species. Since Cnidarians and Bilaterians are considered to have diverged early in metazoan evolution, the internal YxxP motif must have evolved before divergence of the two phyla.

We also used Bayesian inference as another tool to build phylogenetic trees (Supplementary Figure S7a). Bayesian analysis generates a consensus tree based on the mean branch lengths from numerous MCMC generations. Therefore, apparent multifurcations in the Bayesian tree and low bootstrap numbers in the ML tree suggest that the coding sequences of CRK/CRKL orthologs alone may be statistically insufficient to call a definitive evolutionary order at the base of chordates as well as between basal metazoans. We also generated phylogenetic trees from a protein alignment (Supplementary Figure S7b,c). However, both ML and Bayesian protein trees show misplacements of some species, indicating that nucleotide alignments offer more precise information than protein alignments for our phylogenetic analysis of crk/crkl ortholog sequences.

Despite minor differences, these trees indicate that all vertebrate species have two distinct genes, *CRK* and *CRKL*, with an exception of *P. marinus*, which contains only a *crkl* homolog (Fig. 2; Supplementary Figure S7). The classification of this homolog as *CRKL* rather than *CRK* is supported by the fact that *P. marinus crkl* is localized close to *klhl22*, similar to the linkage between human *CRKL* and *KLHL22* found in 22q11 (Supplementary Table S7). Therefore, the divergence of *CRK* and *CRKL* likely occurred before the common ancestor of the vertebrate subphylum. In contrast, invertebrate *CRK/CRKL* orthologs could not be distinguished as closer to either *CRK* or *CRKL* before their divergence. Therefore, we introduce a new term, *crka (crk/crkl* ancestral), to refer to invertebrate *CRK/CRKL* orthologs and avoid identifying them as either *CRK* or *CRKL*. The presence of *crka* not only in basal metazoans such as the placozoa *Trichoplax adhaerens* and sponge *Amphimedon queenslandica* but also in the pre-metazoan species *Salpinogoeca rosetta, Monosiga brevicollis*, and *Capsaspora owczarzaki* clearly indicates that the *CRK* gene family has a pre-metazoan origin.

### Structure of the SH2 Domain of *Monosiga brevicollis* crka1

**Figure 3:**
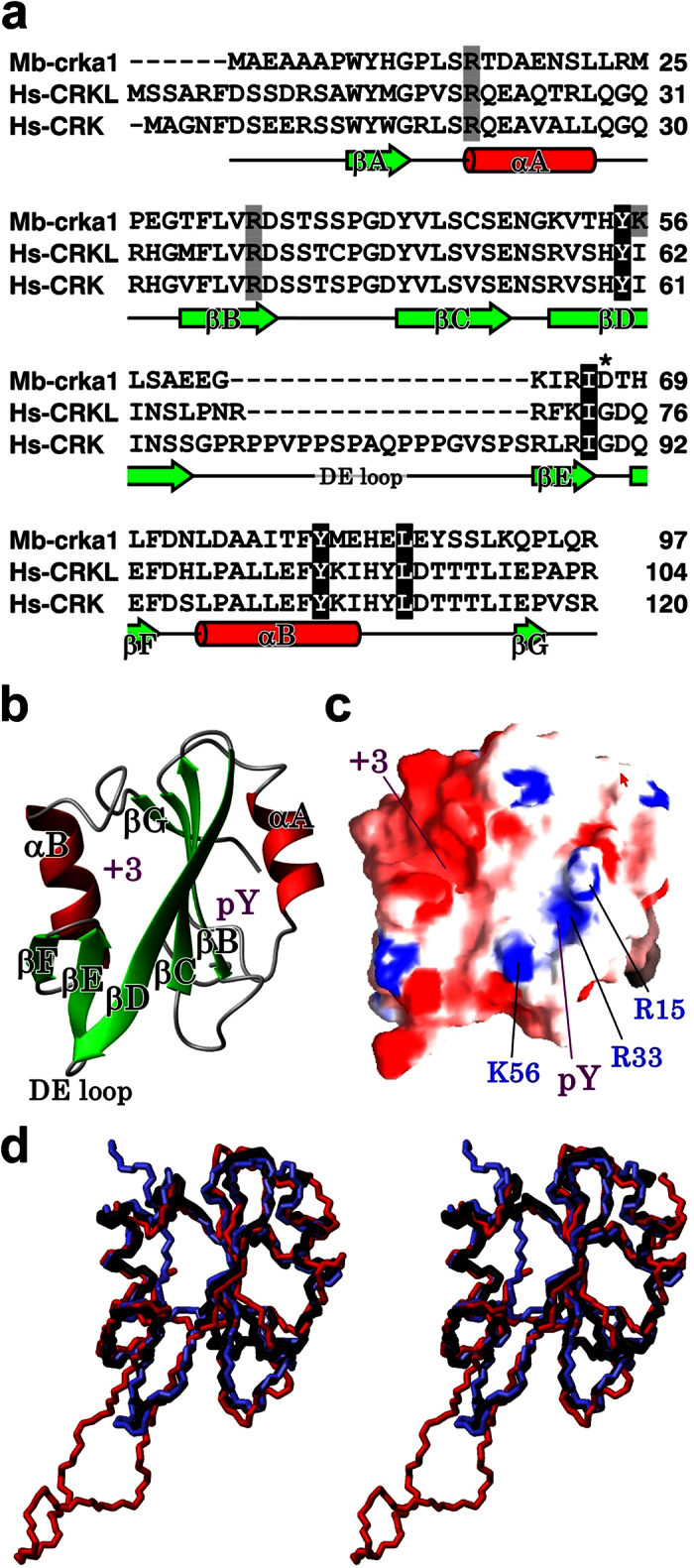
Solution Structure of the *M. brevicollis* crka1 SH2 Domain. (**a**) The panel shows an amino-acid sequence alignment based on the solution structures of the SH2 domain of *M. brevicollis* crka1 (Mb-crka1), human CRKL and CRK (Hs-CRKL and Hs-CRK). Secondary structure elements of Mb-crka1 are indicated below the alignment. The basic residues surrounding the phosphotyrosine binding site and the hydrophobic residues located at the +3 residue binding pocket are indicated as gray and black shades, respectively. An asterisk indicates the *i*+1 position of β turn between βE and βF. (**b,c**) Panels b and c show ribbon and surface models of the mean structure of the Mb-crka1 SH2 domain, respectively. The surface model also shows the electrostatic potential. The basic and acidic residues are shown in blue and red, respectively. The phosphotyrosine binding site (pY) and the +3 residue binding site (+3) are indicated by purple letters. Consistent with previous structural studies of the mammalian CRK SH2 domain, two highly conserved basic residues (R15 and R33) lie in the pY binding pocket and interact with the phosphate group of phosphotyrosine in ligand proteins. In Mb-crka1 SH2, another basic residue (K56) also faces the pY binding pocket. This residue is conserved among the choanozoa crka proteins, but not in the other species (Supplementary Figure S6b). Another pocket lies on the other side of the three anti-parallel beta sheets and appears to be structurally sufficient to accommodate a proline residue identified in mammalian CRK/CRKL SH2 binding motif (the +3 position from a tyrosine residue). (**d**) A wire model of Mb-crka1 SH2 domain (black) is shown in stereo views, overlaid with human CRK and CRKL SH2 domains based on PDB IDs 2EO3 and 2EYV (red and blue, respectively). While human CRK has an extruded DE loop (see also Panel a), the other parts of the SH2 backbones are well-aligned.

To gain insight into functional evolution, we have determined the solution structure of the SH2 domain of *M. brevicollis* crka1 (Fig. 3; Supplementary Table S2). This domain consists of two α helices separated by three anti-parallel β sheets at the middle of the domain (Fig. 3b). Two highly conserved key basic residues (R15 and R33) are located in a pocket on one side of the anti-parallel β sheets (Fig. 3a–c). As they are known to provide essential interactions with the phosphate group of the ligand protein, it is likely that the crka1 SH2 domain is a functional phosphotyrosine binding domain. Interestingly, another basic residue (K56) faces the phosphotyrosine binding pocket in the *M. brevicollis* crka1 SH2 domain. This lysine residue is conserved exclusively in choanoflagellates (Supplementary Figure S6b). In mammalian CRK and CRKL SH2 domains, a hydrophobic pocket interacts with a proline residue at the +3 position from the phosphotyrosine found in many ligand proteins^30^. We have identified four hydrophobic residues, Y55, I66, Y81, and L86, in a putative proline-binding pocket on the other side of the anti-parallel β sheets conserved in the sequence alignment (Fig. 3a). Overall, the *M. brevicollis* crka1 SH2 domain superimposes closely with those of human CRK^32^,^33^ and CRKL^9^ (Fig. 3d). Our results therefore suggest that the *M. brevicollis* crka1 SH2 domain is capable of interacting with phospho-YxxP similar to its mammalian counterparts.

While the overall domain structure appeared to be highly conserved, we have noted an unusual residue, aspartic acid D67, at the *i*+1 position of the β turn in the EF loop in the *M. brevicollis* crka1 SH2 domain, where other species have a highly conserved glycine residue (Supplementary Figure S8c). Curiously, the program PROCHECK mapped D67 in a “disallowed” region in a Ramachandran plot unless the residue was manually converted to a glycine (Supplementary Figure S8b). To verify the local structure around the D67 residue, we determined the ^3^J_HNHA_ coupling constant by HNHA experiment (Supplementary Table S3). The coupling constant is close to the predicted value of type I’ or II’ β turns, rather than other standard β turns. However, we could not firmly determine a β turn classification, since the signal intensity in the HNHA spectrum was too low to determine the coupling constant of the *i*+2 residue, T68, and its backbone dihedral angles. Nevertheless, ^3^J_HNHA_ analysis indicates that while the *M. brevicollis* crka1 SH2 domain has an unconventional amino acid in the EF loop, the β turn structure itself is conserved.

### Binding of Phosphotyrosyl Peptides to *Monosiga brevicollis* crka1 SH2 Domain

**Figure 4:**
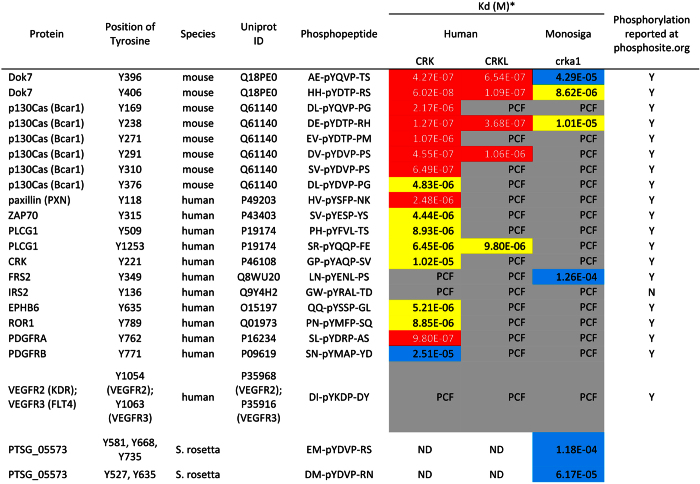
ITC Reveals Physical Binding of Phospho-YxxP Peptides to Monosiga crka1 SH2 Domain. Values indicate the binding affinity as a dissociation constant (Kd). Kd values are arbitrarily categorized as high affinity (Kd < 3 μM, red), moderate affinity (Kd between 3–20 μM, yellow), or low affinity (Kd > 20 μM, blue). Kd values are not shown in cells shaded gray due to poor curve fitting (PCF). See titration plots in Supplementary Figure S9. ND, not determined.

The structural analysis above has predicted that the *M. brevicollis* crka1 SH2 domain may recognize the YxxP binding consensus of mammalian CRK and CRKL SH2 domains. To address this issue further, we have used isothermal titration caloriometry (ITC) to determine physical binding of the *M. brevicollis* crka1 SH2 domain with selected 21 phosphotyrosyl peptides previously reported to associate with mammalian CRK or CRKL SH2 domains^25^,^34^,^35^,^36^,^37^,^38^,^39^ (Fig. 4). As expected, most peptides tested show specific-binding to either or both human CRK and CRKL SH2 domains. Significantly, two phosphotyrosyl peptides from *M. musculus* Dok7 (Y406) and Bcar1 (p130Cas, Y238) bind the crka1 SH2 domain with K_D_ values ranging 8–10 μM (Fig. 4; Supplementary Figure S9). These results confirm the prediction from our structural analysis above and suggest that the binding preference of CRK/CRKL SH2 domains is remarkably conserved throughout evolution.

### *Monosiga brevicollis* crka1 binds to mammalian Bcar1 and Rapgef1 in a Heterologous System

**Figure 5:**
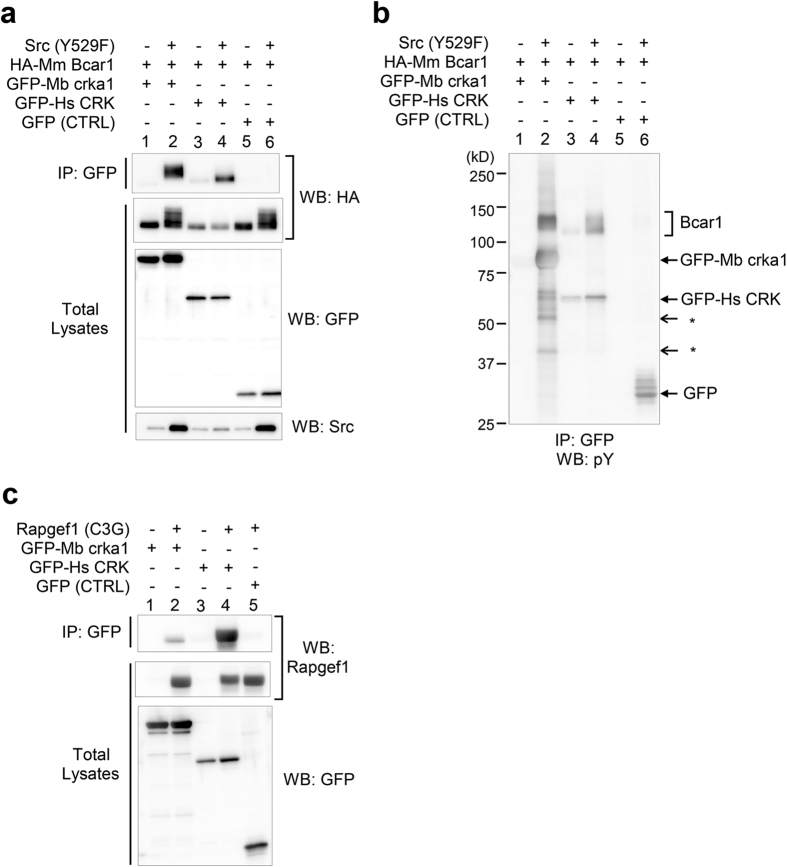
Association of *Monosiga brevicollis* crka1 Protein with Mammalian Bcar1 and Rapgef1. *M. brevicollis* crka1 associated with an HA-tagged mouse Bcar1 (HA-Mm Bcar1) when an activated mouse Src (Y529F) was co-expressed in a heterologous system (lane 2). Human embryonic kidney 293 cells (HEK293) were transfected with combinations of plasmids as indicated. *M. brevicollis* crka1 and human CRK were fused to an enhanced GFP at their N-terminus to enable immunoprecipitation (IP) with anti-GFP antibodies. Lanes 1 and 3 were samples from control groups without active Src, showing little co-precipitation of HA-Bcar1. Lane 4 was a positive control with human CRK instead of Mb crka1. Lanes 5 and 6 were negative controls in which GFP without human CRK or Mb crka1 SH2 domain was expressed. The amount of Bcar1 co-precipitation could not be directly compared between Mb crka1 and Hs CRK, since Hs CRK appeared to reduce protein levels of transfected HA-Mm Bcar1 and Src (reproducible in three experiments; see Western blots of cell lysates for HA and Src). Mb crka1 binds to multiple proteins phosphorylated by active Src. Phosphotyrosine contents were probed by an anti-phosphotyrosine antibody in the same immunoprecipitates used in panel a. While Bcar1 was the major protein that bound to Mb crka1 and Hs CRK (lanes 2 and 4), additional tyrosine-phosphorylated proteins of unknown identities (*) also associated with Mb crka1 (lane 2). We noted that GFP-fusion proteins and GFP itself were also phosphorylated by active Src (lanes 2, 4, and 6). (**a**) *Monosiga brevicollis* crka1 can associate with the CRK SH3 binding protein RAPGEF1 (also known as C3G), albeit at a lower level than that of human CRK (lanes 2 and 4). A heterologous system was designed to co-express RAPGEF1 with GFP-tagged Mb crka1 or Hs CRK in HEK293 cells as above.

As the biophysical and biochemical experiments above suggest that Bcar1 may bind to *M. brevicollis* crka1, we constructed a heterologous system to test their association in the presence or absence of activated Src in human embryonic kidney 293 cell line (Fig. 5). Although the amount of association could not be directly compared due to the differential effects of crka1 and CRK on the protein expression of Src and Cas, HA-tagged Bcar1 was co-precipitated efficiently with *M. brevicollis* crka1 when a constitutively active Src was co-expressed (Fig. 5a). Interestingly, *M. brevicollis* crka1 associated with a broader range of proteins besides HA-tagged Bcar1 as compared to human CRK (Fig. 5b). In addition, we found that *M. brevicollis* crka1 physically associated with the major CRK/CRKL SH3 binding protein RAPGEF1 (C3G) in 293 cells, although the level of association was much lower than that of human CRK (Fig. 5c). These results therefore demonstrate that *M. brevicollis* crka1 has biochemical properties similar to that of human CRK, despite their large distance in evolutionary time.

### Search for Bcar1 Orthologs

Our results above have hinted that possible co-evolution of Bcar1 may offer insight into the crka SH2-mediated network during the pre-metazoan-metazoan evolution. To address this hypothesis, we first searched bcar1 orthologs in several species. Our tblastn search identified bcar1 orthologs in the hemichordate *Saccoglossus kowalevskii* and two Cnidarians, *Hydra vulgaris* and *Nematostella vectensis* based on high similarities in the N-terminal SH3 domain, Ser-rich domain, and C-terminal FAT-like domain (Table 1; Supplementary Figure S10a). We have also identified a *bcar1* ortholog in the sponge *Amphimedon queenslandica* using similar strategies, although high similarities were confined only to the SH3 and FAT-like domains. Although the placozoa *Trichoplax adhaerens* showed a high degree of similarity in the SH3 domain, the other domains showed low overall sequence similarities (Table 1; Supplementary Figure S10a and Table S4). Despite relatively low similarity scores, the presence of several YxxP motifs, ranging 6–25 repeats depending on the species, is a highly conserved feature of the substrate domain (15 motifs in *Homo sapiens* and *Mus musculus*; 6 in *A. queenslandica* and *T. adhaerens*). However, similar BLAST search strategies did not identify a *bcar1* ortholog in the choanozoa or *C. owczarzaki* genome (Table 1). These findings suggest that the major CRK/CRKL binding partner BCAR1 likely evolved during or before the emergence of the common metazoan ancestor, although it may have been lost in the extant pre-metazoan genomes.

### Pre-metazoan crka SH2 Binding Partners

**Figure 6:**
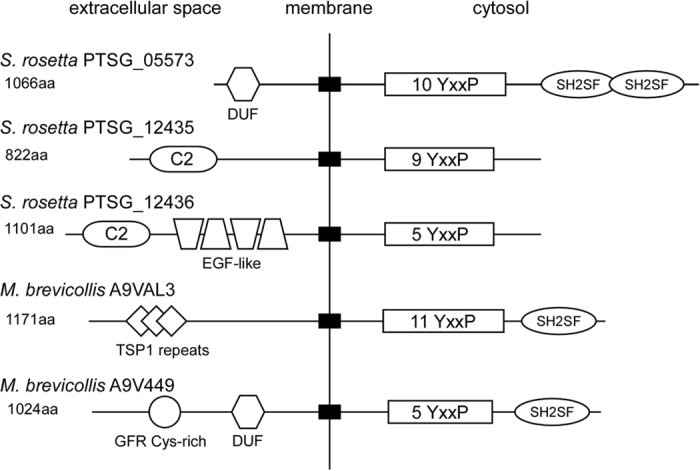
Predicted Pre-metazoan Proteins Include Several YxxP Motifs. The diagrams illustrate two groups of predicted/uncharacterized proteins: 1) three *S. rosetta* proteins identified by BLASTP with the Bcar1 and Dok7 peptide sequences that bind to the *M. brevicollis* crka1 SH2 domain (Table 1; Supplementary Table S5 and 2) two *M. brevicollis* proteins identified by a profile hidden Markov model constructed from the metazoan bcar1 alignment shown in Supplementary Figure S10a without the SH3 domain. Despite the fact that they are identified by two independent methods, all 5 proteins are transmembrane proteins with several YxxP motifs in the predicted cytosolic region. Closed boxes across the membrane indicate predicted transmembrane regions. PTSG_05573 has an extracellular DUF5011 domain that has not been assigned to known functions. PTSG_12435 and 12436 have a C2 domain (Ca^2+^-dependent phospholipid binding domain) after the N-terminal signal peptide. In PTSG_12436, repeats of EGF-like domains likely mediate protein-protein interactions. In *M. brevicollis* A9VAL3, the extracellular region includes three thrombospondin type 1 repeats (TSP1) that may mediate cell-cell interactions or ligand-receptor binding. In *M. brevicollis* A9V449, the extracellular region includes a cysteine-rich growth factor receptor (GFR) domain found frequently in mammalian growth factor receptors such as EGFR, PDGFR and IGF1R, as well as a DUF domain. PTSG_05573, A9VAL3, and A9V449 have SH2-like domains (SH2SF: SH2 domain superfamily) near the C-terminus. A9VAL3 and A9V449 are UniProt accession numbers, while PTSG_05573, 12435 and 12436 are current gene symbols/IDs.

The bcar1 substrate domain is not easily identified by BLAST using the whole domain sequence, even in metazoan species. Therefore, we searched RefSeq protein databases instead with the two mouse peptide sequences that showed significant affinities to *M. brevicollis* crka1 SH2 domain (Table 1). This search yielded BCAR1 as the only protein with “multiple hits” in the human and *Mus musculus* RefSeq protein databases (Supplementary Table S5). Using this strategy, we have identified three putative proteins that contain multiple YxxP motifs in the choanoflagellate *Salpingoeca rosetta* (Supplementary Table S5). Upon closer examinations, we have found that PTSG_05573, 12435, and 12436 encode putative transmembrane proteins that include several YxxP motifs in the cytoplasmic region (Fig. 6). These YxxP motifs, such as in PTSG_05573, aligned to the metazoan bcar1 substrate domain (Supplementary Figure S10b). Unlike metazoan bcar1 orthologs, however, these proteins did not have an SH3 domain or other bcar1-like domains (Table 1). To provide evidence that these YxxP motifs may serve as pre-metazoan crka SH2 binding sites, we performed ITC assays with *M. brevicollis* crka1 SH2 and two phosphopeptide sequences, EM-pYDVP-RS and DM-pYDVP-RN, which overlap 5 out of the 10 YxxP motifs in PTSG_05573. We found that these peptides bound to the *M. brevicollis* SH2 domain (Table 1). Since biochemical and structural properties are remarkably conserved in the SH2 domain between human CRK/CRKL and *Monosiga* crka1, one may speculate that *S. rosetta* crka likely shares such conserved protein properties, which may permit binding to PTSG_05573. In addition, the multiplicity of YxxP motifs likely boost the protein-protein association, despite a low affinity at each site. BLASTP did not identify proteins containing the two-peptide sequences in *Monosiga brevicollis* or *Capsaspora owczarzaki*. BLASTP could also not identify orthologs of these *S. rosetta* genes in the other pre-metazoan species.

To identify sequences distantly related to the metazoan bcar1 in pre-metazoans, we have constructed profile hidden Markov models^40^ (profile HMMs) using the bcar1 protein alignment of 10 metazoan species shown in Supplementary Figure S10a. Since the SH3 domain is much more highly conserved than other regions, our preliminary search with an HMM constructed from the whole alignment was overly represented by SH3 domain-containing proteins, none of which included regions similar to the bcar1 substrate domain. Therefore, alternative profile HMMs were constructed from the alignment without the SH3 domain. With this strategy, we identified two proteins, A9VAL3 (XP_001749805, MONBRDRAFT_11892) and A9V449 (XP_001747564, MONBDRAFT_27039) in the *M. brevicollis* UniProt database at E-values of 0.00047 and 0.0032, respectively. Although there were a few hits in *S. rosetta* and *C. owczarzaki*, they had E-values above 0.05 and contained no YxxP motifs.

The five proteins identified in *S. rosetta* and *M. brevicollis* have notable features (Fig. 6). All of them are transmembrane proteins. In addition to several YxxP repeats, three proteins have a cytosolic region with one or two C-terminal SH2-like domains (SH2 domain superfamily). In the extracellular region, A9VAL3 has three thrombospondin type 1 repeats, whereas A9V449 has a cysteine-rich growth factor receptor-like domain and DUF5011 domain (domain of unknown function). PTSG_05573 has a DUF5011 in its extracellular region, but it does not have other domain-like, pattern-identifiable features. Although chained HMMs may introduce a higher risk of false findings, we constructed another profile HMM from the five proteins. The second HMM identified several transmembrane proteins, subsets of which included YxxP repeats in *M. brevicollis, S. rosetta*, and *Capsaspora* (Supplementary Table S6). These proteins may represent highly divergent groups of pre-metazoan-specific transmembrane proteins which harbor sequences remotely related to the metazoan bcar1 substrate domain.

## Discussion

Based on the refined sequence information we obtained, our analysis suggests that the divergence of vertebrate *crk* and *crkl* genes may have taken place at or before the divergence of the vertebrate ancestor. While *P. marinus* contains only a *CRKL* ortholog, all other vertebrates have two distinct genes identified as *CRK* and *CRKL* (Fig. 2). Susumu Ohno originally proposed a revolutionary idea that whole genome duplications (WGDs) had been an important driver responsible for the complexities of metazoan species^41^. The 2R hypothesis assumes that two rounds of WGDs (2R) occurred around the speciation of the vertebrate ancestor. Recent studies of the most primitive vertebrates, the cyclostomes *P. marinus* and hagfish, suggest that 2R occurred before the divergence of the vertebrates^42^,^43^. WGDs generate paralogs called ‘ohnologs’ evidenced by chromosomal synteny, while many such paralogs undergo sequence degeneration followed by gene loss^44^. Chromosomes 17q13 and 22q11, to which *CRK* and *CRKL* are localized in humans, show extensive synteny (Supplementary Table S7). Our phylogenetic analysis is consistent with the hypothesis that the last of the 2R occurred before the divergence of the vertebrate ancestor.

One notable difference between vertebrate *CRK* and *CRKL* genes is the fact that the SH2 domain is split over two exons with a junction after the DE loop in *CRK* orthologs, while it is encoded by a single exon in *CRKL* orthologs. It is possible that either gain of a new intron in *CRK* or loss of a previously existed intron in *CRKL* occurred after the WGD. When we consider these possibilities with highly divergent sequences in the DE loop, it seems more plausible that CRK gained a new intron that may have included alternative splice donor sequences. If a splicing slippage occurred before the divergence of vertebrate species after the WGD, we would anticipate more conserved sequences in the DE loop. Thus, the highly divergent CRK DE loop may be explained by the possibility that splicing slippages occurred independently in multiple vertebrate lineages.

Outside the vertebrates, all primitive species have one or more *crka* genes. It cannot be determined if these orthologs are more closely related to either *CRK* or *CRKL*. We have noted that *Ciona intestinalis* has two additional model loci related to the *crka* gene. One is immediately adjacent to *crka* on chromosome 12 (XM_002130761), and the other is on chromosome 5 (XM_009860485). While these two are also annotated as *crk-like* at NCBI, our alignment and phylogenetic analysis produce long-branch attraction artifacts if they were included (not shown). In addition, a closely related urochordate, *Ciona savignyi,* has only one ortholog that corresponds to *crka* in the Ensembl database (ENSCSAVG00000010659). Hence, we excluded the two additional loci from the current study, and called them *crkb* and *crkc*, respectively. It is possible that the *crkb* and *crkc* loci accumulated extensive sequence degeneration after they were duplicated in the *C. intestinalis* lineage. It is noteworthy that *C. intestinalis* chromosome 12 contains several genes that are orthologous to human chromosomes 17 or 22 and to *Xenopus tropicalis* genomic scaffolds to which *crk* and *crkl* are localized (Supplementary Table S7). It has been proposed that the 2R occurred after the split of *C. intestinalis*^45^. Therefore, together with the topology of our phylogenetic trees, we speculate that *C. intestinalis* chromosome 12 retains a block of the common ancestor chromosome from which a WGD may have generated vertebrate *crk* and *crkl*.

**Figure 7:**
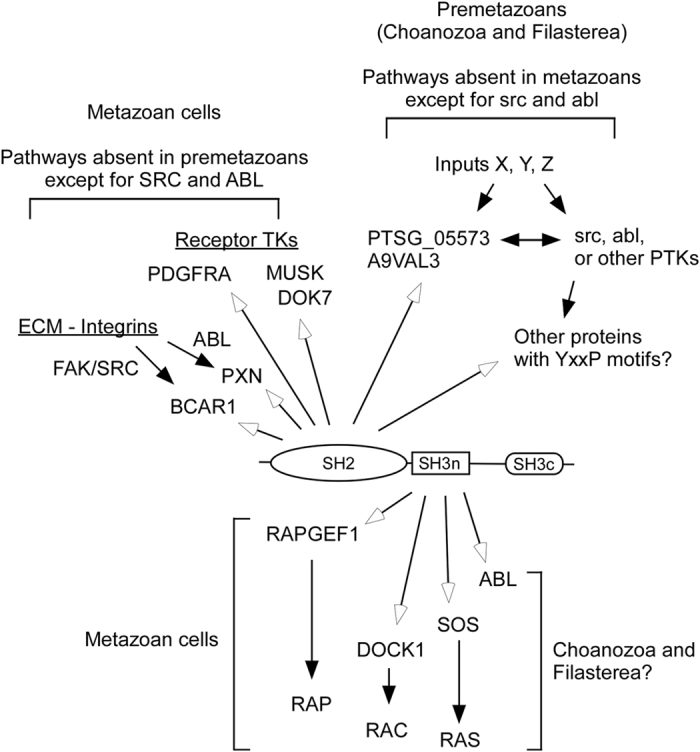
Proposed Model of the CRK/CRKL Signaling Network in Metazoans and Pre-metazoans. In mammalian cells, CRK and CRKL have been assigned to pathways induced by growth factors or extracellular matrix (ECM) proteins. The open arrows indicate the physical association of CRK-CRKL orthologs to their binding partners, while the closed arrows indicate the flow of signals when pathways are activated. Receptor tyrosine kinases (RTK) and intracellular tyrosine kinases such as PDGFRA, MUSK, FAK (PTK2), SRC, and ABL phosphorylate their substrates, which the CRK/CRKL SH2 domain may bind. Many such substrates localize to the plasma membrane as well as to adhesion structures such as focal adhesions in vertebrate cells. Although choanozoa and filasterea have many TKs, integrin-based signaling complexes or known growth factor RTKs are absent. Pre-metazoan CRK/CRKL orthologs likely respond to transmembrane signals that do not exist in metazoan cells. Since SRC and ABL orthologs exist in pre-metazoan species, they may phosphorylate crka SH2 binding proteins. On the other hand, orthologs of major CRK/CRKL SH3n binding proteins such as ABL, DOCK1, and SOS exist in choanoflagellates and *Capsaspora* (see Supplementary Table S7).

We have demonstrated that the common ancestry of *CRK* and *CRKL* can be traced back to a pre-metazoan origin. As adapter proteins, pre-metazoan crka proteins likely mediate protein-protein interactions through their SRC homology domains in similar ways to their metazoan counterparts (Fig. 7). In basal metazoan and pre-metazoan species, we identified orthologs for a few major CRK/CRKL SH3n-binding proteins, some of which have proline rich motifs with which SH3n may associate (Supplementary Table S8). On the other hand, we have little evidence of orthologs for known CRK/CRKL SH2-binding partners in pre-metazoan species (Supplementary Table S8).

Our current study has offered evidence that the focal adhesion protein *BCAR1* likely evolved during or before speciation of metazoa, while previous studies suggested that some focal adhesion proteins such as paxillin (pxn) were present in the pre-metazoan species^22^. Paxillin is phosphorylated by FAK, ABL and SRC in response to integrin-mediated cell adhesion and growth factor-mediated signaling in mammalian cells^46^. Like Bcar1, paxillin is essential for mouse development^29^,^47^. Mammalian paxillin proteins have two YxxP motifs (Y31 and Y118) as primary phosphorylation sites at the N-terminus, which bind to the SH2 domain of CRK and CRKL when phosphorylated^46^. However, conserved YxxP motifs are not found in paxillin orthologs in *M. brevicollis* (XP_001742769) and *C. owczarzaki* (XP_004345254) nor in two basal metazoans, *Amphimedon queenslandica* and *Trichoplax adhaerens* (XP_011402803 and XP_002115001, respectively). The conserved YxxP motifs exist in zebrafish paxillin (XP_005172507), but not in *Hydra vulgaris, S. kowalevskii,* or *Ciona intestinalis* (XP_002168161, XP_006811982, and XP_002127320, respectively). On a related note, paralogous members of the human paxillin family, leupaxin (LPXN) and HIC5 (TGFB1I1), do not have YxxP motifs that correspond to paxillin Y31 and Y118. Therefore, it is likely that Bcar1 has been a major player in mediating metazoan PTK signals until co-option has recruited paxillin to participate in networking with CRK and CRKL at focal adhesions in vertebrates.

In summary, our results underscore a high degree of conservation in crk/crkl orthologs, thus suggesting the importance of this adapter protein family in the signaling network throughout evolution. Our current evidence links the function of the *M. brevicollis* crka1 SH2 to the conserved binding consensus phospho-YxxP, although it does not exclude the possibility that pre-metazoan crka SH2 domains may have distinct binding preferences. We speculate that a recruitment of crka to bcar1 may have become an integral mechanism to mediate cell community-based signaling in the metazoan ancestor likely through co-option of bcar1 as a multi domain signaling center (Fig. 7). In pre-metazoans, conserved functions of crka likely link the signaling complex to the membrane through pre-metazoan-specific transmembrane proteins. FAK, SRC, and ABL are known to phosphorylate YxxP motifs in mammalian cells^1^,^4^, and their orthologs exist in choanoflagellates and Capsaspora^13^,^15^,^22^. To date, their pre-metazoan substrates remain unknown, however. Future studies are warranted to determine whether and how the connectivity shift of crk/crkl orthologs via co-option of bcar1-related sequences may have contributed to the evolution of multicellularity or other metazoan-specific cell functions.

## Methods

### Identification of Sequences Closely Related to Mammalian *CRK* and *CRKL*

CRK and/or CRKL homologs have been annotated in RefSeq databases for several species, although some sequences are still listed as “provisional” at NCBI (http://www.ncbi.nlm.nih.gov/; Supplementary Table S1). The annotated sequences available at NCBI were also compared with the Ensembl databases (http://www.ensembl.org/). In these sequences, we identified a significant difference between the *Xenopus tropicalis crkl* homolog sequences available at NCBI and Ensembl. We chose the sequence from Ensembl as it was more in line with the criteria described below. In the species in which genes are not fully annotated, tblastn was used to identify candidate nucleotide sequences (http://amphioxus.icob.sinica.edu.tw/ for the cephalochordate *B. floridae*; personal communication with Chris Lowe for the hemichordate *S. kowalevskii*; http://skatebase.org/ for the little skate *Leucoraja erinacea*; http://cnidarians.bu.edu/stellabase/ for the sea anemone *Nematostella vectensis*; and http://blast.ncbi.nlm.nih.gov/Blast.cgi for other species). When available, genomic information was also used to confirm RNA sequences. In species in which database information provided no or partial information, we sequenced EST plasmids to obtain full sequence information, or isolated cDNA using RT-PCR from RNA samples obtained from the organism.

Four independent cDNA clones were isolated from a pool of embryonic RNA preparations of the sea lamprey *Petromyzon marinus*. All four cDNAs are 90 bases longer in the coding region than the annotated/provisional *P. marinus crkl* sequence available at Ensembl, which would result in additional 30 amino acids between the SH2 and SH3n domains when translated. The missing bases in the annotated *crkl* sequence can be attributed to a stretch of nucleotide ambiguities that still exists in the corresponding region of in the genomic contig, likely including an additional small exon (Supplementary Figure S2). The four sequences we obtained were independent and unique clones from a single gene, as they differ at synonymous sites or one amino acid insert within the open reading frame.

We have also used full-length EST plasmids to obtain transcripts of *Crk/Crkl* homologs in the urochordate *Ciona intestinalis*, which turns out to be identical to the published model sequence XM_002130671 for the coding sequence. We have also sequenced two distinct full-length EST plasmids for the *crk/crkl* homologs in the cephalochordate *Branchiostoma floridae* (Supplementary Figure S3). These two transcripts are similar but mapped to non-overlapping genomic scaffolds (Supplementary Table S1; mapping data not shown). Thus, they are transcribed from two independent loci, rather than transcripts produced by alternative splicing or polymorphisms from a single gene. The *crk/crkl* transcript we obtained from the hemichordate *Saccoglossus kowalevskii* is different from the sequence annotated as ‘*crk-like protein like*’ at NCBI (XM_006822248) by 147 bases corresponding to approximately half the SH2 domain (Supplementary Figure S4). Multiple sequence alignment indicates that our *S. kowalevskii* ortholog is more similar to the mammalian Crk/Crkl SH2 domain (Supplementary Figure S4). Hence, the sequence we obtained is used in our phylogenetic analysis.

While we did not find annotated sequences for *crk* or *crkl* in the placozoa *Trichoplax adhaerens*^48^, we have found a genomic contig (ABGP01000065) that contains *crk/crkl* homologous sequences by tblastn over its WGS database with the sea anemone *Nematostella vectensis* homolog as a query. From this genomic sequence, we have predicted a coding sequence that aligns well with other *crk/crkl* homologs (Supplementary Figure S5).

Total RNA samples were obtained from Nicole King for the choanoflagellate *Monosiga brevicollis*, and Marianne Bronner for the sea lamprey *Petromyzon marinus*. EST plasmids were obtained from Nori Satoh for the tunicate *Ciona Intestinalis* and the amphioxus *Branchiostoma floridae*, and from Chris Lowe for the hemichordate *Saccoglossus kowalevskii*. We chose only the sequences that have all three characteristic domains found in mammalian Crk and Crkl: one SH2 followed by two SH3 domains. When multiple transcripts (isoforms) were identified from a single gene, we chose the longest transcript for phylogenic analysis.

### RT-PCR and Sequencing

Reverse transcriptase (Superscript II, Lifetechnologies) was used with oligo dT primers to generate cDNA pools from total RNA isolated from *P. marinus* or *M. brevicollis* per manufacturer’s protocol. *Crk/Crkl* homologs were then amplified by high-fidelity PCR with PrimeSTAR (TAKARA) using specific-primers designed for the Gateway cloning system (Invitrogen). Isolated cDNA fragments as well as full-length EST plasmids were sequenced for both upper and lower strands at the University of Chicago Sequencing Core Facility.

### Phylogenetic Analysis

GUIDANCE2 server was used with MAFFT alignment options (\-\-genafpair \-\-maxiterate 1000) to generate iterative nucleotide and protein alignments and to provide a confidence score for each aligned column^49^,^50^. Poorly aligned columns below a confidence score of 0.93 were removed before tree building. All sequences scored higher than a default sequence cutoff score of 0.6. For maximum likelihood (ML) analysis, raxmlGUI version 1.5b1 was used to interface a parallel-threaded RAxML^51^,^52^. A preset of rapid bootstrap and best tree search was used with the general time reversible (GTR) substitution model + gamma or with a fixed cost matrix of Whelan and Goldman (WAG) + gamma for RAxML nucleotide or protein tree generations, respectively. For Bayesian analysis, an MPI version was compiled from the MrBayes source code (version 3.2.6), and was used to run a Metropolis-coupled Markov chain Monte Carlo (MCMC) analysis for 1,400,000 generations with 1 cold and 3 heated chains^53^. A relative burn-in was set at 25% of total generations. Bayesian nucleotide and protein “consensus” trees were generated using GTR or WAG with 8 gamma categories, respectively. For both RAxML and MrBayes, 8 processor cores were assigned to run parallel computations. When multiple sequence alignments were performed without tree generations for bcar1 orthologs and related sequences, MAFFT (v7.271) E-INS-i iterative algorithm was used with a gap extension penalty of 0 (--ep 0), suited to identify conserved motifs in long poorly aligned regions^50^. To search bcar1-related sequences poorly conserved in pre-metazoan species, a Linux version of HMMER v3.1b2 (hmmer.org) was used to construct profile hidden Markov models^40^ (HMMs) from the protein alignment shown in Supplementary Figure 10a without SH3 domain or similar alignments of pre-metazoan transmembrane proteins. Searches using profile HMMs were conducted against UniProt protein databases downloaded from www.uniprot.org (SALR5, MONBE, and CAPO3 protein databases for *S. rosetta, M. brevicollis*, and *C. owczarzaki*, respectively). Geneious program suite (Biomatters) was used to visualize trees and alignments.

### Protein Synthesis

The SH2 domains of human CRK, CRKL, and *M. brevicollis* crka1 were generated initially as polyhistidine-tagged proteins as described^54^. Recombinant proteins were purified with HisTrap HP columns (GE Healthcare). His-tag was then cleaved from the protein with Tobacco Etch Virus protease and removed by the affinity column. After His-tag removal, the synthesized protein included additional seven amino acids (GSSGSSG) at the N-terminus as a byproduct of the production process.

### NMR and Structure Analysis

For structure determinations, the SH2 domain of *M. brevicollis* crka1 was synthesized as above, then labeled with ^13^C and ^15^N using the dialysis mode of the *Escherichia coli* cell-free protein synthesis system^55^. The NMR sample containing 1.1 mM labeled proteins in the NMR buffer (20 mM ^2^H_11_ Tris–HCl buffer, pH 7.0, 100 mM NaCl, 0.02% (w/v) NaN_3_, and 1 mM ^2^H_10_ dithiothreitol) was prepared with Vivaspin 2 Concentrator MWCO 5000 PES (Sartorius). All NMR experiments were performed at 298 K on 600, 700, or 800 MHz Avance spectrometers (Bruker) equipped with CryoProbes. Acquired NMR spectra for the chemical shift assignments and the distance constraints were ^1^H-^15^N HSQC, ^1^H-^13^C HSQC, HNCO, HN(CA)CO, HNCA, HN(CO)CA, HNCACB, CBCA(CO)NH, HBHA(CO)NH, C(CCO)NH, HC(C)H-TOCSY, (H)CCH-TOCSY, HC(C)H-COSY, (HB)CB(CGCD)HD, ^15^N-edited NOESY-HSQC (80 ms mixing time), and ^13^C-edited NOESY-HSQC (80 ms mixing time). The NMR spectra were processed and analyzed as previously described^56^. The 20 structures of the final calculation cycle with the lowest target function values were further refined with the program AMBER^57^. The mean structure of these refined structures was also shortly energy-minimized with AMBER. For the confirmation of φ angle, HNHA experiment was performed with an NMR sample containing 1.0 mM ^15^N labeled proteins^58^. The electrostatic potential was calculated by the program GRASP^59^. Graphic rendering was processed in the program MOLMOL^60^.

### Isothermal Titration Calorimetry (ITC)

Synthesized phosphotyrosyl peptides (95% or greater purity by HPLC) were purchased from Toray Research Center. Based on a pilot experiment with different peptide lengths, we found that 8-mer was the minimal length to obtain reliable ITC and NMR titration results (not shown). Isothermal titration calorimetry (ITC) was performed in MicroCal Auto-iTC200 (Malvern) at 25 °C as described in the manufacturer’s instruction manual. The SH2 domain and peptide were dissolved in 25 mM HEPES, pH7, 100 mM NaCl at a final concentration of 40 μM or 400 μM, respectively. Titration was carried out by injecting the peptide solution into the reaction cell holding the SH2 domain containing solution every 150 seconds. The results were analyzed by the software ORIGIN 7 supplied by the manufacturer.

### Heterologous Expression and Detection of Protein Association

The human Embryonic Kidney 293 cell line (CRL-1573, ATCC) was used to construct a heterologous expression system by plasmid transfection. *M. brevicollis crka1* was subcloned into a 3′ in-frame cloning site of a CMV-driven humanized GFP vector to generate GFP-Mb crka1. GFP-Hs CRK plasmid was constructed by subcloning the cDNA into pEGFP C1 plasmid (Clontech/TAKARA). HA-Mm Bcar1 is a mouse Bcar1 tagged with a hemagglutinin (HA)-epitope at the N-terminus and subcloned into a CMV expression plasmid. A constitutively active mouse Src in which the negative regulatory tyrosine Y529 was replaced with a phenylalanine residue, Src(Y529F), was subcloned into a Pol2 promoter-driven expression plasmid^6^. Forty-eight hours after transfection, cell lysates were prepared and analyzed by 7.5–15% gradient SDS PAGE and Western blots, or were first subjected to immunoprecipitation followed by electrophoresis and Western blots. GFP-tagged proteins were precipitated by agarose beads chemically coupled with single chain anti-GFP V_H_H (GFP-Trap_A, Chromotek). HA-tag, GFP, Src, and Rapgef1 (C3G) were probed in Western blots using anti-HA (sc-805, Santa Cruz), anti-GFP (sc-8334, Santa Cruz), anti-Src (05–184, EMD Millipore), and anti-C3G (sc-15359, Santa Cruz), respectively.

### Accession Numbers

The mRNA (cDNA) sequences of *Monosiga brevicollis crka1* and *crka2, Saccoglossus kowalevskii crka, Branchiostoma floridae crka1* and *crka2*, and *Petromyzon marinus crkl* have been assigned GenBank accession numbers (KT795324-795329, respectively). In addition to these sequences, Supplementary FASTA file includes a full-length *Ciona intestinalis crka* (cima833d10) transcript sequence and the GENSCAN-predicted coding sequence for *Trichoplax adhaerens crka*. The NMR structure data for *Monosiga brevicollis* crka1 SH2 domain have been assigned Protein Data Bank (PDB) ID number 2RVF.

## Additional Information

**How to cite this article**: Shigeno-Nakazawa, Y. *et al*. A pre-metazoan origin of the *CRK* gene family and co-opted signaling network. *Sci. Rep.* **6**, 34349; doi: 10.1038/srep34349 (2016).

## Supplementary Material

Supplementary Information

## Figures and Tables

**Table 1:** TBLASTN Reveals bcar1 Homologs in Basal Metazoans.

Species	Gene or locus /RefSeq/WGS scaffold	E-values from tblastn search with *Hydra* bcar1 domains			
		SH3 Domain	Substrate Domain (YxxP repeats)§	Serine-rich Domain	FAT-like Domain
*Homo sapiens*	*BCAR1*	7.00E-17	NS* (15)	NS	2.00E-16
*Mus musculus*	*Bcar1*	7.00E-16	NS (15)	NS	5.00E-17
*Saccoglossus kowalevskii*	*bcar1-like*/XM_006811703**/ACQM01029203.1	1.00E-14	NS (13)	3.00E-08	1.00E-13
*Nematostella vectensis*	*bcar1*/ABAV01000884.1	3.00E-14	NS (10)	9.00E-22	8.00E-20
*Amphimedon queenslandica*	LOC105313492/XM_011406973/ACUQ01005063.1 (not annotated)	6.00E-04	NS (6)	NS	8.00E-14
*Trichoplax adhaerens*	TRIADRAFT_52500/XM_002108193/ABGP01000099.1 (not annotated)	8.00E-10	NS (6)	NS	NS
*Salpingoeca rosetta*	PTSG_05573/XP_ 004993441§§	NS	NS (10)§§	NS	NS
	PTSG_12435/XP_004993252§§	NS	NS (9)§§	NS	NS
	PTSG_12436/XP_004993253§§	NS	NS (5)§§	NS	NS
*Monosiga brevicollis*		NS	NS	NS	NS
*Capsaspora owczarzaki*		NS***	NS	NS	NS

^§^The number in parentheses indicates the number of YxxP repeats.

^*^NS: Not Significant, with an arbitrary cut off at an E-value of 0.001.

^**^Our EST plasmid sequences do not agree with the provisional model at the 5′ end including the potential translation start. However, the difference does not extend into the SH3 domain.

^***^Although ACFS02000105.1 is identified by tblastn with *Hydra* bcar1 SH3 sequence at 6E-04, it is an NCK1 homolog (XM_004348736.1). Thus it is listed as NS in this table.

^§§^Not identified by tblastn with *Hydra* bcar1. See Supplementary Table S5.

## References

[b1] Feller S. M. Crk family adaptors-signalling complex formation and biological roles. Oncogene 20, 6348–6371 (2001). 11607838 10.1038/sj.onc.1204779

[b2] Guris D. L., Duester G., Papaioannou V. E. & Imamoto A. Dose-dependent interaction of Tbx1 and Crkl and locally aberrant RA signaling in a model of del22q11 syndrome. Dev Cell 10, 81–92 (2006). 16399080 10.1016/j.devcel.2005.12.002

[b3] Racedo S. E. . Mouse and Human CRKL Is Dosage Sensitive for Cardiac Outflow Tract Formation. Am J Hum Genet 96, 235–244 (2015). 25658046 10.1016/j.ajhg.2014.12.025 PMC4320261

[b4] Birge R. B., Kalodimos C., Inagaki F. & Tanaka S. Crk and CrkL adaptor proteins: networks for physiological and pathological signaling. Cell Commun Signal 7, 13 (2009). 19426560 10.1186/1478-811X-7-13 PMC2689226

[b5] Park T. J. & Curran T. Crk and Crk-like play essential overlapping roles downstream of disabled-1 in the Reelin pathway. J Neurosci 28, 13551–13562 (2008). 19074029 10.1523/JNEUROSCI.4323-08.2008 PMC2628718

[b6] Li L., Guris D. L., Okura M. & Imamoto A. Translocation of CrkL to focal adhesions mediates integrin-induced migration downstream of Src family kinases. Mol Cell Biol 23, 2883–2892 (2003). 12665586 10.1128/MCB.23.8.2883-2892.2003 PMC152569

[b7] Moon A. M. . Crkl deficiency disrupts Fgf8 signaling in a mouse model of 22q11 deletion syndromes. Dev Cell 10, 71–80 (2006). 16399079 10.1016/j.devcel.2005.12.003 PMC1780033

[b8] Seo J. H. . A Specific Need for CRKL in p210BCR-ABL-Induced Transformation of Mouse Hematopoietic Progenitors. Cancer Res 70, 7325–7335 (2010). 20807813 10.1158/0008-5472.CAN-10-0607 PMC2940946

[b9] Jankowski W. . Domain organization differences explain Bcr-Abl’s preference for CrkL over CrkII. Nat Chem Biol 8, 590–596 (2012). 22581121 10.1038/nchembio.954 PMC3423979

[b10] Hasegawa H. . DOCK180, a major CRK-binding protein, alters cell morphology upon translocation to the cell membrane. Mol Cell Biol 16, 1770–1776 (1996). 8657152 10.1128/mcb.16.4.1770 PMC231163

[b11] Ballif B. A. . Activation of a Dab1/CrkL/C3G/Rap1 pathway in Reelin-stimulated neurons. Curr Biol 14, 606–610 (2004). 15062102 10.1016/j.cub.2004.03.038

[b12] Chen K. . Interaction between Dab1 and CrkII is promoted by Reelin signaling. J Cell Sci 117, 4527–4536 (2004). 15316068 10.1242/jcs.01320

[b13] Colicelli J. ABL tyrosine kinases: evolution of function, regulation, and specificity. Sci Signal 3, re6 (2010). 20841568 10.1126/scisignal.3139re6 PMC2954126

[b14] Pincus D., Letunic I., Bork P. & Lim W. A. Evolution of the phospho-tyrosine signaling machinery in premetazoan lineages. Proc Natl Acad Sci USA 105, 9680–9684 (2008). 18599463 10.1073/pnas.0803161105 PMC2443182

[b15] Manning G., Young S. L., Miller W. T. & Zhai Y. The protist, Monosiga brevicollis, has a tyrosine kinase signaling network more elaborate and diverse than found in any known metazoan. Proc Natl Acad Sci USA 105, 9674–9679 (2008). 18621719 10.1073/pnas.0801314105 PMC2453073

[b16] Suga H. . The Capsaspora genome reveals a complex unicellular prehistory of animals. Nat Commun 4, 2325 (2013). 23942320 10.1038/ncomms3325 PMC3753549

[b17] Eichinger L. . The genome of the social amoeba Dictyostelium discoideum. Nature 435, 43–57 (2005). 15875012 10.1038/nature03481 PMC1352341

[b18] Verschueren E. . Evolution of the SH3 Domain Specificity Landscape in Yeasts. PLoS One 10, e0129229 (2015). 26068101 10.1371/journal.pone.0129229 PMC4466140

[b19] Klinghoffer R. A., Sachsenmaier C., Cooper J. A. & Soriano P. Src family kinases are required for integrin but not PDGFR signal transduction. EMBO J 18, 2459–2471 (1999). 10228160 10.1093/emboj/18.9.2459 PMC1171328

[b20] Oktay M., Wary K. K., Dans M., Birge R. B. & Giancotti F. G. Integrin-mediated activation of focal adhesion kinase is required for signaling to Jun NH2-terminal kinase and progression through the G1 phase of the cell cycle. J Cell Biol 145, 1461–1469 (1999). 10385525 10.1083/jcb.145.7.1461 PMC2133163

[b21] Moresco E. M., Donaldson S., Williamson A. & Koleske A. J. Integrin-mediated dendrite branch maintenance requires Abelson (Abl) family kinases. J Neurosci 25, 6105–6118 (2005). 15987940 10.1523/JNEUROSCI.1432-05.2005 PMC6725048

[b22] Sebe-Pedros A., Roger A. J., Lang F. B., King N. & Ruiz-Trillo I. Ancient origin of the integrin-mediated adhesion and signaling machinery. Proc Natl Acad Sci USA 107, 10142–10147 (2010). 20479219 10.1073/pnas.1002257107 PMC2890464

[b23] Sakai R. . A novel signaling molecule, p130, forms stable complexes *in vivo* with v-Crk and v-Src in a tyrosine phosphorylation-dependent manner. EMBO J 13, 3748–3756 (1994). 8070403 10.1002/j.1460-2075.1994.tb06684.x PMC395286

[b24] Chodniewicz D. & Klemke R. L. Regulation of integrin-mediated cellular responses through assembly of a CAS/Crk scaffold. Biochim Biophys Acta 1692, 63–76 (2004). 15246680 10.1016/j.bbamcr.2004.03.006

[b25] Shin N. Y. . Subsets of the major tyrosine phosphorylation sites in Crk-associated substrate (CAS) are sufficient to promote cell migration. J Biol Chem 279, 38331–38337 (2004). 15247284 10.1074/jbc.M404675200

[b26] Donato D. M., Ryzhova L. M., Meenderink L. M., Kaverina I. & Hanks S. K. Dynamics and mechanism of p130Cas localization to focal adhesions. J Biol Chem 285, 20769–20779 (2010). 20430882 10.1074/jbc.M109.091207 PMC2898362

[b27] Sawada Y. . Force sensing by mechanical extension of the Src family kinase substrate p130Cas. Cell 127, 1015–1026 (2006). 17129785 10.1016/j.cell.2006.09.044 PMC2746973

[b28] Patwardhan P., Shen Y., Goldberg G. S. & Miller W. T. Individual Cas phosphorylation sites are dispensable for processive phosphorylation by Src and anchorage-independent cell growth. J Biol Chem 281, 20689–20697 (2006). 16707485 10.1074/jbc.M602311200 PMC2441569

[b29] Honda H. . Cardiovascular anomaly, impaired actin bundling and resistance to Src-induced transformation in mice lacking p130Cas. Nat Genet 19, 361–365 (1998). 9697697 10.1038/1246

[b30] Songyang Z. . SH2 domains recognize specific phosphopeptide sequences. Cell 72, 767–778 (1993). 7680959 10.1016/0092-8674(93)90404-e

[b31] King N. . The genome of the choanoflagellate Monosiga brevicollis and the origin of metazoans. Nature 451, 783–788 (2008). 18273011 10.1038/nature06617 PMC2562698

[b32] Donaldson L. W., Gish G., Pawson T., Kay L. E. & Forman-Kay J. D. Structure of a regulatory complex involving the Abl SH3 domain, the Crk SH2 domain, and a Crk-derived phosphopeptide. Proc Natl Acad Sci USA 99, 14053–14058 (2002). 12384576 10.1073/pnas.212518799 PMC137835

[b33] Kobashigawa Y. . Structural basis for the transforming activity of human cancer-related signaling adaptor protein CRK. Nat Struct Mol Biol 14, 503–510 (2007). 17515907 10.1038/nsmb1241

[b34] Hallock P. T. . Dok-7 regulates neuromuscular synapse formation by recruiting Crk and Crk-L. Genes Dev 24, 2451–2461 (2010). 21041412 10.1101/gad.1977710 PMC2964755

[b35] Hock B. . Tyrosine-614, the major autophosphorylation site of the receptor tyrosine kinase HEK2, functions as multi-docking site for SH2-domain mediated interactions. Oncogene 17, 255–260 (1998). 9674711 10.1038/sj.onc.1201907

[b36] Liu B. A. . SH2 domains recognize contextual peptide sequence information to determine selectivity. Mol Cell Proteomics 9, 2391–2404 (2010). 20627867 10.1074/mcp.M110.001586 PMC2984226

[b37] Schaller M. D. & Parsons J. T. pp125FAK-dependent tyrosine phosphorylation of paxillin creates a high-affinity binding site for Crk. Mol Cell Biol 15, 2635–2645 (1995). 7537852 10.1128/mcb.15.5.2635 PMC230493

[b38] Matsumoto T. . Differential interaction of CrkII adaptor protein with platelet-derived growth factor alpha- and beta-receptors is determined by its internal tyrosine phosphorylation. Biochem Biophys Res Commun 270, 28–33 (2000). 10733900 10.1006/bbrc.2000.2374

[b39] Salameh A., Galvagni F., Bardelli M., Bussolino F. & Oliviero S. Direct recruitment of CRK and GRB2 to VEGFR-3 induces proliferation, migration, and survival of endothelial cells through the activation of ERK, AKT, and JNK pathways. Blood 106, 3423–3431 (2005). 16076871 10.1182/blood-2005-04-1388

[b40] Eddy S. R. Accelerated Profile HMM Searches. PLoS Comput Biol 7, e1002195 (2011). 22039361 10.1371/journal.pcbi.1002195 PMC3197634

[b41] Ohno S. Evolution by Gene Duplication (Springer Science and Business Media, New York, 1970).

[b42] Kuraku S. Insights into cyclostome phylogenomics: pre-2R or post-2R. Zoolog Sci 25, 960–968 (2008). 19267631 10.2108/zsj.25.960

[b43] Smith J. J. . Sequencing of the sea lamprey (Petromyzon marinus) genome provides insights into vertebrate evolution. Nat Genet 45, 415–21, 421e1 (2013). 23435085 10.1038/ng.2568 PMC3709584

[b44] Singh P. P., Arora J. & Isambert H. Identification of Ohnolog Genes Originating from Whole Genome Duplication in Early Vertebrates, Based on Synteny Comparison across Multiple Genomes. PLoS Comput Biol 11, e1004394 (2015). 26181593 10.1371/journal.pcbi.1004394 PMC4504502

[b45] Dehal P. & Boore J. L. Two rounds of whole genome duplication in the ancestral vertebrate. PLoS Biol 3, e314 (2005). 16128622 10.1371/journal.pbio.0030314 PMC1197285

[b46] Deakin N. O. & Turner C. E. Paxillin comes of age. J Cell Sci 121, 2435–2444 (2008). 18650496 10.1242/jcs.018044 PMC2522309

[b47] Hagel M. . The adaptor protein paxillin is essential for normal development in the mouse and is a critical transducer of fibronectin signaling. Mol Cell Biol 22, 901–915 (2002). 11784865 10.1128/MCB.22.3.901-915.2002 PMC133539

[b48] Srivastava M. . The Trichoplax genome and the nature of placozoans. Nature 454, 955–960 (2008). 18719581 10.1038/nature07191

[b49] Sela I., Ashkenazy H., Katoh K. & Pupko T. GUIDANCE2: accurate detection of unreliable alignment regions accounting for the uncertainty of multiple parameters. Nucleic Acids Res 43, W7–14 (2015). 25883146 10.1093/nar/gkv318 PMC4489236

[b50] Katoh K. & Standley D. M. MAFFT multiple sequence alignment software version 7: improvements in performance and usability. Mol Biol Evol 30, 772–780 (2013). 23329690 10.1093/molbev/mst010 PMC3603318

[b51] Silvestro D. & Michalak I. RaxmlGUI: a graphical front-end for RAxML. Organisms Diversity & Evolution 12, 335–337 (2012).

[b52] Stamatakis A. RAxML version 8: a tool for phylogenetic analysis and post-analysis of large phylogenies. Bioinformatics 30, 1312–1313 (2014). 24451623 10.1093/bioinformatics/btu033 PMC3998144

[b53] Ronquist F. . MrBayes 3.2: efficient Bayesian phylogenetic inference and model choice across a large model space. Syst Biol 61, 539–542 (2012). 22357727 10.1093/sysbio/sys029 PMC3329765

[b54] Yabuki T. . A robust two-step PCR method of template DNA production for high-throughput cell-free protein synthesis. J Struct Funct Genomics 8, 173–191 (2007). 18167031 10.1007/s10969-007-9038-z

[b55] Matsuda T. . Improving cell-free protein synthesis for stable-isotope labeling. J Biomol NMR 37, 225–229 (2007). 17237976 10.1007/s10858-006-9127-5

[b56] Li H. . Structure of the C-terminal phosphotyrosine interaction domain of Fe65L1 complexed with the cytoplasmic tail of amyloid precursor protein reveals a novel peptide binding mode. J Biol Chem 283, 27165–27178 (2008). 18650440 10.1074/jbc.M803892200

[b57] Case D. A. . The Amber biomolecular simulation programs. J Comput Chem 26, 1668–1688 (2005). 16200636 10.1002/jcc.20290 PMC1989667

[b58] Bax A. . Measurement of homo- and heteronuclear J couplings from quantitative J correlation. Methods Enzymol 239, 79–105 (1994). 7830604 10.1016/s0076-6879(94)39004-5

[b59] Nicholls A., Sharp K. A. & Honig B. Protein folding and association: insights from the interfacial and thermodynamic properties of hydrocarbons. Proteins 11, 281–296 (1991). 1758883 10.1002/prot.340110407

[b60] Koradi R., Billeter M. & Wüthrich K. MOLMOL: a program for display and analysis of macromolecular structures. J Mol Graph 14, 51–5, 29 (1996). 8744573 10.1016/0263-7855(96)00009-4

