# Supplementary material for: A pre-metazoan origin of the CRK gene family and co-opted signaling network
Source: Sci Rep. 2016 Sep 30;6:34349. doi: 10.1038/srep34349 (PMC5043372; doi:10.1038/srep34349)
Supplement: Supplementary Information [file srep34349-s1.pdf]

SUPPLEMENTARY INFORMATION

MANUSCRIPT TITLE:

A pre-metazoan origin of the *CRK* gene family and co-opted signaling network

AUTHORS:

Yoko Shigeno-Nakazawa, Takuma Kasai, Ki Sewon, Elina Kostyanovskaya, Jana Pawlak, Junya Yamagishi, Noriaki Okimoto, Makoto Taiji, Mariko Okada, Jody Westbrook, Yoko Satta, Takanori Kigawa, and Akira Imamoto

This Supplementary Information file includes:

8 tables (Supplementary Tables 1-8)

10 figures (Supplementary Figures 1-10)

1 data file (Supplementary Data 1)

Supplementary Table S1. The source sequences used for phylogenetic analysis

			NCBI RefSeq (reviewed)	NCBI RefSeq (provisional/predicted)	Ensembl transcript	Other transcriptome database	Full-length EST sequenced	Full length RT-PCR product cloned and sequenced	Predicted from genomic sequences	Chromosomal Location or Scaffold
Metazoa	Bilateria>Chordata>Vertebrata	**H_sapiens_CRK**	NM_016823.3							17p13
		**H_sapiens_CRKL**	NM_005207.3							22q11
		**M_musculus_Crk**	NM_133656.5							Ch 11
		**M_musculus_Crkl**	NM_007764.5							Ch 16
		**G_gallus_CRK**		NM_001007846.1						Ch 19
		**G_gallus_CRKL*** **		XM_415233.4						Ch 15
		**X_tropicalis_crk**		NM_001006107.1						NW_004668233
		**X_tropicalis_crkl**		!!! (NM_203729.1)	ENSXETT00000009283					NW_004668232 (!!!)
		**D_rerio_crk**		NM_001003628.2						Ch 15
		**D_rerio_crkl**		NM_213538.1						Ch 21
		**L_erinacea_crk**				SkateBase (LSb2: LittleSkate_TranscriptomeContig10952), not annotated				LSb2-ctg36384 LittleSkate_ConsensusfromContig36384; LSb2-ctg24003 LittleSkate_ConsensusfromContig24003; LSb2-ctg2662823 LittleSkate_ConsensusfromContig2662823
		**L_erinacea_crkl**				SkateBase (LSb2: LittleSkate_TranscriptomeContig10415), not annotated				LSb2-ctg50573 LittleSkate_ConsensusfromContig50573; LSb2-ctg22367 LittleSkate_ConsensusfromContig22367; LSb2-ctg1642667 LittleSkate_ConsensusfromContig1642667
		**P_marinus_crkl***			!! (ENSPMAT00000009218)			clone 10 and three other nearly identical clones 3, 20, 31		Pmarinus_7.0_GL478324
	Bilateria>Chordata (except for vertebrata)	**C_intestinalis_crka***		XM_002130671.2			cima833d10 (KH.C12.637.v1.A.SL1-1)			Ch 12 : scaffold_140_Mar. 2005, JGI 2.1/ci2
		**B_floridae_crka1***					bflv061c08			Bf_v1.0_scaffold_202; Bf_v1.0_scaffold_18; Bf_V2_70
		**B_floridae_crka2***					bflv57k18			Bf_v1.0_scaffold_131; Bf_V2_2
		**S_purpuratus_crka*** **		XM_777316.4 (annotated as crkl; longest of the three variants)						NW_011995600.1 (LOC577062)
		**S_kowalevskii_crka***		!! (XM_006822248; annotated as *crk-like protein like* ; missing an exon sequence due to a stretch of genomic sequence ambiguity)			G613P6149RE5.T0			NW_003143829.1
	Cnidaria	**H_vulgaris_crka1*** **		XM_012705689.1/ XM_012705690.1 (annotated as crkl)						GAOL01023343, NW_004170364.1 (LOC100206935)
		**H_vulgaris_crka2*** **		XM_002164309.3 (annotated as crkl)						NW_004166903.1 (LOC100205773)
		**C_hemisphaerica_crka****				Compagen CHEM_T-CDS (unigene013260)				
		**N_vectensis_crka**		XM_001630513		StellaBase Nv.T1 Reference Transcriptome (Nv.T1.20878.1; Nv.T1.3880.2) (annotated as crkl)				Nemve1_scaffold_119, NEMVEDRAFT_v1g233027
	Sponge	**A_queenslandica_crka*** **		XM_003384170.2 (annotated as crkl)						NW_003546265.1
	Placozoa	**T_adhaerens_crka*** **							GENSCAN predicted	ABGP01000065
Filozoa (except for metazoa)	Choanoflagellate	**M_brevicollis_crka1***		!! (XM_001745882.1)				Three clones 2.21, 2.23, and 2.24		scaffold_10|1|1258938, MONBRDRAFT_25438
		**M_brevicollis_crka2***		!! (XM_001745746.1)				Three clones 2.25, 2.26, and 2.27		scaffold_10|1|1258938, MONBRDRAFT_25437
		**S_rosetta_crka*** **		XM_004993927.1 (annotated as crkl)						NW_004754922.1 > PTSG_05410
	Filasterea	**C_owczarzaki_crka*** **		XM_004364986.2 (annotated as crkl)						ACFS02000011

*Sequences based on full-length ESTs, or transcripts isolated, or predicted from WGS (this report)

**Sequences based on transcriptome database

***Provisional/Model sequences based only on WGS sequences

!! RefSeq model/provisional sequences (shaded blue) differ from cDNA clones or full length ESTs obtained from mRNA in the lab or transcriptome

!!! RefSeq model is not predicted correctly from the genomic scaffold for exon 3. Ensemble transcript is more consistent with exon-intron boundaries.

Supplementary FASTA file includes the sequences in the boxes shaded pink

Table S2 Restraints and statistics of the structure calculation of *Monosiga* crka1 SH2

**NOE upper distance limits**	
Intraresidual ($\|i-j\|=0$)	389
Sequential ($\|i-j\|=1$)	441
Medium range ($1 < \|i-j\| < 5$)	246
Long range ($\|i-j\| \geq 5$)	735
Torsion angle restraints	104
CYANA target function value	0.0804 ± 0.0013
Distance restraint violations (>0.15 Å)	0
Torsion angle restraint violations (>5°)	1
AMBER energy values (kcal/mol)	-3755.0 ± 8.9
**rms deviation of coordinates**[a]	
Backbone atoms (Å)	0.301 ± 0.055
Non-hydrogen atoms (Å)	0.618 ± 0.054
**Ramachandran plot statistics**[a,b]	
Residues in favored regions	88.2%
Residues in additionally allowed regions	10.4%
Residues in generously allowed regions	0.1%
Residues in disallowed regions	1.3%

[a] for residues W8-L95

[b] by the program PROCHECK

Table S3 Characteristics of i+1 residues of β turns of EF loop and standard β turns.

		ϕ angle [degree]	calculated $^{3}J_{HNHA}$ [Hz] [b]	experimental $^{3}J_{HNHA}$ [Hz]
β turns of Crk SH2 structures	Mb-Crka1 (this work)	53 ± 2	6.27	6.67
	Hs-CRKL (2EO3)	75 ± 3	5.97	
	Hs-CRK (2EYV)	54	6.29	
standard β turns [a]	type I	-64	4.62	
	type II	-60	4.11	
	type VIII	-72	5.69	
	type I’	55	6.31	
	type II’	60	6.35	
	type VIa1	-64	4.62	
	type VIa2	-132	9.55	
	type VIb	-135	9.37	

[a] values of ϕ angles are taken from Hutchinson *et al*. [1]

[b] $^{3}J_{HNHA}$ values are calculated from ϕ angles by Karplus equation according to Vuister *et al*. [2]

1. Hutchinson EG, Thornton JM. A revised set of potentials for beta-turn formation in proteins. Protein Sci. 1994;3: 2207-2216
2. Vuister GW, Bax A. Quantitative *J* correlation: a new approach for measuring homonuclear three-bond *J*($H^{N}H^{\alpha}$) coupling constants in ^{15}N-enriched proteins. J Am Chem Soc. 1993;115: 7772-7777

Table S4. Domain Similarites between BCAR1 Orthologs

	SH3 Domain (57/57)*	Substrate Domain (76/560)**	Ser-rich Domain (137/179)	FAT-like Domain (114/118)
H_sapiens_BCAR1	100	100	100	100
M_musculus_Bcar1	98	95	87	98
R_norvegicus_Bcar1	98	96	89	97
X_tropicalis_bcar1	95	63	55	80
C_intestinalis_cima813i05	70	57	31	39
B_floridae_XP_002605932	82	47	43	61
S_kowalevskii_bcar1 (contig 189)	79	51	36	57
Hydra_bcar1_XP_002164065	77	25	18	34
A_queenslandica_XP_011405275	49	42	14	37
Trichoplax_bcar1	61	32	15	21

The numbers in the table columns are % similarity values from pairwise comparisons with Human BCAR1 domains after gaps are removed in an MAFFT protein alignment.

*The numbers in parentheses indicate the aligned amino acid length after versus before gap removal.

** The substrate domain is a poorly aligned region containing many gaps. See also Table 2. The % similarity values may be overestimated after removal of the gaps.

Table S5. BLASTp with SH2 binding short peptide sequences over RefSeq protein databases

	Species	Proteins	Subject Sequences	E-value	Hits per protein	
BLASTp with DEYDTPRH						
	Human	BCAR1	DEYDIPRH; YDTP; YDVPR	0.32	multiple	
		P2RX4	EYDTPR	3.7	single	
		P2RX1	EYDTPR	3.8	single	
		CARS2	DDFDTPR	24	single	No tyrosine
		HNF1B	DDYDTP	24	single	
		PCDHA7	DETDAPRH	33	single	No tyrosine
		PCDHA4	DETDAPRH	33	single	No tyrosine
		DOK7	YDTPR	43	single	
	Mouse	Bcar1	DEYDTPRH; YDTP; YDVPR	0.015	multiple	
		P2rx4	EYDTPR	3	single	
		P2rx1	EYDTPR	3	single	
		Hnf1b	DDYDTP	19	single	
		Setd3	EYDTP	35	single	
		Il5ra	DEYDT	35	single	
		Dok7	YDTPR	35	single	
		Shisa7	YDTPR	35	single	
	Salpingoeca	PTSG_12436	YDTPRH; EYDLPR; YDNPR; YDNPR	0.44	multiple	
		PTSG_12435	DEYDYPR; DEQQYDVPR; EYAVPR; YDLPR	2.7	multiple	
		PTSG_01567	YDSPRH	3.7	single	
		dynein	DDYDTP	3.8	single	
		PTSG_06396	DEYDT	6.6	single	
		PTSG_01720	YDTPR	6.7	single	
		PTSG_05573	YDTPR; YDTPR; DMYDVPR; DMYDVPR; Y[illegible]	6.8	multiple	
		notch2	YATPRH	13	single	
BLASTp with HHYDTPRS						
	Salpingoeca	PTSG_08164	HHYETPRS	0.027	single	
		PTSG_04726	HHYDDTPR	0.6	single	
		PTSG_05573	YDTPRS; YDTPR; YDVPRS; YDVPRS; YDVP	1.1	multiple	
		PTSG_08942	HHYDT	3.7	single	
		sry	HYDTP;	5.1	single	
		PTSG_09521	HHQQYDSPR	6.7	single	
		PTSG_01720	YDTPR	6.7	single	
		PTSG_12056	YDTPR	6.8	single	
		PTSG_11582	HYDCPRS	6.8	single	
		PTSG_12436	YDTPR; YDNPR; YDNPR; YDLPR	6.8	multiple	
		PTSG_12435	HHYEQP; QQYDVPR; YDYPR; YDLPR	82	multiple	

Supplementary Table S6 Profile HMM Search Results

	UniProt Accession	YxxP*	InterPro**	Pfam**	Smart**	E-value
S. rosetta						
	F2UCP6	5	IPR000008. C2_dom. ; IPR001881. EGF-like_Ca-bd_dom. ; IPR013032. EGF-like_CS. ; IPR000742. EGF-like_dom.		SM00181. EGF. 9 hits. ; SM00179. EGF_CA. 6 hits.	3.8E-251
	F2UBL2	10	IPR032179. DUF5011. ; IPR000980. SH2.	PF16403. DUF5011. 1 hit.		1.7E-208
	F2UCP5	9	IPR000008. C2 dom.	PF00168. C2. 1 hit.		1.7E-182
	F2UBL6	4	IPR032179. DUF5011. ; IPR009030. Growth_fac_rcpt_. ; IPR000980. SH2. ; IPR001368. TNFR/NGFR_Cys_rich_reg. ; IPR011641. Tyr-kin_ephrin_A/B_rcpt-like.	PF16403. DUF5011. 2 hits. ; PF00017. SH2. 1 hit. ; PF00020. TNFR_c6. 1 hit.	SM01411. Ephrin_rec_like. 8 hits. ; SM00208. TNFR. 10 hits.	9E-74
	F2UKG9	4	IPR000008. C2_dom. ; IPR001881. EGF-like_Ca-bd_dom. ; IPR013032. EGF-like_CS. ; IPR000742. EGF-like_dom.	PF00168. C2. 1 hit.	SM00181. EGF. 9 hits. ; SM00179. EGF_CA. 6 hits.	6.2E-70
	F2UH22	5	IPR001881. EGF-like_Ca-bd_dom. ; IPR013032. EGF-like_CS. ; IPR000742. EGF-like_dom.		SM00181. EGF. 9 hits. ; SM00179. EGF_CA. 4 hits.	3.8E-64
	F2U6S2	4	IPR000008. C2_dom. ; IPR013032. EGF-like_CS. ; IPR000742. EGF-like_dom.		SM00181. EGF. 10 hits.	4.6E-19
	F2UDW2	4	IPR032179. DUF5011. ; IPR006212. Furin_repeat. ; IPR009030. Growth_fac_rcpt_. ; IPR000980. SH2. ; IPR001368. TNFR/NGFR_Cys_rich_reg. ; IPR011641. Tyr-kin_ephrin_A/B_rcpt-like.	PF16403. DUF5011. 5 hits. ; PF07699. Ephrin_rec_like. 6 hits. ; PF00017. SH2. 2 hits. ; PF00020. TNFR_c6. 10 hits.	SM01411. Ephrin_rec_like. 13 hits. ; SM00261. FU. 6 hits. ; SM00252. SH2. 2 hits. ; SM00208. TNFR. 31 hits.	1.5E-15
	F2UMJ5	4	IPR032179. DUF5011. ; IPR000980. SH2.	PF16403. DUF5011. 1 hit. ; PF00017. SH2. 1 hit.	SM00252. SH2. 1 hit.	1.2E-09
M. brevicollis						
	A9V449	5	IPR032179. DUF5011. ; IPR009030. Growth_fac_rcpt_. ; IPR000980. SH2.	PF16403. DUF5011. 1 hit. ; PF00017. SH2. 1 hit.	SM00252. SH2.	8.2E-303
	A9VAL3	11	IPR000980. SH2. ; IPR000884. TSP1_rpt.	PF00017. SH2. 1 hit.	SM00209. TSP1. 3 hits.	6.7E-217
	A9URG3	4	IPR032179. DUF5011. ; IPR009030. Growth_fac_rcpt_. ; IPR003410. HYR_dom. ; IPR003347. JmjC_dom. ; IPR000980. SH2. ; IPR001368. TNFR/NGFR_Cys_rich_reg. ; IPR011641. Tyr-kin_ephrin_A/B_rcpt-like.	PF08007. Cupin_4. 1 hit. ; PF16403. DUF5011. 1 hit. ; PF07699. Ephrin_rec_like. 1 hit. ; PF02494. HYR. 1 hit. ; PF00017. SH2. 1 hit.	SM01411. Ephrin_rec_like. 6 hits. ; SM00252. SH2. 1 hit. ; SM00208. TNFR. 4 hits.	3.1E-25
	A9V5T5	10	IPR005579. Cgr1-like. ; IPR011010. DNA_brk_join_enz. ; IPR013034. DNA_topo_domain1. ; IPR032179. DUF5011. ; IPR000980. SH2. ; IPR001368. TNFR/NGFR_Cys_rich_reg. ; IPR001631. TopoI. ; IPR018521. TopoI_AS. ; IPR025834. TopoI_C_dom. ; IPR014711. TopoI_cat_a-hlx-sub_euk. ; IPR014727. TopoI_cat_a/b-sub_euk. ; IPR013500. TopoI_cat_euk. ; IPR008336. TopoI_DNA-bd_euk. ; IPR013030. TopoI_DNA-bd_mixed-a/b_euk. ; IPR013499. TopoI_euk. ; IPR011641. Tyr-kin_ephrin_A/B_rcpt-like.	PF03879. Cgr1. 1 hit. ; PF16403. DUF5011. 1 hit. ; PF00017. SH2. 2 hits. ; PF14370. Topo_C_assoc. 1 hit. ; PF01028. Topoisom_I. 1 hit. ; PF02919. Topoisom_I_N. 1 hit.	SM01411. Ephrin_rec_like. 2 hits. ; SM00252. SH2. 2 hits. ; SM00208. TNFR. 2 hits. ; SM00435. TOPEUc. 1 hit.	5E-21
	A9V0E4	14	IPR009030. Growth_fac_rcpt_. ; IPR003347. JmjC_dom. ; IPR000980. SH2. ; IPR001368. TNFR/NGFR_Cys_rich_reg. ; IPR011641. Tyr-kin_ephrin_A/B_rcpt-like.	PF08007. Cupin_4. 1 hit. ; PF00017. SH2. 2 hits.	SM01411. Ephrin_rec_like. 5 hits. ; SM00252. SH2. 2 hits. ; SM00208. TNFR. 3 hits.	1.6E-19
	A9VDD4	15	IPR032179. DUF5011. ; IPR006212. Furin_repeat. ; IPR009030. Growth_fac_rcpt_. ; IPR003347. JmjC_dom. ; IPR000980. SH2. ; IPR011641. Tyr-kin_ephrin_A/B_rcpt-like.	PF08007. Cupin_4. 1 hit. ; PF16403. DUF5011. 2 hits. ; PF07699. Ephrin_rec_like. 1 hit. ; PF00017. SH2. 2 hits.	SM01411. Ephrin_rec_like. 4 hits. ; SM00261. FU. 3 hits. ; SM00252. SH2. 2 hits.	9.5E-13
	A9V9N2	4	IPR009045. Hedgehog_sig/DD-Pept_Zn-bd_dom. ; IPR000980. SH2. ; IPR002035. VWF_A.	PF00017. SH2. 1 hit. ; PF00092. VWA. 1 hit.	SM00252. SH2. 1 hit. ; SM00327. VWA. 1 hit.	0.0000015
C. owczarzaki						
	A0A0D2WNQ3	4	IPR019931. LPXTG_anchor. ; IPR006626. PbH1. ; IPR012334. Pectin_lyas_fold. ; IPR011050. Pectin_lyase_fold/virulence.		SM00710. PbH1. 6 hits.	0.0000053
	A0A0D2WIP2	4	IPR009030. Growth_fac_rcpt_. ; IPR011009. Kinase-like_dom. ; IPR032675. L_dom-like. ; IPR001611. Leu-rich_rpt. ; IPR003591. Leu-rich_rpt_typical-subtyp. ; IPR026906. LRR_5. ; IPR000719. Prot_kinase_dom. ; IPR001245. Ser-Thr/Tyr_kinase_cat_dom. ; IPR008266. Tyr_kinase_AS. ; IPR020635. Tyr_kinase_cat_dom.	PF13306. LRR_5. 1 hit. ; PF13855. LRR_8. 2 hits.	SM00369. LRR_TYP. 10 hits. ; SM00219. TyrKc. 1 hit.	0.0009

A profile hidden Markov model was constructed from an MAFFT (E-INS-i) alignment of the five proteins in the yellow shade, and was used to search related proteins in UniProt databases as described in Methods. As expected, these source proteins returned best E-values in the searches. This table shows only proteins that have 4 or more YxxP motifs. F2BL6, F2CP5, and F2CP6 are UniProt Accession numbers for PTSG_05573, 12435, and 12436, respectively.

* This column indicates the number of YxxP motifs in the predicted protein.

** InterPro, Pfam, and Smart are domain family databases. The features shown are from the protein-specific page of UniProtKB (UniProt Knowledge Base) .

Table S7. Partial synteny between Ciona Ch12 and vertebrate chromosomes

**C_intestinalis (at UCSC > JGI_2.1/ci2 or GhostDatabase-Kyoto) scaffold 140 / KhC12 = chr12**	**X. tropicalis (Ensembl > JGI_4.2); scaffolds GL173559, GL172708, and GL172648**	**P. marinus (Ensembl > Pmarinus_7.0)**	**Human**	**Hs other paralogs**
**crka (scaffold_140)**	**crk (GL173559); crkl (GL172648)**	**crkl (GL478324)**	**CRK (17p13.3); CRKL (22q11.21)**	none
No klhl22, but another Kelch-like, **klhl12**, is on KhC12; many others are on KhL29 KhC5; KhL87; etc	**klhl22 (GL172648)**	**klhl22 (GL478324)**	**KLHL10 (17q21.2); KLHL22 (22q11.21)**	KLHL12 (1); KLHL17 (1); KLHL20 (1); KLHL18 (3); KLHL5 (4); KLHL8 (4); KLHL2 (4); KLHL3 (5); KLHL1 (13); KLHL28 (14); KEAP1 (19); KLHL4 (X);
**snap29* (scaffold_140)**	**snap29 (GL172648)**		**SNAP29 (22q11.21)**	none
No T-box genes in Ch12; tbx1 (chr07q:2,753,474-2,759,703; KhC7); tbx2/3 (chr04q:5,688,919-5,703,952; KhL8); tbx4 (KhC3)	**tbx2 (GL172708); tbx4 (GL172708); tbx1 (GL172648)**		**TBX2 (17q23.2); TBX4 (17q23.2); TBX1 (22q11.21)**	TBX15 (1); TBX19 (1); T (6); TBX18 (6); TBX20 (7); TBX10 (11), TBX3 (12); TBX5 (12), TBX6 (16); TBX22 (X);
**aldh3a1/2 (scaffold_140)**	**aldh3a2 (GL172708)**		**ALDH3A1 (17p11.2); ALDH3A2 (17p11.2)**	ALDH3B1 (11); ALDH3B2 (11)
**cltc/cltcl1 (scaffold_140)**	**cltc (GL172708); cltcl1 (GL172648)**		**CLTC (17q23.1); CLTCL1 (22q11.21)**	none

Blue indicates gene and scaffold in parentheses syntenic to human chromosome 17.

Magenta indicates gene and scaffold in parentheses syntenic to human chromosome 22.

Red indicates gene and scaffold in parentheses syntenic to human chromosomes 17 and 22.

*Partial sequence.

Table S8 Networking with possible CRK/CRKL binding proteins

CRK/CRKL binding proteins			Orthologs of CRK/CRKL binding proteins			
			Premetazoans		Basal Metazoans	
via SH2	via SH3n	Function	Capsaspora	Choanoflagellates	Sponge	Hydra vulgaris
BCAR1 ($p130^{CAS}$)		scaffold	N	N	Y (+ YxxP sites)	Y (+ YxxP sites)
PXN		scaffold	Y (no YxxP sites)	Y (no conserved YxxP sites)*	Y (no conserved YxxP sites)	Y (no conserved YxxP sites)
Dok7 (and other Dok)		scaffold	N	N	N	N
GAB1 (and GAB2)		scaffold	N	N	Y (+ YxxP sites)	N
IRS1 (and IRS2)		scaffold	N	N	N	Y (no YxxP)
PDGFRA (and PDGFRB)		receptor tyrosine kinase	N	N	N	Y (no YxxP)
	ABL (ABL1 and ABL2)	tyrosine kinase	Y (+ PxxP motifs)#	Y (no proline-rich region)	Y (+ PxxPxK motifs)	Y (+ PxxPxK motifs)
	C3G (RAPGEF1)	guanine nucleotide exhange for RAP	N	N	N	Y (+ PxxPxK motifs)
	DOCK1 (DOCK180)	guanine nucleotide exhange for RAC	Y (+ PxxPxK motifs)§	Y (+ PxxPxK motifs)§	Y (+ PxxPxK motifs)	Y (+ PxxPxK motifs)
	SOS (SOS1 and other SOS)	guanine nucleotide exhange for RAS	Y (+ PxxPxK motifs)§	Y (no PxxPxK motifs)	Y (+ PxxPxK motifs)	Y (+ PxxPxK motifs)

Y or N indicates the presence or absence of orthologs, respectively.

* *Monosiga brevicollis* is predicted to have a protein related to paxillin. However, it has only LIM domains, lacking a region that corresponds to the N-terminal half of mammalian paxillin. *Salpingoeca rosetta* also has a paxillin-related protein which includes a few YxxP motifs. Although they may serve as SH2 binding sites if phosphorylated, these sites do not correspond to Y31 or Y118 of mammalian paxillin.

# Although PxxPxK motifs do not exist, the ABL ortholog has a few divergent PxxP motifs that may serve as SH3 binding sites (but lacking a C-terminal F-actin binding domain).

§ Although PxxPxK motifs exist in the C-terminal region of the premetazoan ortholog, the region is highly divergent.

Supplementary Figure S1 *CRK/CRKL* orthologs in *Monosiga brevicollis*

(a and b) Pairwise nucleotide sequence alignment of *Monosiga crka1* or *crka2* with the corresponding model sequences XM_001745882 or XM_001745746, respectively. The possible coding sequences are highlighted in yellow. The regions that correspond to SH2 and SH3 domains are highlighted in pink. The identical bases are in black. Note that the model sequences XM_001745882 and XM_001745746 lack 5’ sequences in the region that corresponds to the start of the SH2 domain.

Supplementary Figure S1

a

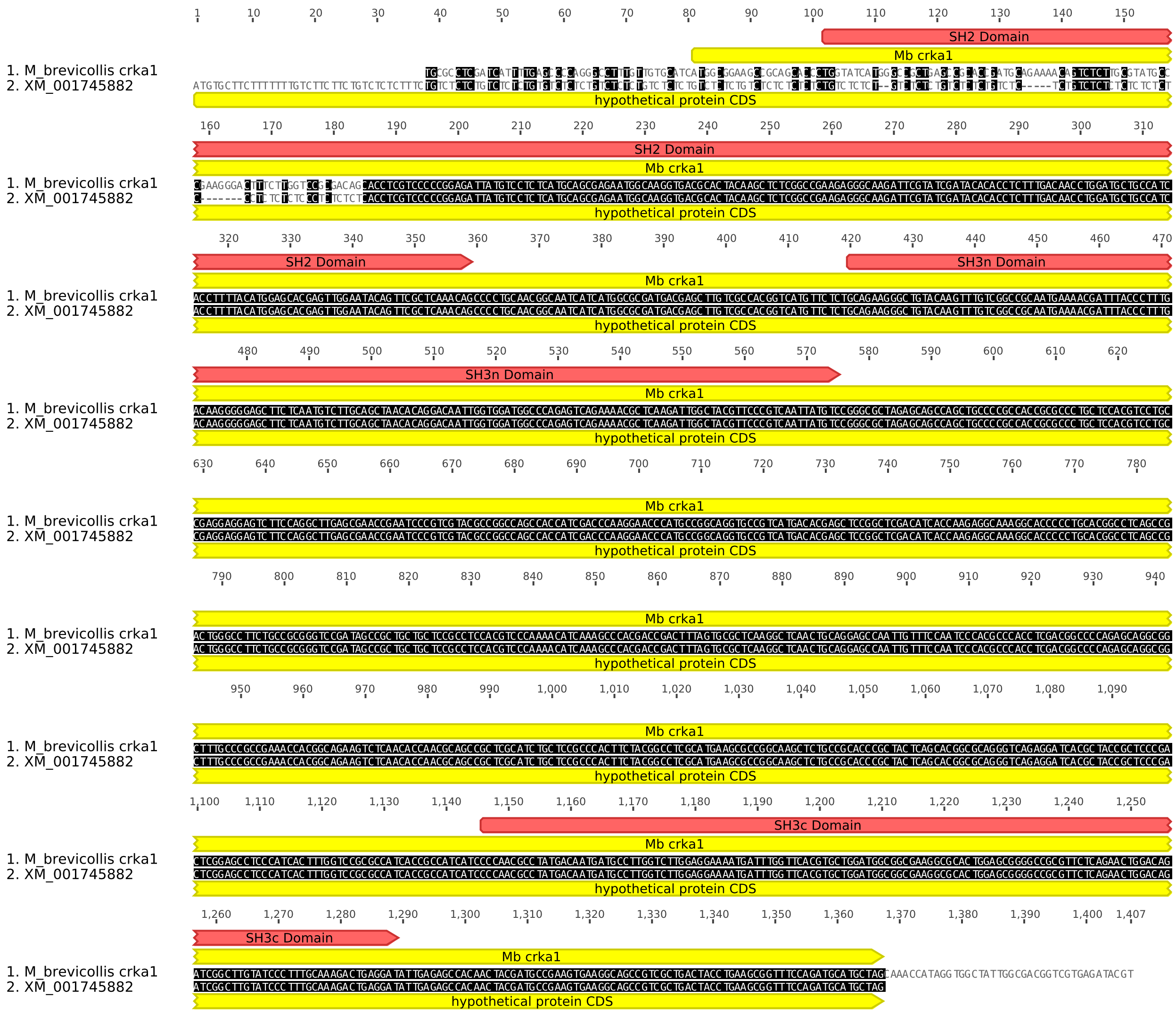

Supplementary Figure S1

b

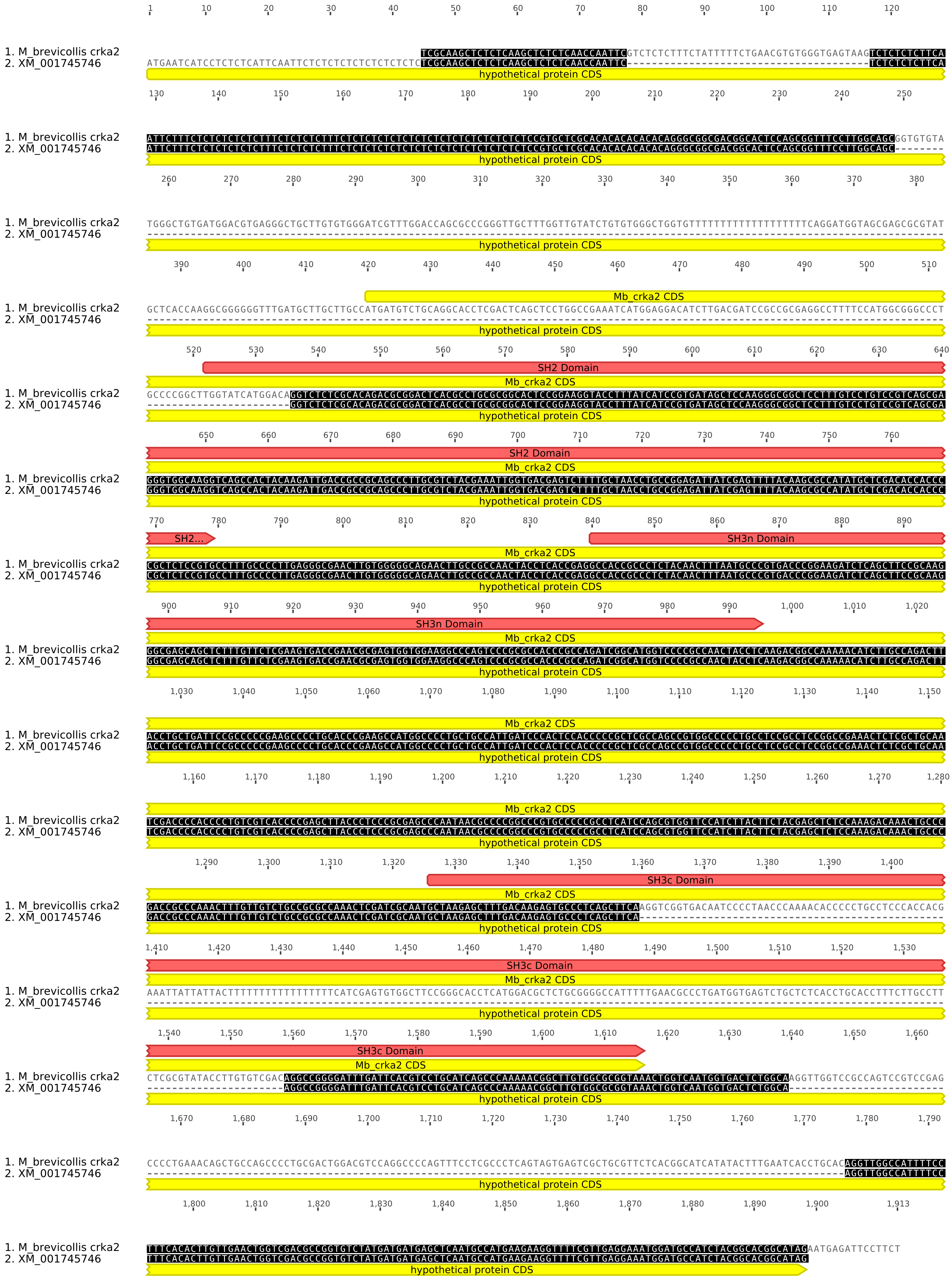

Supplementary Figure S2 *crkl* in *Petromyzon marinus*

(a) A diagram displays the *crkl* locus in *P. marinus*. The coding sequences (yellow) span over two exons (gray boxes).

(b) Shown is a nucleotide alignment of the model sequence posted at ENSEMBL and two *crkl* transcripts (clones 10 and 20) out of a total of four clones obtained. Only coding sequences are shown. % identity is indicated by green, yellow, or no shades corresponding to 100%, between 60-80%, or below 60%, respectively. Note that while some polymorphisms are identified among the expressed clones, the ENSEMBL model sequence lacks a stretch of 90 bases that exist in the transcribed sequences between the SH2 and SH3n domains (the consensus nucleotides 300 to 389, highlighted yellow). Pink bars below the consensus indicate the regions corresponding to the SH2 and SH3 domains.

Supplementary Figure S2

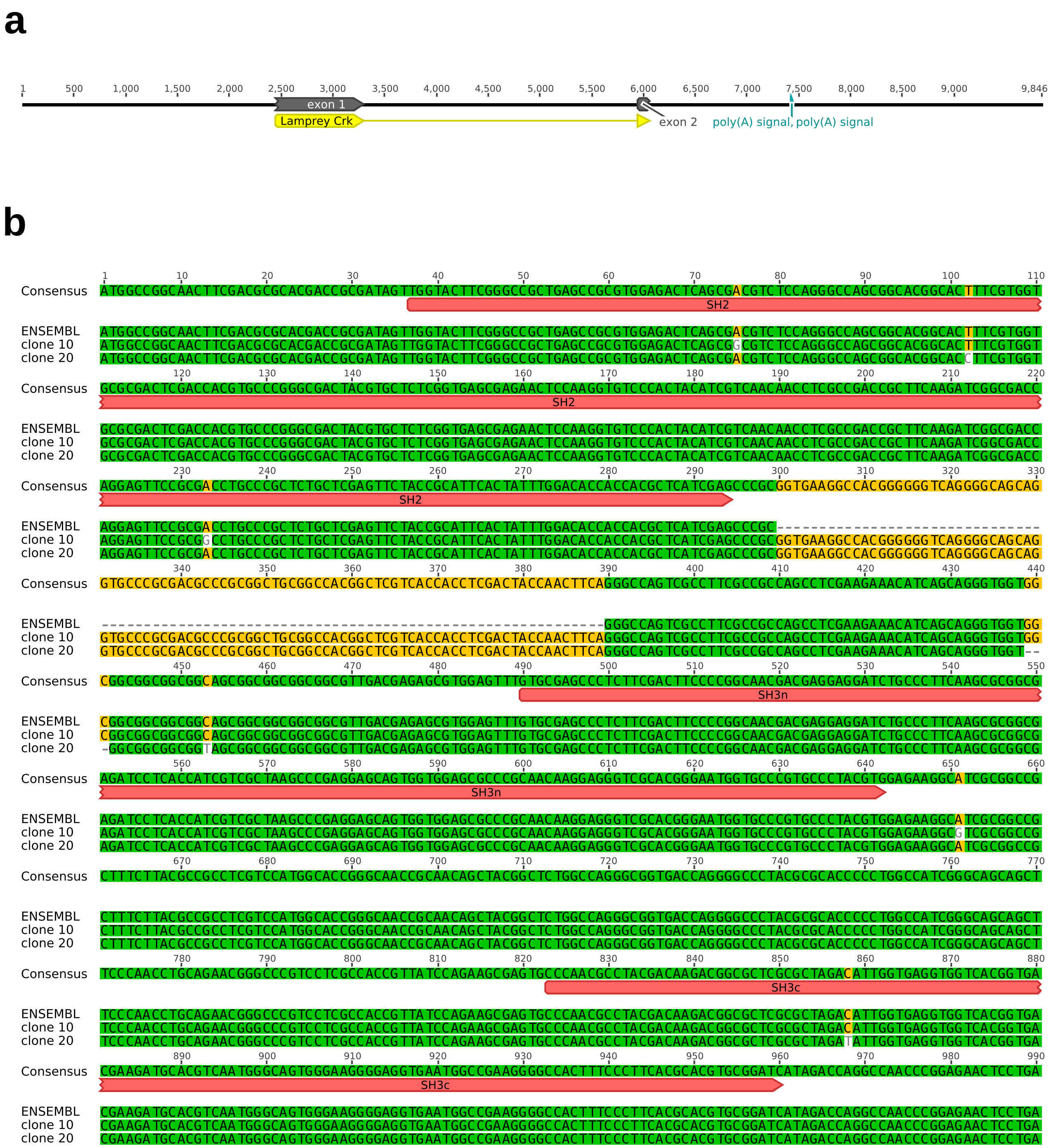

Supplementary Figure S3 Two *crka* sequences expressed in *Branchiostoma floridae*

Shown is a protein alignment of amphioxus *crka1* and *crka2* sequences with human CRK and CRKL. A consensus sequence is shown on top of the alignment and the regions that correspond to SH2 and SH3 domains are highlighted by pink bars beneath the consensus sequence. % similarity is indicated by green, yellow, or no shades corresponding to 100%, between 60-80%, or below 60%, respectively. Note that *B. floridae crka*1 and *crka2* have sequences highly similar to human *CRK* and *CRKL*. As they are mapped to different genomic scaffolds, we conclude that they are transcribed from two independent genes (Table S1).

Supplementary Figure S3

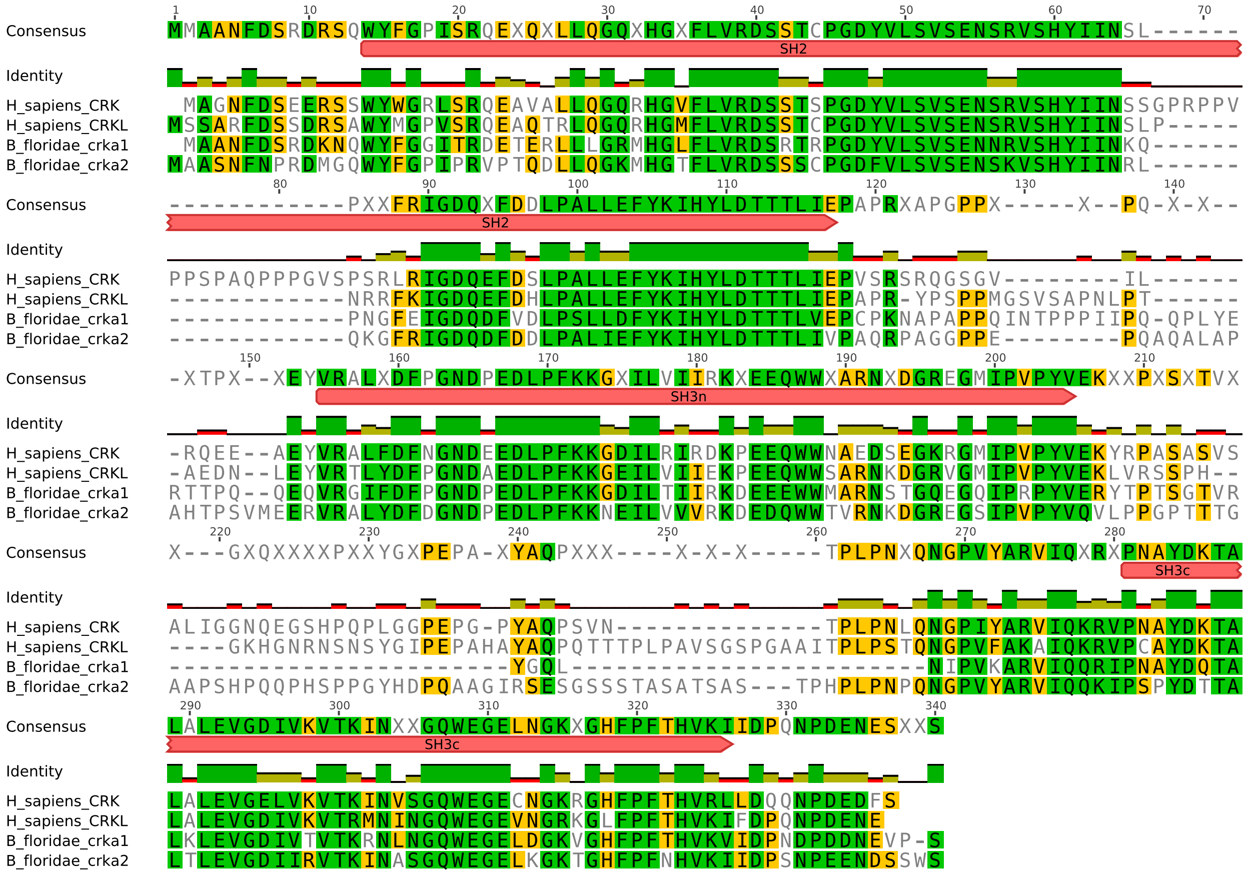

Supplementary Figure S4 *crka* sequence expressed in *Saccoglossus kowalevskii*

The region corresponding to the SH2 domain is extracted from a nucleotide sequence alignment of hemichordate *crka* and the model sequence XM_006822248 along with mammalian CRKL. For simplicity, mammalian CRK sequences are not included in the alignment. Translated amino acids are shown beneath each nucleotide sequence. Nucleotides in green, yellow, or no shades indicate % identity at 100%, between 60-80%, or below 60%, respectively. Note that the model sequence does not align in the second half of the SH2 domain (highlighted by a thick underline), while the transcribed *crka* shows a high degree of homology to mammalian sequences.

Supplementary Figure S4

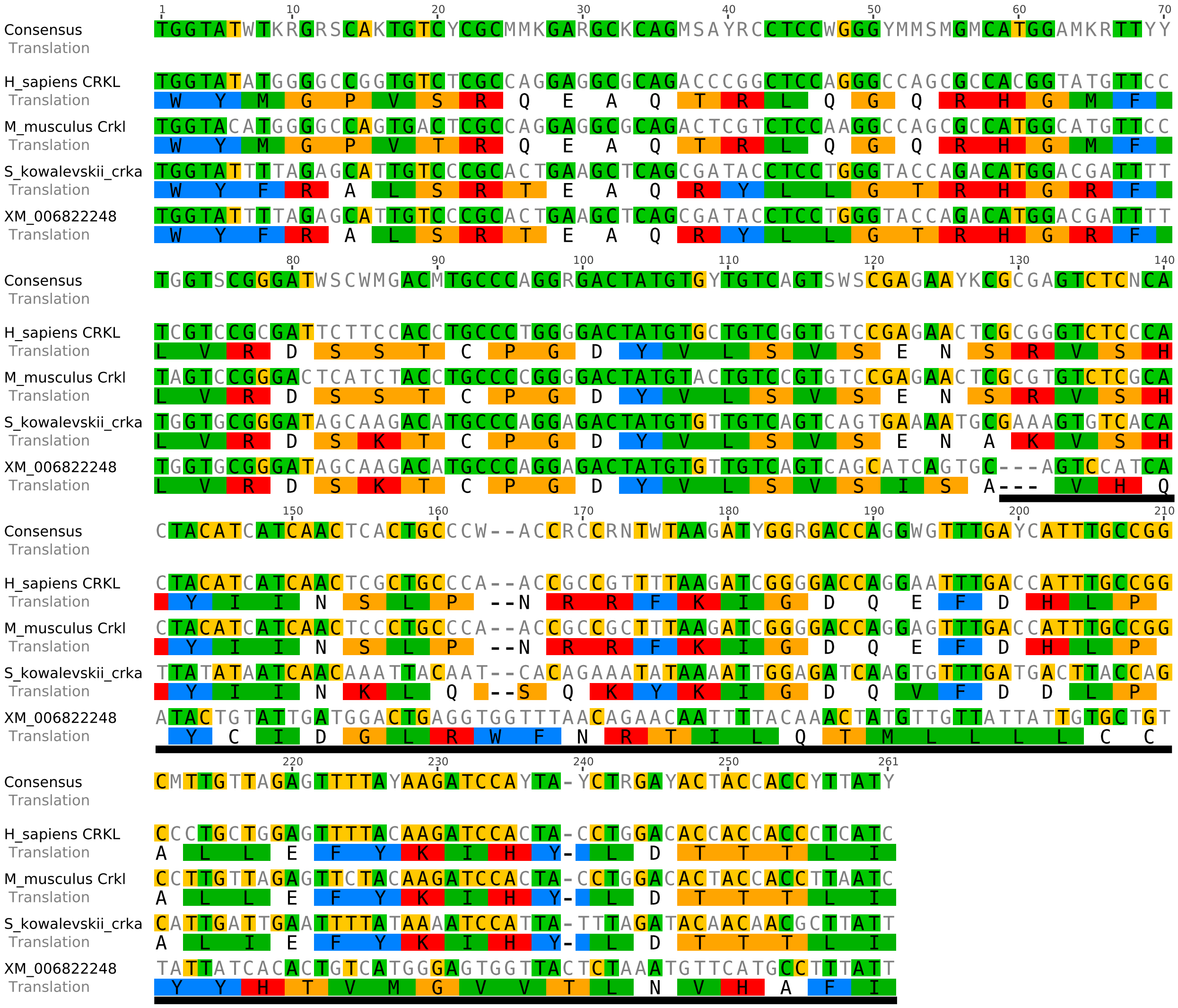

Supplementary Figure S5 Possible *crka* gene in *Trichoplax adhaerens*

(a) A graphic display of a possible *crka* locus in the placozoa. We have used GENSCAN to predict exons shown in gray boxes. The numbers along the line are the position of base(s) in the sequence shown (starting from 1) or in the genomic scaffold ABGP01000065 (starting from 239,401). Yellow color indicates the positions of possible coding sequences. Potential poly(A) signal is shown in turquoise.

(b) The predicted *Trichoplax crka* coding sequence is translated into an amino acid sequence and aligned with other *crka* sequences in the sea anemone *Nematostella vectensis* and the sponge *Amphimedon queenslandica* along with human CRK and CRKL. The SH2 and SH3 domains are highlighted in pink bars underneath the consensus. Amino acid residues in green, light green, yellow, or no shades indicate 100%, 80-100%, 60-80% or below 60% similarities, respectively. Note that predicted *Trichoplax crka* sequence shows a high degree of homology with other *crka* and human homologs in the SH2 and SH3 domains.

Supplementary Figure S5

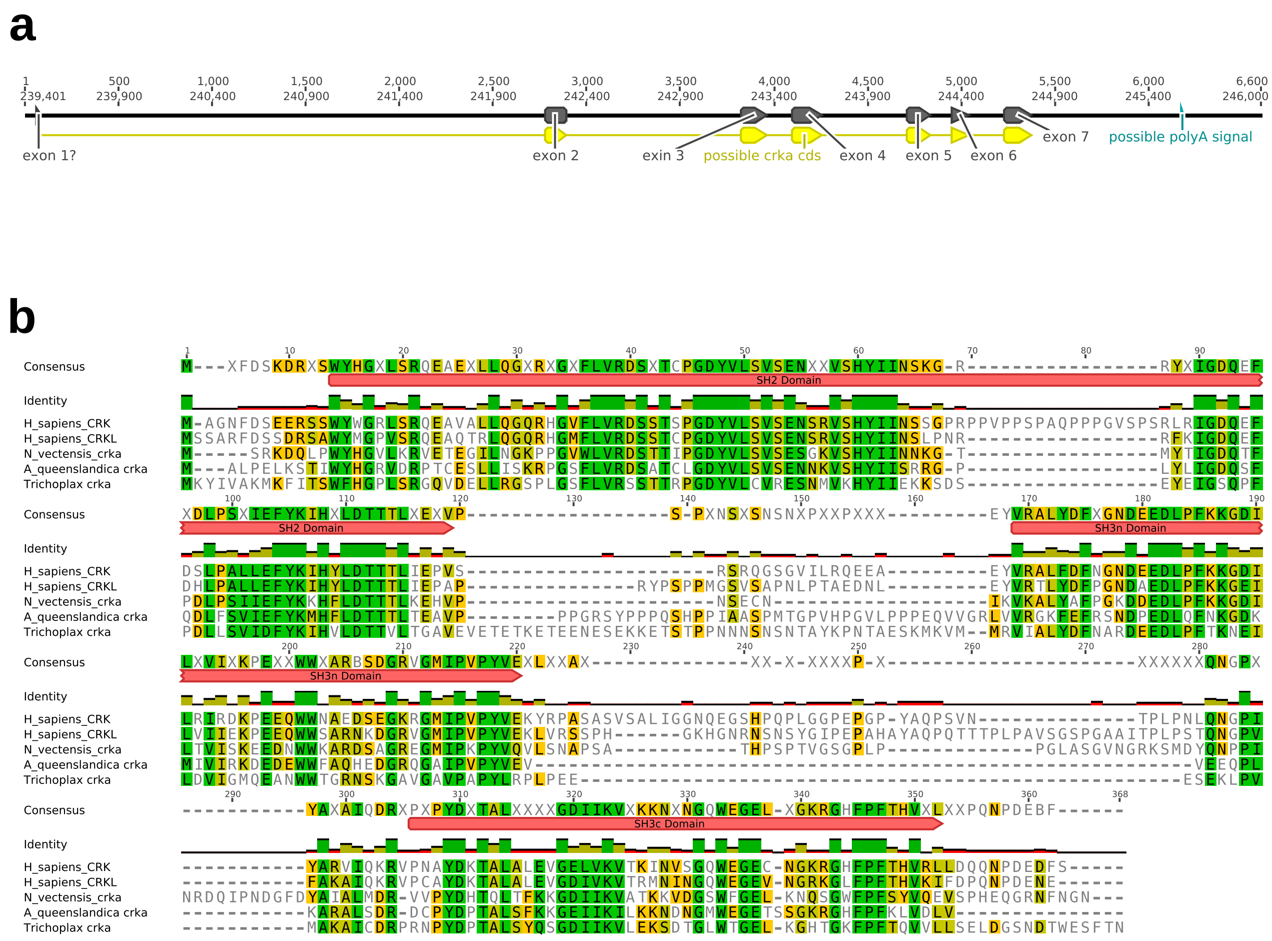

Supplementary Figure S6 Multiple sequence alignments for CRK, CRKL, and their orthologs

(a) A nucleotide sequence alignment was generated by GUIDANCE2 with MAFFT sequence aligner (E-INS-i). % identity is categorized into 4 groups by green, yellow-green, yellow, and gray representing 100%, 80-100%, 60-80%, or below 60%, respectively. The regions encoding the SH2 and SH3 domains are indicated by pink bars beneath the consensus. Thin gray lines indicate gaps. Note that the regions corresponding to SH2 and SH3 domains show largely green and yellow indicating high similarities, with the exception that *M. brevicollis crka2* shows a poor alignment in the SH3c domain (see also panel d).

(b-d) Panels b, c, and d show parts of the alignment corresponding to the SH2, SH3n, and SH3c, respectively. Translated amino acids are shown beneath each nucleotide sequence. % identity of each base is categorized into 4 groups by black, dark gray, pale gray, and no shades representing 100%, 80-100%, 60-80%, or below 60%, respectively. The vertebrate CRK SH2 domain has a long divergent DE loop in the protein structure (panel b; see also Figure 3). There is a conserved exon-exon junction at the end of the DE loop in the vertebrate CRK SH2 domain in *H. sapiens*, *M. musculus*, *G. gallus*, *X. tropicalis*, and *D. rerio,* but not in *L. erinaccea* (red arrow and line, panel b). The SH2 domain in all vertebrate *CRKL,* as well as in *C. intestinalis* and pre-metazoan *crka,* are encoded in a single exon. The red box in panel b highlights the codon encoding a choanoflagellate-specific basic amino acid residue (K56) shown in Figure 4. Note that the alignment matches perfectly with the reading frame of the SH2 and SH3n domains in all species (b and c). In panel d, the red arrow and line indicate the position of an exon-exon junction highly conserved in all species, except that i) *B. floridae crka1* and *crka2* have their SH3c domain encoded entirely by the last coding exon and that ii) *C. hemisphaerica crka* have not been mapped to genomic sequences. The blue arrow and line indicate the position of additional exon-exon junction that exists in *S. rosetta crka* and *M. brevicollis crka1*. Note that *M. brevicollis* crka2

shows a frameshift due to an inclusion of an unspliced unaligned sequence, and is therefore lacking a large part of an SH3c domain (d; see also Figure 1). Except for *M. brevicollis crka2*, the nucleotide alignment of the SH3c domain is consistent with the reading frame in all other species.

Supplementary Figure S6

a

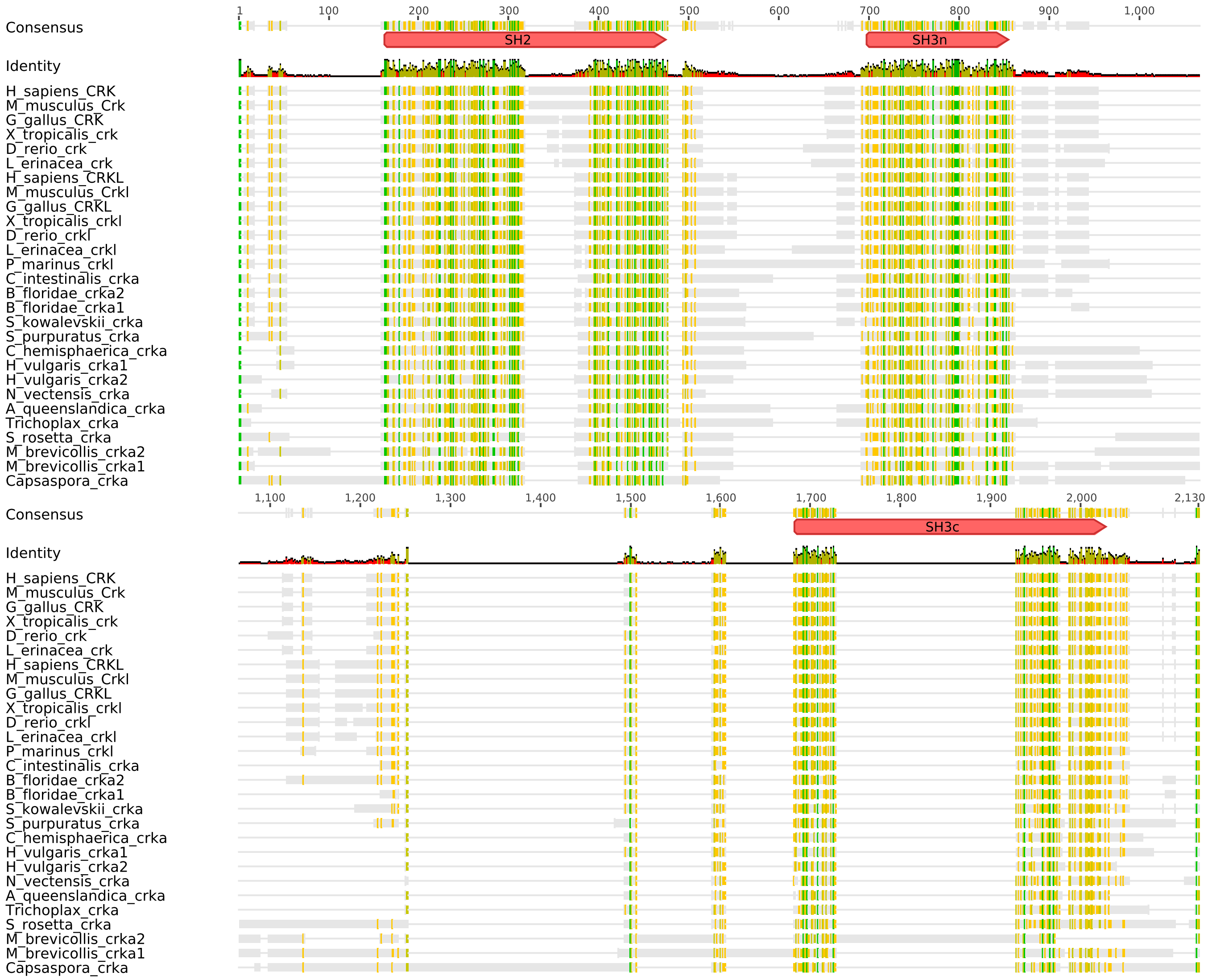

Supplementary Figure S6

b

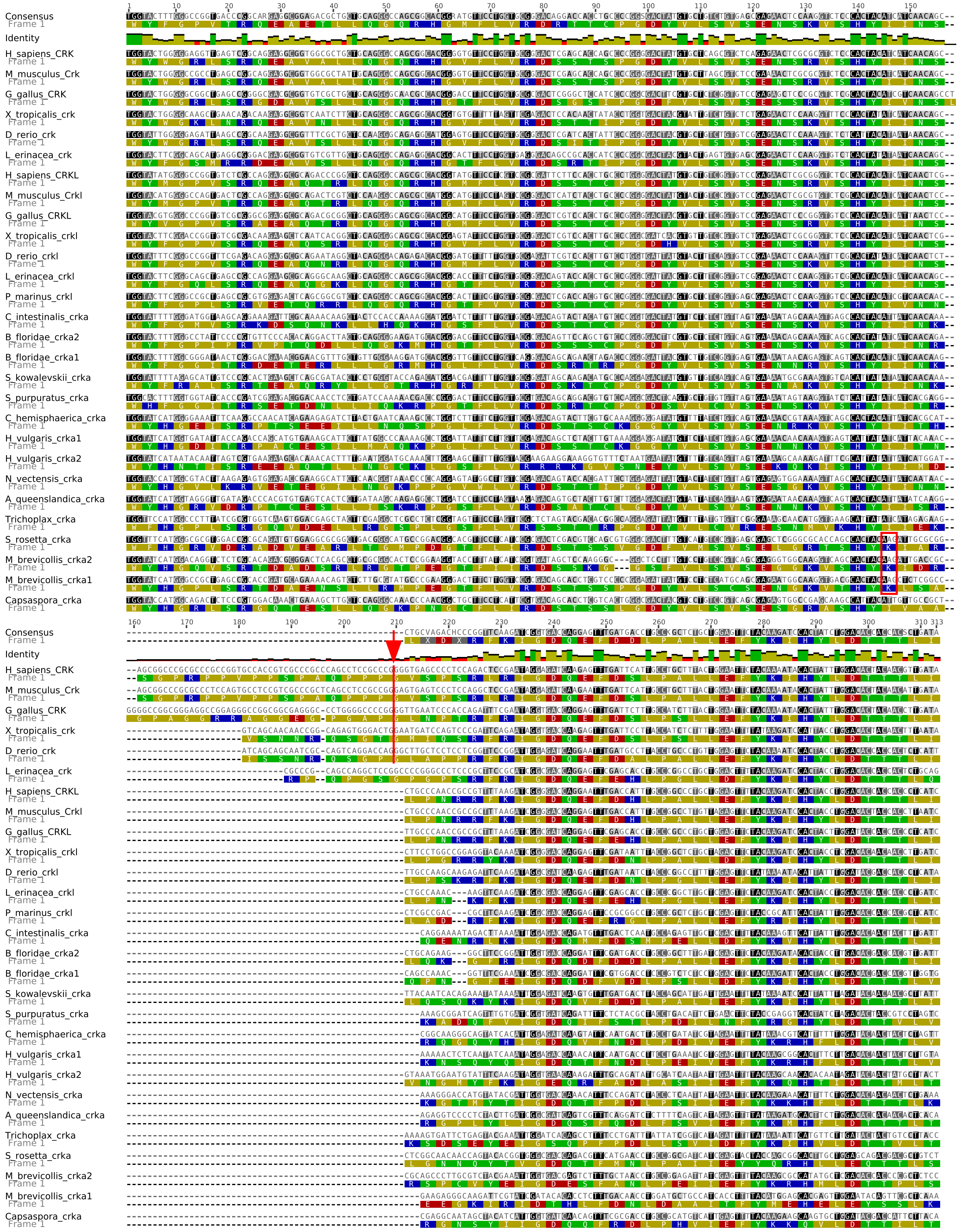

Supplementary Figure S6

c

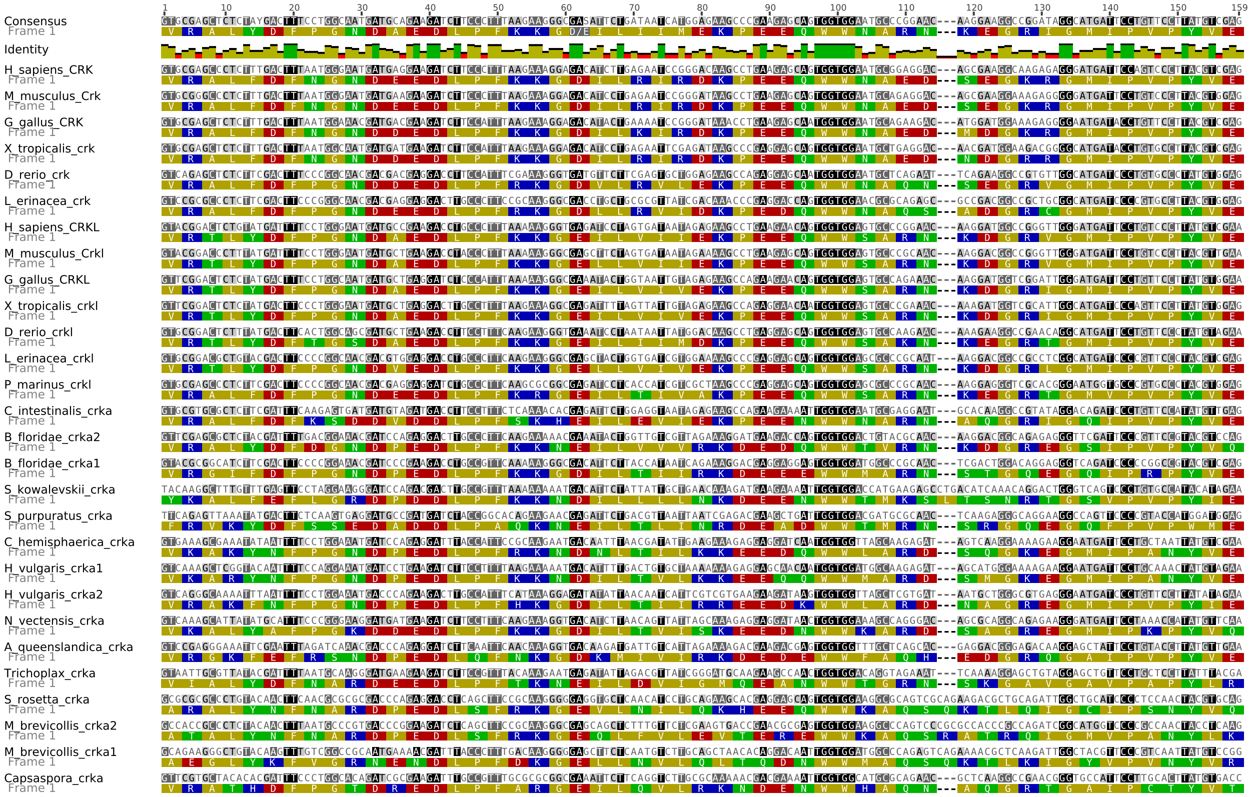

Supplementary Figure S6

d

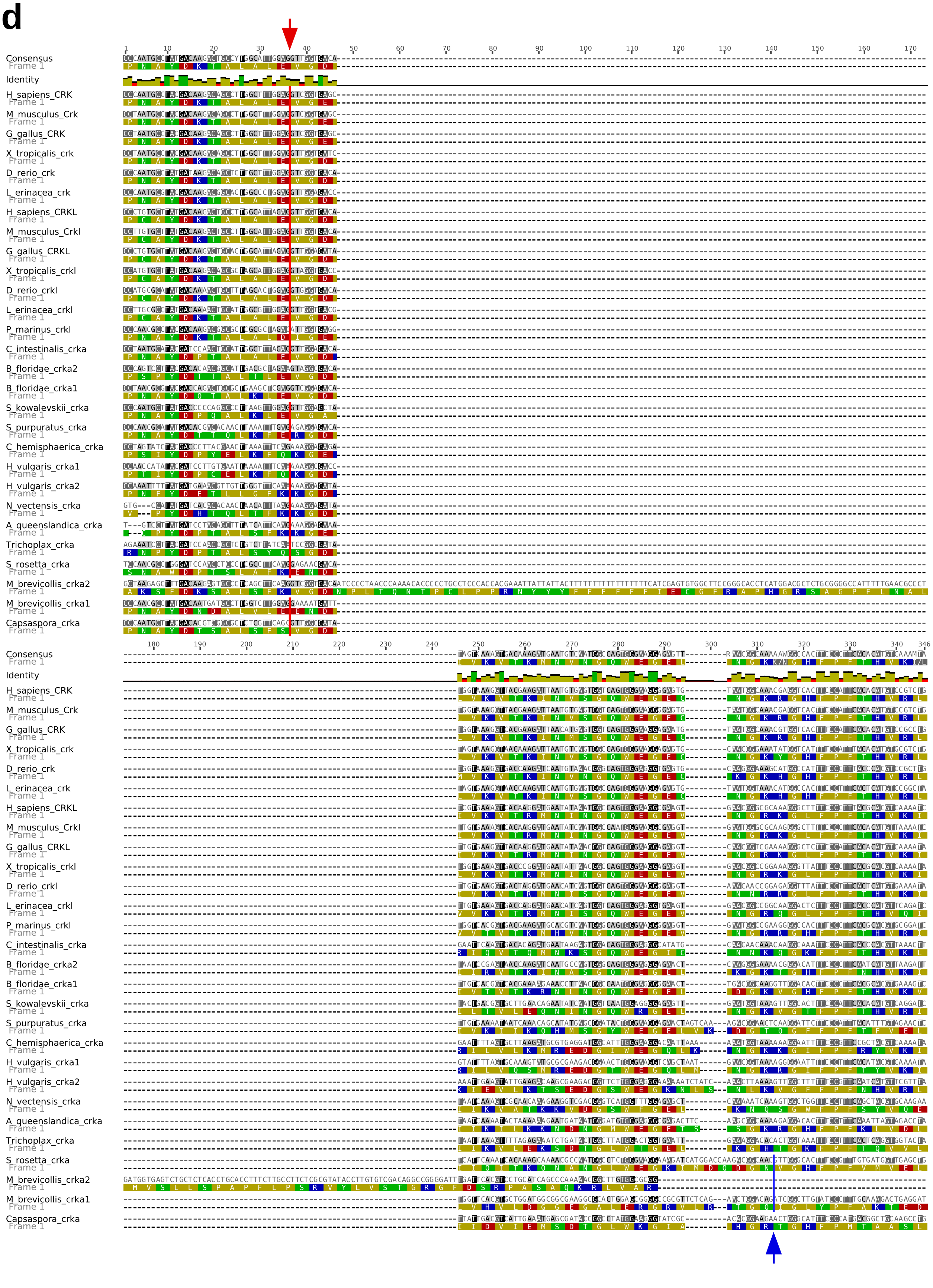

Supplementary Figure S7 Phylogenetic tree analysis

(a) Shown is a consensus Bayes tree using the same GUIDANCE2/MAFFT nucleotide alignment used in Figure 2 (see Supplementary Figure S6). Compared to the best-scored maximum likelihood tree shown in Fig. 2, consensus outputs from Bayes are drawn to average branch lengths. Therefore, the apparent multi-furcation at the base of the chordate speciation and the basal metazoans is not necessarily an indication of hard polytomy. Numbers at the base of each clade indicate posterior probabilities.

(b) Shown is a consensus Bayes tree using a protein alignment generated by GUIDANCE2/MAFFT aligner with E-INS-i algorithm, after poorly aligned columns are removed at a confidence score cutoff of 0.93. Numbers at the base of each clade indicate posterior probabilities

(c) A maximum likelihood (ML) tree was drawn using the same protein alignment used in panel b. Numbers at the base of each clade indicate bootstrap numbers. Note that ML or Bayes produced similar trees using the protein alignment, in which the placement of *A. queenslandica* crka (highlighted in red) appeared to be out of place considering the divergence of the species. The protein trees also produced a topology in which *B. floridae* crka1 and crka2 (highlighted with orange color) was placed before the hemichordate *S. kowalevskii* and echinoderm *S. purpuratus*. In addition, protein ML tree shows *N. vectensis* crka before *A. queenslandica* or *Trichoplax* crka. In contrast, trees generated from the nucleotide alignment either by ML or Bayes were in agreement with the current understanding of species evolution (Figure 2 and Supplementary Figure S7a).

Supplementary Figure S7

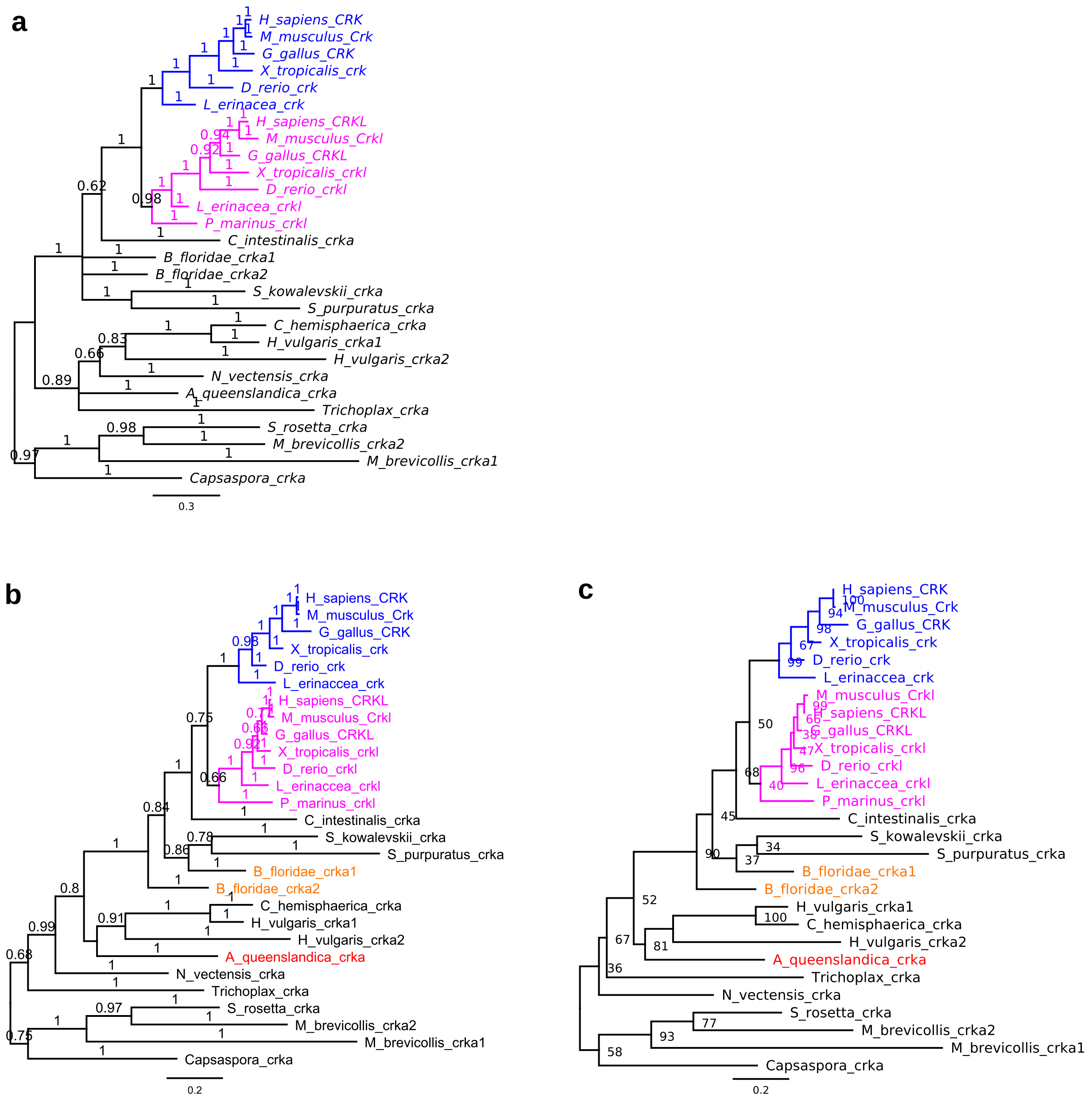

Supplementary Figure S8   An Atypical Amino Acid Residue in the EF Loop β-turn in *Monosiga brevicollis* crka1 SH2 Domain

(a) A stereo view of the β turn at EF loop of the mean structure of the SH2 domain of *M. brevicollis* crka1.

(b) Ramachandran diagram of D67 residue of 20 lowest energy structures. Shaded regions indicate "disallowed region" of the program PROCHECK. Black dots indicate experimental data.

(c) Amino-acid sequence alignment of the EF loop in SH2 domains from various species. Asterisk indicates i+1 position of the β turn.

Supplementary Figure S8

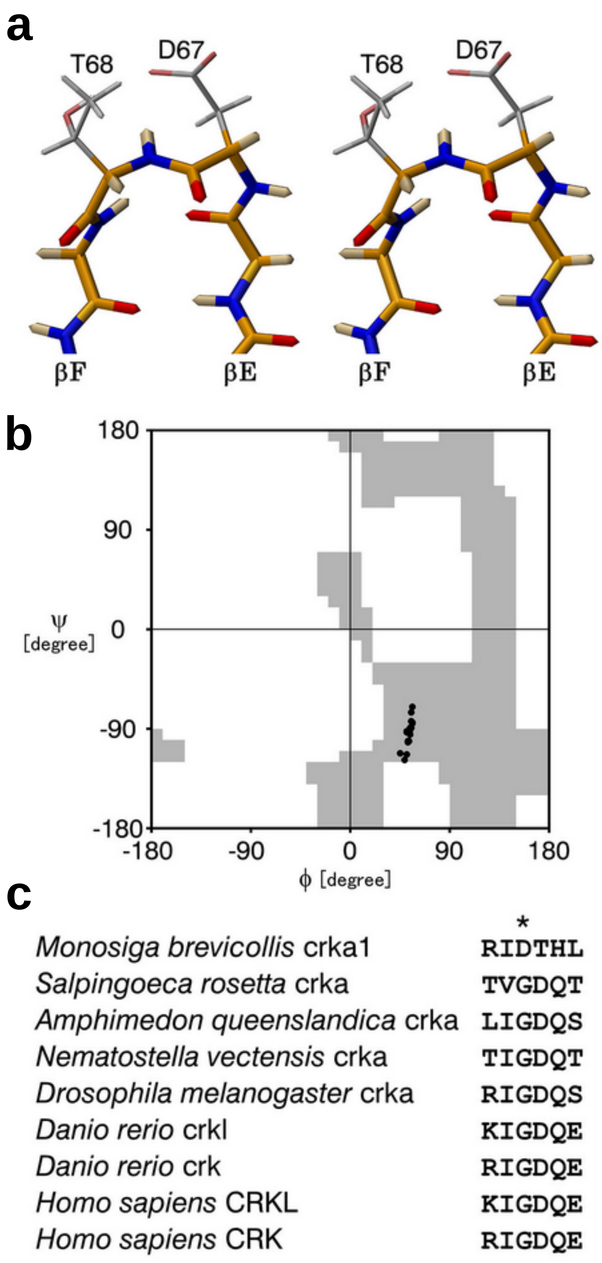

Supplementary Figure S9 Titration curves from isothermal titration calorimetry experiments

Each panel shows titration curves from isothermal titration calorimetry (ITC) experiments using SH2 domains and peptides. Red dots are the control experiment titrating the phosphorylated peptide against the blank buffer without the SH2 domain. Black dots fitted with red curve indicate titration with phosphorylated peptide. In general, a steep curve fitting suggests a stronger protein-protein interaction. Stable molecular binding can be heat generating or heat-absorbing reactions driven by a net change of entropy or enthalpy. Note that the K value in each inset is expressed as K_B (1/M). Table 1 summarizes affinities as the dissociation constant K_D (M).

	Human CRK SH2	Human CRKL SH2	MonBr crka1 SH2
Dok7 Y396	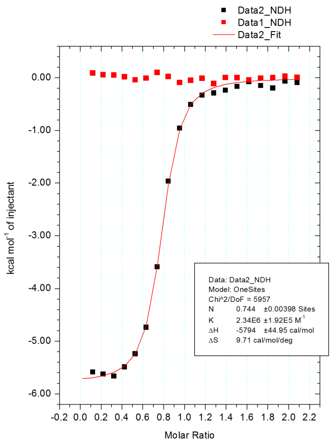	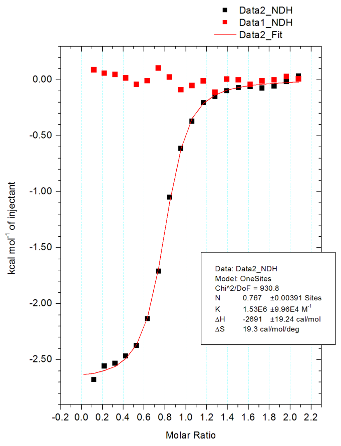	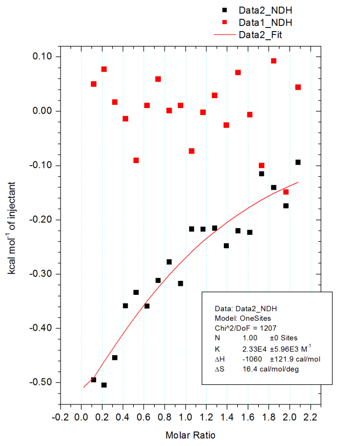
Dok7 Y406	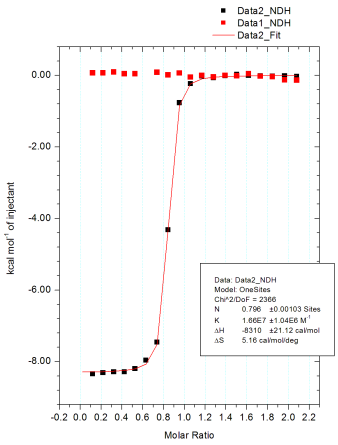	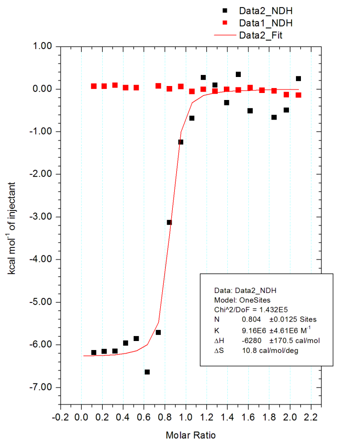	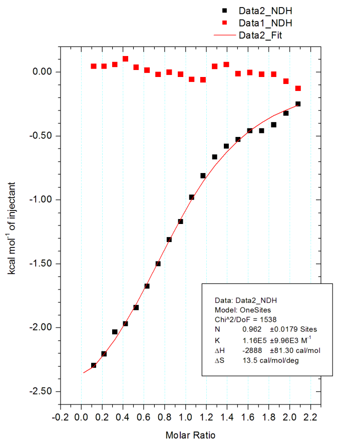
Bcar1 Y169	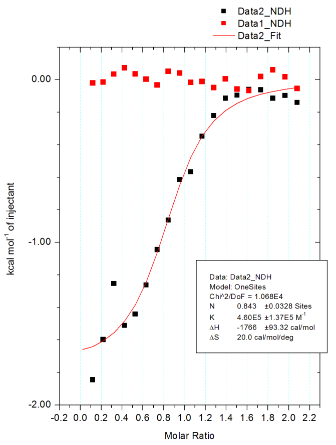	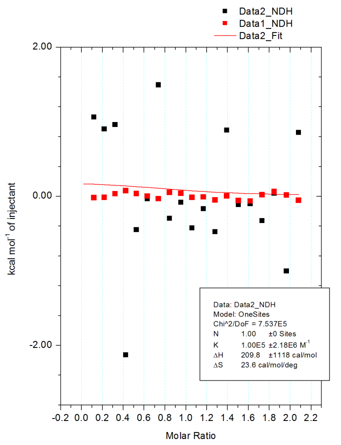	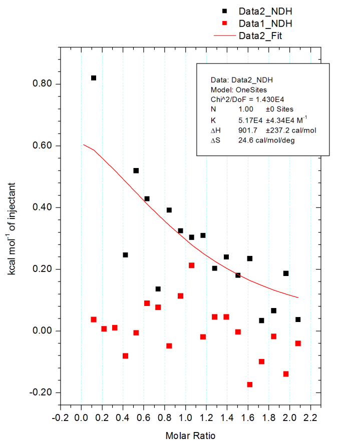

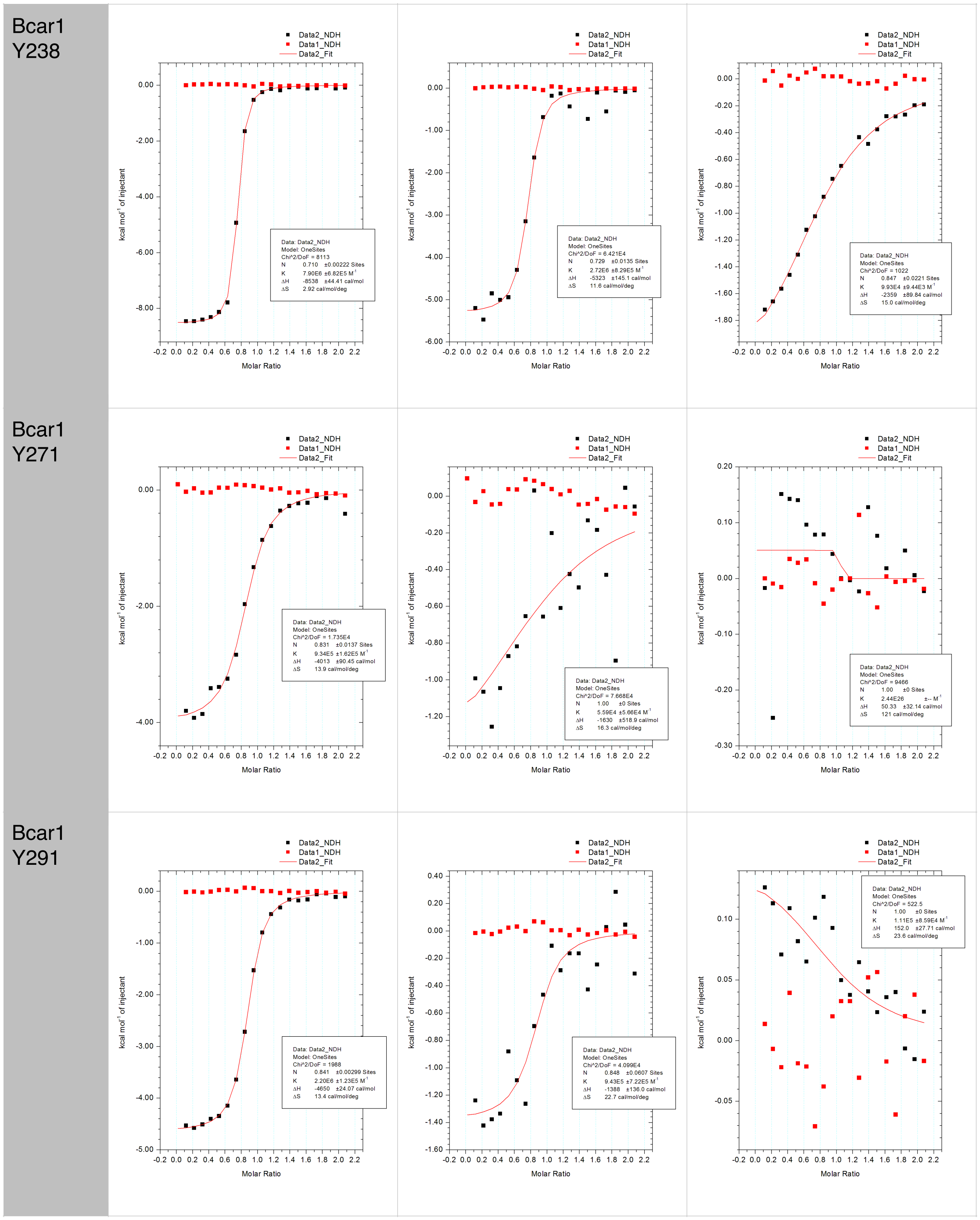
Bcar1 Y238
Data2_NDH
Data1_NDH
Data2_Fit
kcal mol-1 of injectant
Molar Ratio
Data: Data2_NDH
Model: OneSites
Chi^2/DoF = 8113
N 0.710 ±0.00222 Sites
K 7.90E6 ±6.82E5 M-1
ΔH -8538 ±44.41 cal/mol
ΔS 2.92 cal/mol/deg
Data: Data2_NDH
Model: OneSites
Chi^2/DoF = 6.421E4
N 0.729 ±0.0135 Sites
K 2.72E6 ±8.29E5 M-1
ΔH -5323 ±145.1 cal/mol
ΔS 11.6 cal/mol/deg
Data: Data2_NDH
Model: OneSites
Chi^2/DoF = 1022
N 0.847 ±0.0221 Sites
K 9.93E4 ±9.44E3 M-1
ΔH -2359 ±89.84 cal/mol
ΔS 15.0 cal/mol/deg
Bcar1 Y271
Data: Data2_NDH
Model: OneSites
Chi^2/DoF = 1.735E4
N 0.831 ±0.0137 Sites
K 9.34E5 ±1.62E5 M-1
ΔH -4013 ±90.45 cal/mol
ΔS 13.9 cal/mol/deg
Data: Data2_NDH
Model: OneSites
Chi^2/DoF = 7.668E4
N 1.00 ±0 Sites
K 5.59E4 ±5.66E4 M-1
ΔH -1630 ±518.9 cal/mol
ΔS 16.3 cal/mol/deg
Data: Data2_NDH
Model: OneSites
Chi^2/DoF = 9466
N 1.00 ±0 Sites
K 2.44E26 ±-- M-1
ΔH 50.33 ±32.14 cal/mol
ΔS 121 cal/mol/deg
Bcar1 Y291
Data: Data2_NDH
Model: OneSites
Chi^2/DoF = 1988
N 0.841 ±0.00299 Sites
K 2.20E6 ±1.23E5 M-1
ΔH -4650 ±24.07 cal/mol
ΔS 13.4 cal/mol/deg
Data: Data2_NDH
Model: OneSites
Chi^2/DoF = 4.099E4
N 0.848 ±0.0607 Sites
K 9.43E5 ±7.22E5 M-1
ΔH -1388 ±136.0 cal/mol
ΔS 22.7 cal/mol/deg
Data: Data2_NDH
Model: OneSites
Chi^2/DoF = 522.5
N 1.00 ±0 Sites
K 1.11E5 ±8.59E4 M-1
ΔH 152.0 ±27.71 cal/mol
ΔS 23.6 cal/mol/deg

Bcar1
Y310

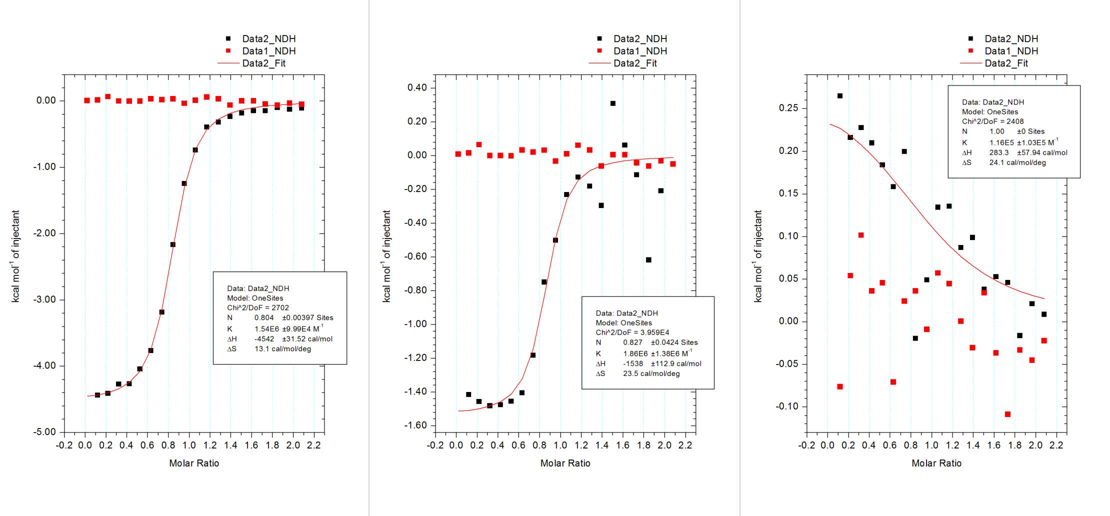

Bcar1
Y376

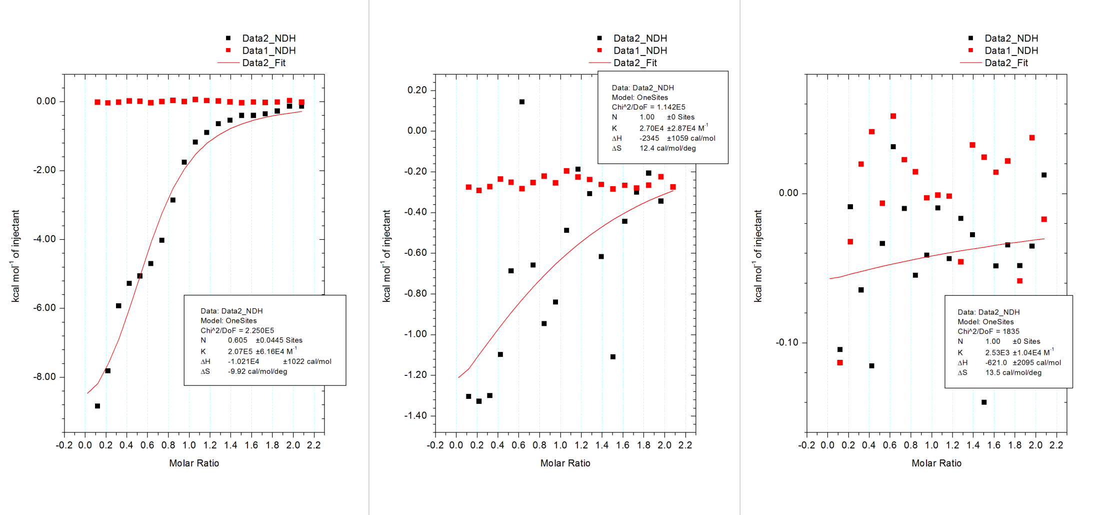

PXN
Y118

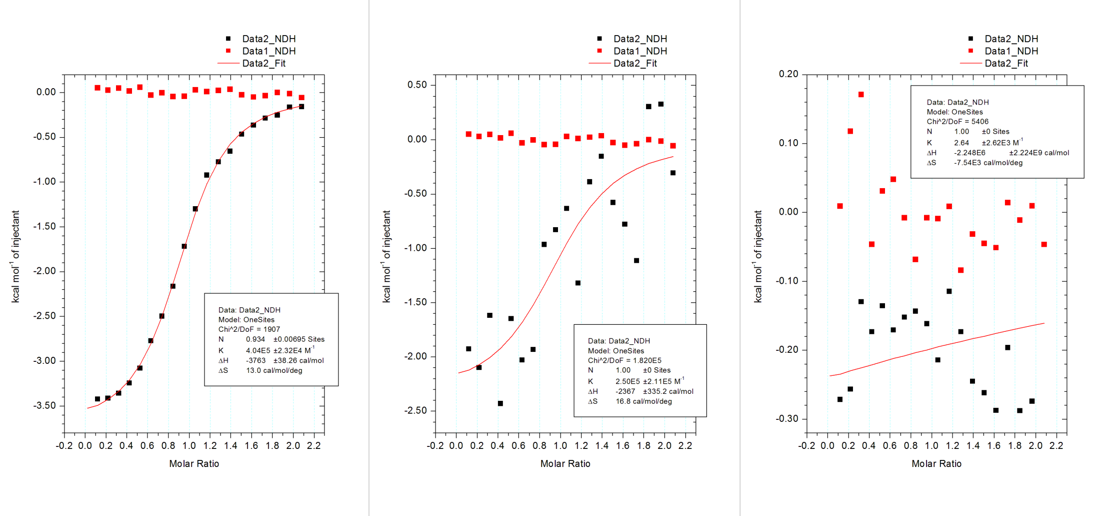

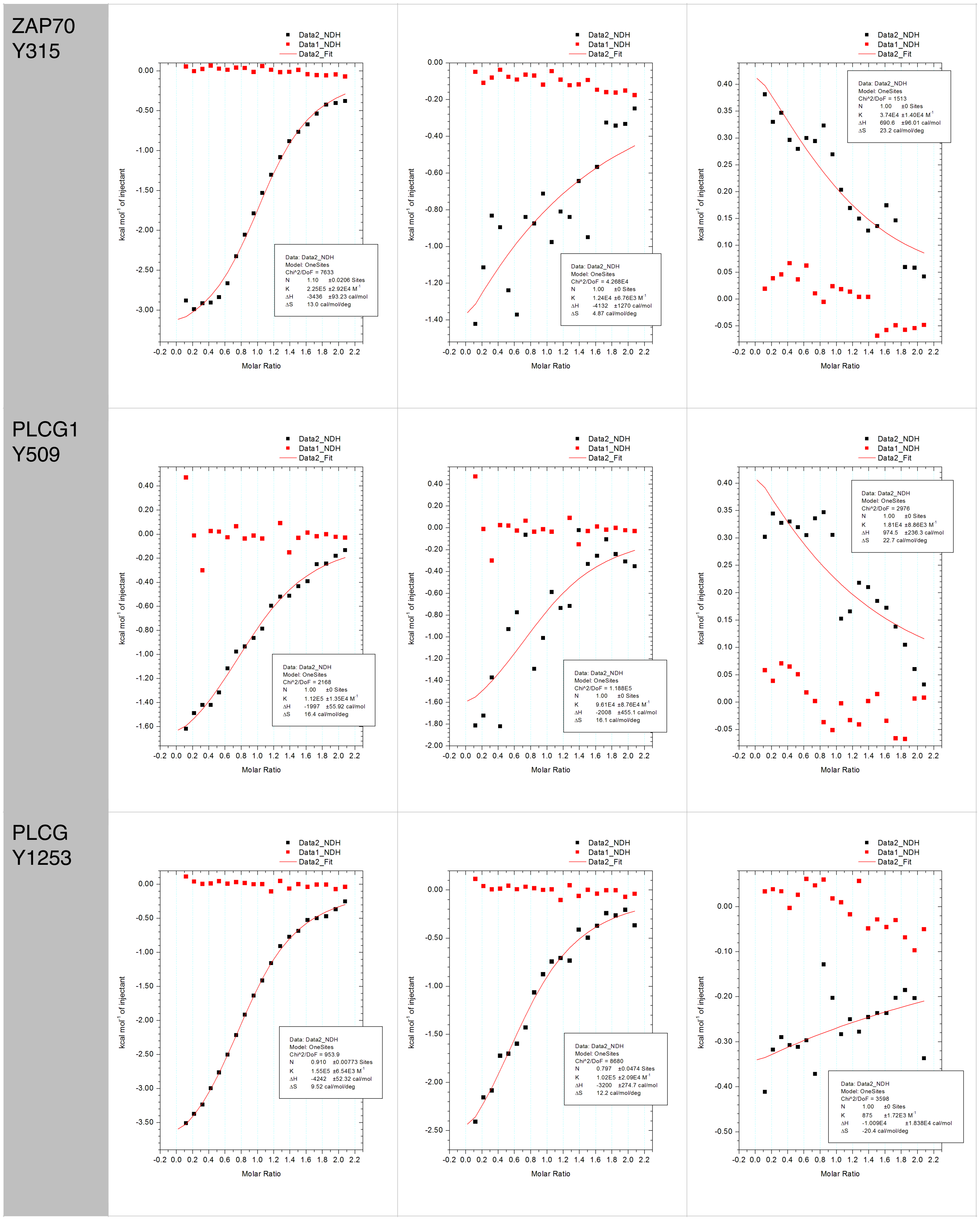
ZAP70
Y315
PLCG1
Y509
PLCG
Y1253
Data2_NDH
Data1_NDH
Data2_Fit
kcal mol-1 of injectant
Molar Ratio
Data: Data2_NDH
Model: OneSites
Chi^2/DoF = 7633
N 1.10 ±0.0206 Sites
K 2.25E5 ±2.92E4 M-1
ΔH -3436 ±93.23 cal/mol
ΔS 13.0 cal/mol/deg
Data: Data2_NDH
Model: OneSites
Chi^2/DoF = 4.268E4
N 1.00 ±0 Sites
K 1.24E4 ±6.76E3 M-1
ΔH -4132 ±1270 cal/mol
ΔS 4.87 cal/mol/deg
Data: Data2_NDH
Model: OneSites
Chi^2/DoF = 1513
N 1.00 ±0 Sites
K 3.74E4 ±1.40E4 M-1
ΔH 690.6 ±96.01 cal/mol
ΔS 23.2 cal/mol/deg
Data: Data2_NDH
Model: OneSites
Chi^2/DoF = 2168
N 1.00 ±0 Sites
K 1.12E5 ±1.35E4 M-1
ΔH -1997 ±55.92 cal/mol
ΔS 16.4 cal/mol/deg
Data: Data2_NDH
Model: OneSites
Chi^2/DoF = 1.188E5
N 1.00 ±0 Sites
K 9.61E4 ±8.76E4 M-1
ΔH -2008 ±455.1 cal/mol
ΔS 16.1 cal/mol/deg
Data: Data2_NDH
Model: OneSites
Chi^2/DoF = 2976
N 1.00 ±0 Sites
K 1.81E4 ±8.86E3 M-1
ΔH 974.5 ±236.3 cal/mol
ΔS 22.7 cal/mol/deg
Data: Data2_NDH
Model: OneSites
Chi^2/DoF = 953.9
N 0.910 ±0.00773 Sites
K 1.55E5 ±6.54E3 M-1
ΔH -4242 ±52.32 cal/mol
ΔS 9.52 cal/mol/deg
Data: Data2_NDH
Model: OneSites
Chi^2/DoF = 8680
N 0.797 ±0.0474 Sites
K 1.02E5 ±2.09E4 M-1
ΔH -3200 ±274.7 cal/mol
ΔS 12.2 cal/mol/deg
Data: Data2_NDH
Model: OneSites
Chi^2/DoF = 3598
N 1.00 ±0 Sites
K 875 ±1.72E3 M-1
ΔH -1.009E4 ±1.838E4 cal/mol
ΔS -20.4 cal/mol/deg

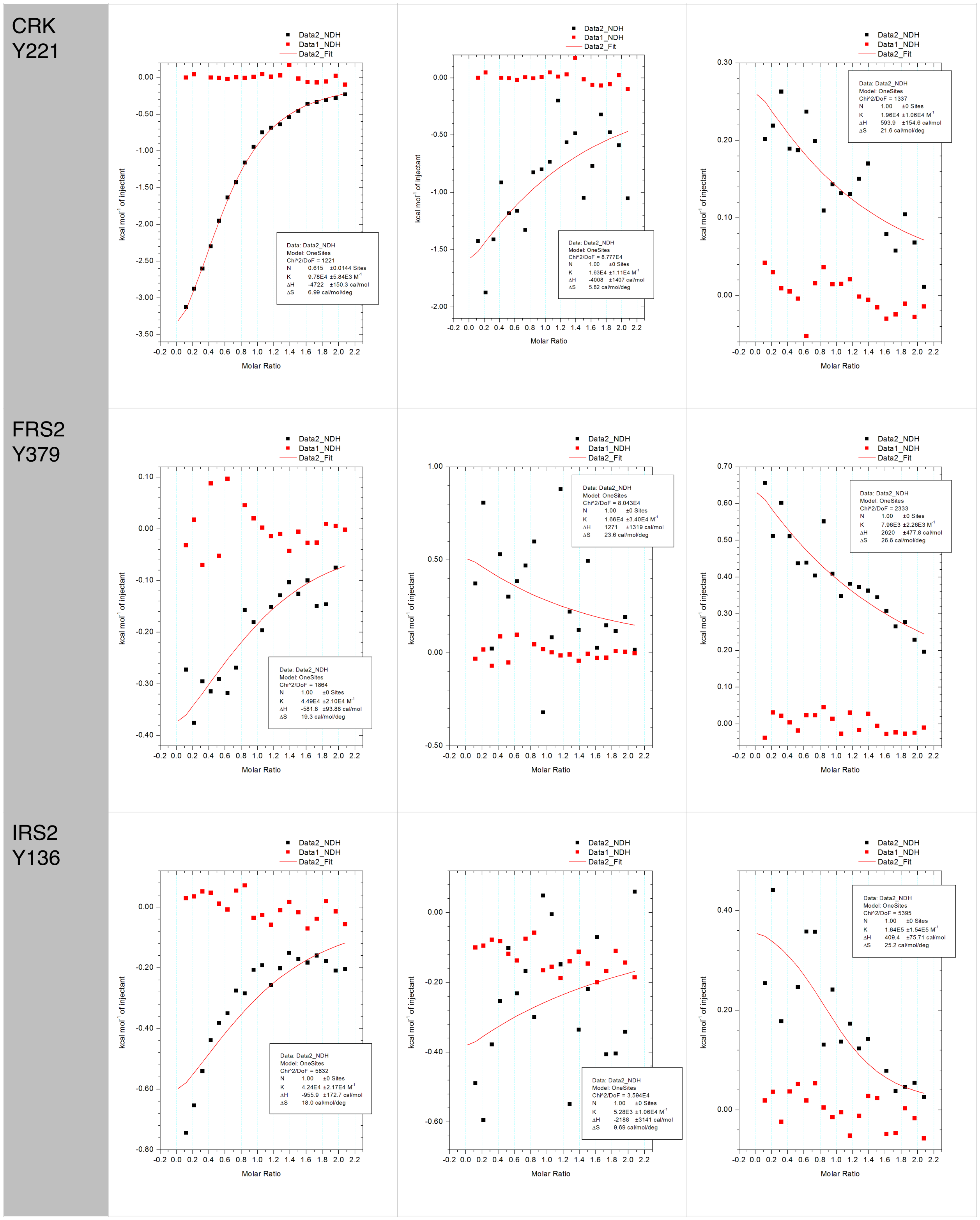

CRK
Y221
FRS2
Y379
IRS2
Y136
Data2_NDH
Data1_NDH
Data2_Fit
kcal mol-1 of injectant
Molar Ratio
Data: Data2_NDH
Model: OneSites
Chi^2/DoF = 1221
N 0.615 ±0.0144 Sites
K 9.78E4 ±5.84E3 M-1
ΔH -4722 ±150.3 cal/mol
ΔS 6.99 cal/mol/deg
Data: Data2_NDH
Model: OneSites
Chi^2/DoF = 8.777E4
N 1.00 ±0 Sites
K 1.63E4 ±1.11E4 M-1
ΔH -4008 ±1407 cal/mol
ΔS 5.82 cal/mol/deg
Data: Data2_NDH
Model: OneSites
Chi^2/DoF = 1337
N 1.00 ±0 Sites
K 1.96E4 ±1.06E4 M-1
ΔH 593.9 ±154.6 cal/mol
ΔS 21.6 cal/mol/deg
Data: Data2_NDH
Model: OneSites
Chi^2/DoF = 1864
N 1.00 ±0 Sites
K 4.49E4 ±2.10E4 M-1
ΔH -581.8 ±93.88 cal/mol
ΔS 19.3 cal/mol/deg
Data: Data2_NDH
Model: OneSites
Chi^2/DoF = 8.043E4
N 1.00 ±0 Sites
K 1.66E4 ±3.40E4 M-1
ΔH 1271 ±1319 cal/mol
ΔS 23.6 cal/mol/deg
Data: Data2_NDH
Model: OneSites
Chi^2/DoF = 2333
N 1.00 ±0 Sites
K 7.96E3 ±2.26E3 M-1
ΔH 2620 ±477.8 cal/mol
ΔS 26.6 cal/mol/deg
Data: Data2_NDH
Model: OneSites
Chi^2/DoF = 5832
N 1.00 ±0 Sites
K 4.24E4 ±2.17E4 M-1
ΔH -955.9 ±172.7 cal/mol
ΔS 18.0 cal/mol/deg
Data: Data2_NDH
Model: OneSites
Chi^2/DoF = 3.594E4
N 1.00 ±0 Sites
K 5.28E3 ±1.08E4 M-1
ΔH -2188 ±3141 cal/mol
ΔS 9.69 cal/mol/deg
Data: Data2_NDH
Model: OneSites
Chi^2/DoF = 5395
N 1.00 ±0 Sites
K 1.64E5 ±1.54E5 M-1
ΔH 409.4 ±75.71 cal/mol
ΔS 25.2 cal/mol/deg

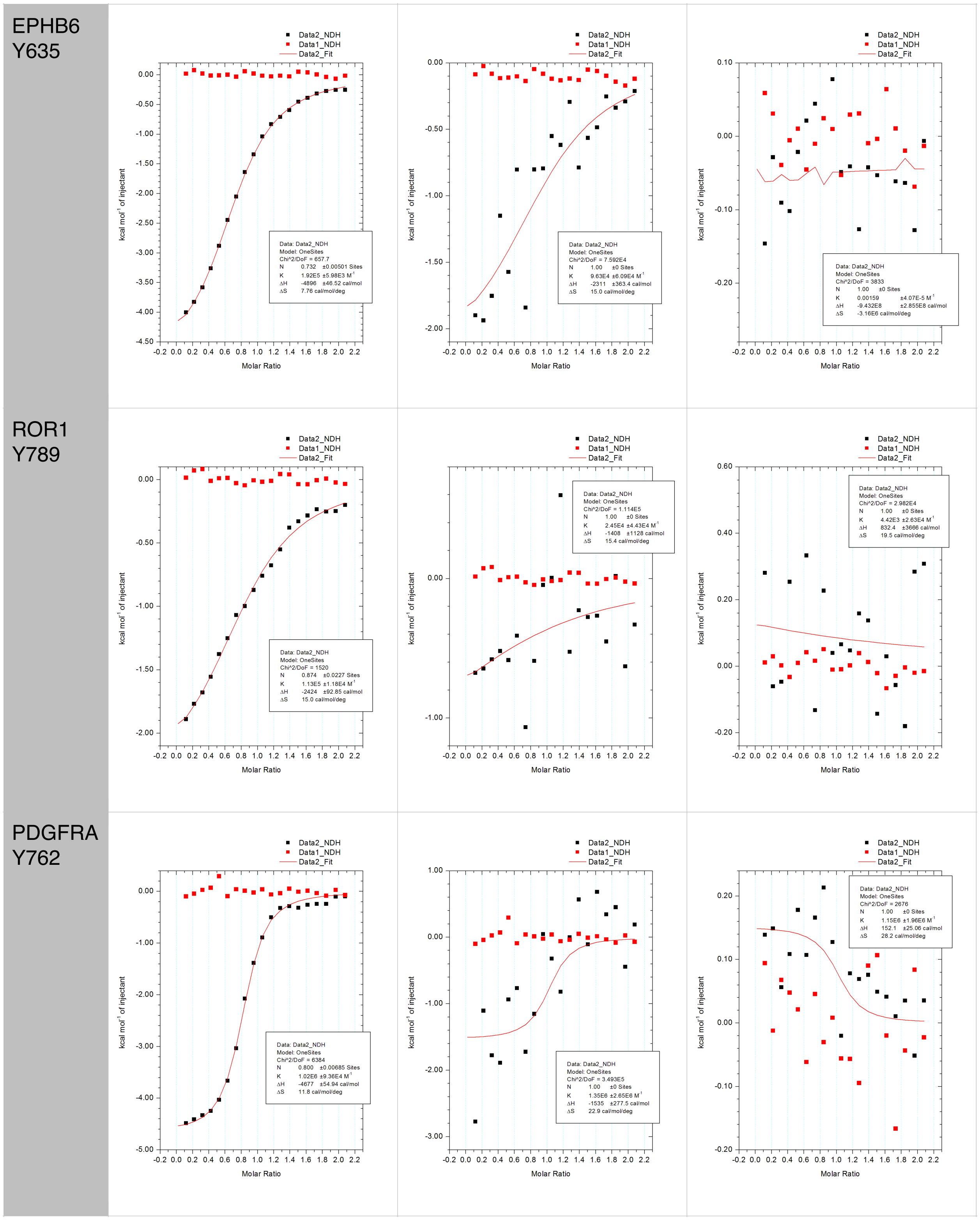
EPHB6
Y635
Data2_NDH
Data1_NDH
Data2_Fit
kcal mol-1 of injectant
Molar Ratio
Data: Data2_NDH
Model: OneSites
Chi^2/DoF = 657.7
N 0.732 ±0.00501 Sites
K 1.92E5 ±5.98E3 M-1
ΔH -4896 ±46.52 cal/mol
ΔS 7.76 cal/mol/deg
Data: Data2_NDH
Model: OneSites
Chi^2/DoF = 7.592E4
N 1.00 ±0 Sites
K 9.63E4 ±6.09E4 M-1
ΔH -2311 ±363.4 cal/mol
ΔS 15.0 cal/mol/deg
Data: Data2_NDH
Model: OneSites
Chi^2/DoF = 3833
N 1.00 ±0 Sites
K 0.00159 ±4.07E-5 M-1
ΔH -9.432E8 ±2.855E8 cal/mol
ΔS -3.16E6 cal/mol/deg
ROR1
Y789
Data: Data2_NDH
Model: OneSites
Chi^2/DoF = 1520
N 0.874 ±0.0227 Sites
K 1.13E5 ±1.18E4 M-1
ΔH -2424 ±92.85 cal/mol
ΔS 15.0 cal/mol/deg
Data: Data2_NDH
Model: OneSites
Chi^2/DoF = 1.114E5
N 1.00 ±0 Sites
K 2.45E4 ±4.43E4 M-1
ΔH -1408 ±1128 cal/mol
ΔS 15.4 cal/mol/deg
Data: Data2_NDH
Model: OneSites
Chi^2/DoF = 2.982E4
N 1.00 ±0 Sites
K 4.42E3 ±2.63E4 M-1
ΔH 832.4 ±3666 cal/mol
ΔS 19.5 cal/mol/deg
PDGFRA
Y762
Data: Data2_NDH
Model: OneSites
Chi^2/DoF = 6384
N 0.800 ±0.00685 Sites
K 1.02E6 ±9.36E4 M-1
ΔH -4677 ±54.94 cal/mol
ΔS 11.8 cal/mol/deg
Data: Data2_NDH
Model: OneSites
Chi^2/DoF = 3.493E5
N 1.00 ±0 Sites
K 1.35E6 ±2.65E6 M-1
ΔH -1535 ±277.5 cal/mol
ΔS 22.9 cal/mol/deg
Data: Data2_NDH
Model: OneSites
Chi^2/DoF = 2676
N 1.00 ±0 Sites
K 1.15E6 ±1.96E6 M-1
ΔH 152.1 ±25.06 cal/mol
ΔS 28.2 cal/mol/deg

PDGFRB Y771

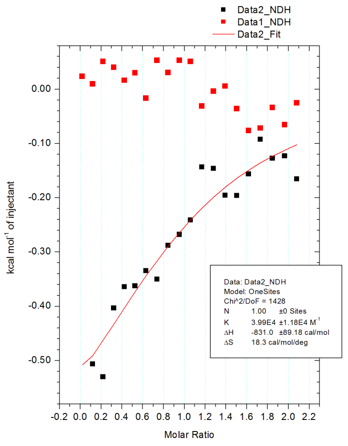

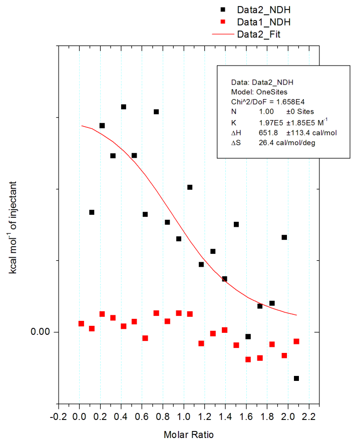

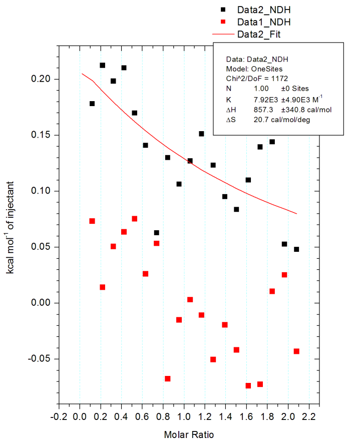

VEGFR2 (KDR) Y1054; VEGFR3 (FLT4) Y1063

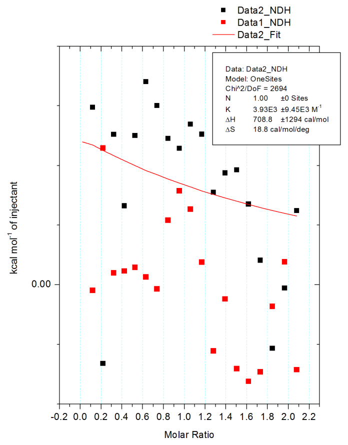

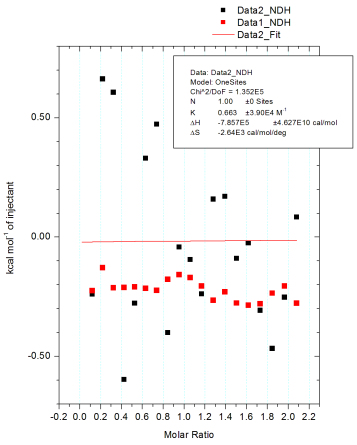

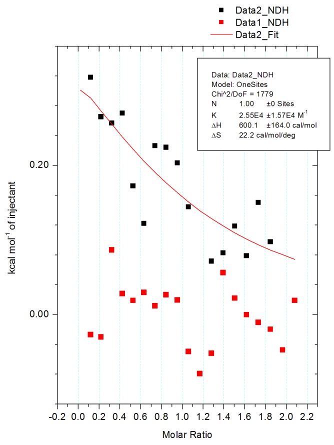

PTSG_05573 Y581, Y668, Y735

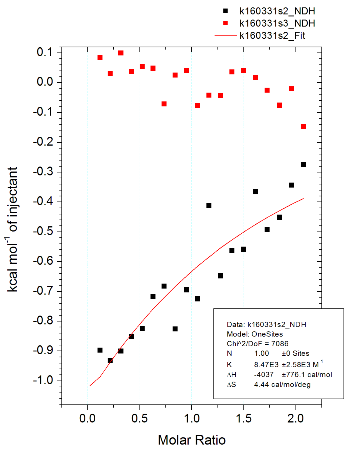

PTSG_05573 Y527, Y635			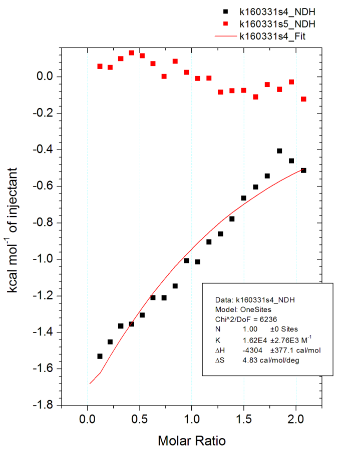

Supplementary Fig. S10   Alignment of Proteins that Include Multiple YxxP Motifs

(a) The diagram shows a graphical view of aligned bcar1 proteins from selected species including two basal metazoans, the sponge *Amphimedon queenslandica* and the placozoa *Trichoplax adhaerens*. Major domains found in mammalian BCAR1 are indicated as pink bars underneath the consensus. Green, yellow-green, yellow, and gray represent 100%, 80-100%, 60-80%, or below 60% similarities, respectively. Gray triangles under each sequence indicate the positions of YxxP motifs. The N-terminal SH3 domain is highly conserved, while the FAT-like domain is moderately conserved at the C-terminus (see % similarities in Supplementary Table S5). Although the substrate domains are aligned with many gaps, the presence of repeated YxxP motifs is an important feature conserved through evolution that is functionally essential in mammalian BCAR1.

(b) Shown is the bcar1 substrate domain that was re-aligned with *Salpingoeca rosetta* PTSG_05573 (XP_004993441). Conserved YxxP motifs are highlighted by purple bars beneath the consensus, while individual YxxP motifs are highlighted only in human and *S. rosetta* sequences for the sake of simplicity (gray bars). The putative *S. rosetta* protein has 10 YxxP motifs overlapping the 15 YxxP conserved motifs in this region. Human BCAR1 has 15 YxxP motifs, 14 of which overlap the 15 conserved motifs. % similarities are categorized into 4 groups: 100%, 80-100%, 60-80%, and below 60% by shades of green, yellow-green, yellow, and no shades, respectively. Although addition of *S. rosetta* PTSG_05573 has shifted relative positions of some YxxP motifs, the re-alignment highlights the presence of multiple YxxP motifs in *S. rosetta* PTSG_05573 similar to those of metazoan bcar1 orthologs. Note that the substrate domains are highly divergent, although all have several YxxP motifs as an important feature of this domain.

Supplementary Fig S10

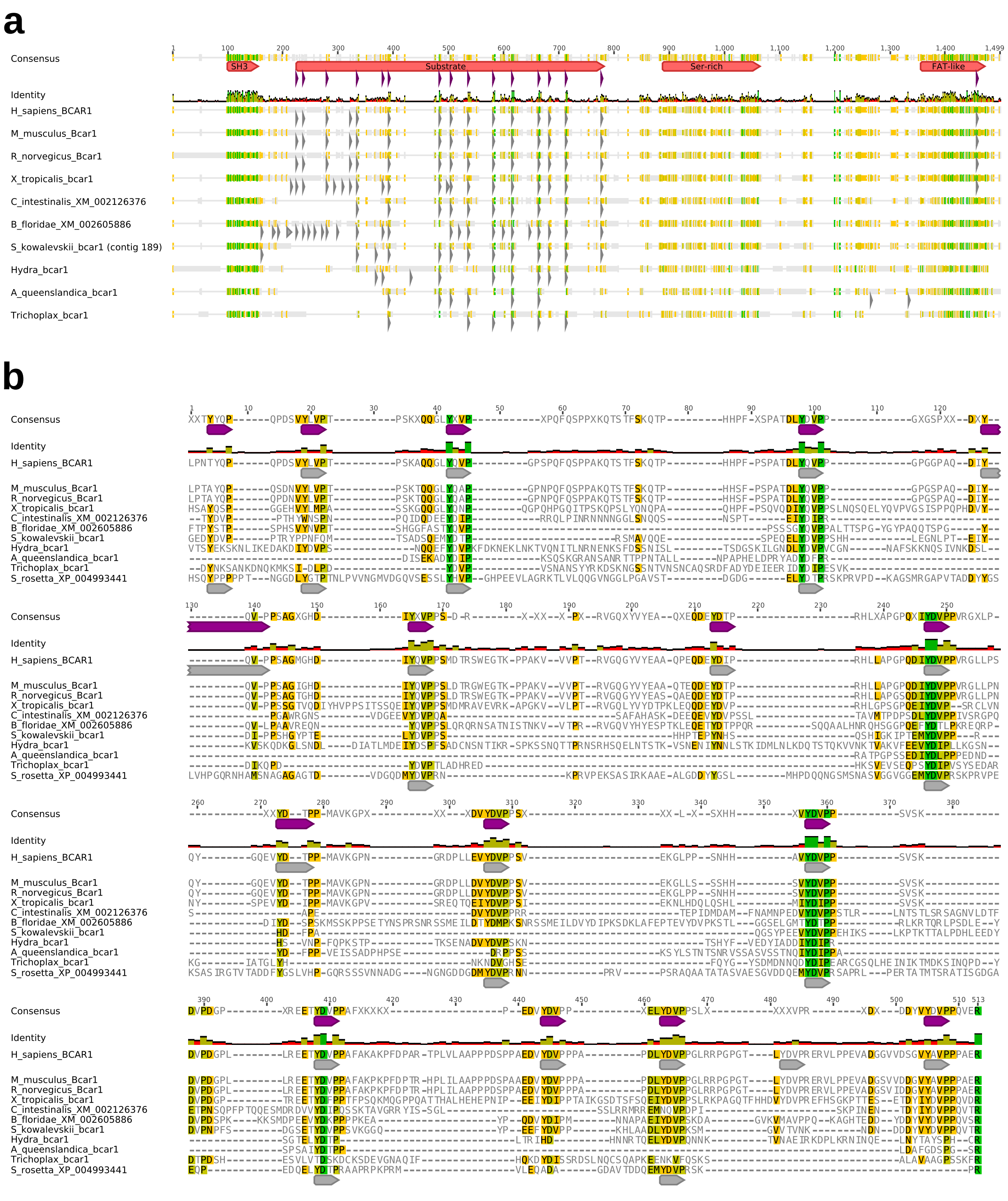

Supplementary Data 1: New Nucleotide Sequences Reported

Following are the CRK/CRKL orthologous mRNA sequences newly reported in this manuscript. See the Methods section of the main text and Supplementary Table S1 (highlighted in the boxes shaded in pink). To extract the sequence info in the FASTA format, select the header and nucleotide sequence including the non-letter character '>' and copy/paste into a new text file. After saving, replace '.txt' in the file name with '.fasta' or '.fas' before importing it to sequence analysis programs, if necessary.

```
>M_brevicollis_crka1 | KT795324 | MONBRDRAFT_25438 => contig 403 (clones 221, 223,
224)
TGCGCCTCGATCATTTTGAGCCCCAGGGCCTTTGTTGTGCATCATGGCGGAAGCCGCAGCACCCTGGTATCATGGGCCGCTGAGC
CGCACCGATGCAGAAAACAGTCTCTTGCGTATGCCCGAAGGGACTTTCTTGGTCCGCGACAGCACCTCGTCCCCCGGAGATTATG
TCCTCTCATGCAGCGAGAATGGCAAGGTGACGCACTACAAGCTCTCGGCCGAAGAGGGCAAGATTCGTATCGATACACACCTCTT
TGACAACCTGGATGCTGCCATCACCTTTTACATGGAGCACGAGTTGGAATACAGTTCGCTCAAACAGCCCCTGCAACGGCAATCA
TCATGGCGCGATGACGAGCTTGTCGCCACGGTCATGTTCTCTGCAGAAGGGCTGTACAAGTTTGTCGGCCGCAATGAAAACGATT
TACCCTTTGACAAGGGGGAGCTTCTCAATGTCTTGCAGCTAACACAGGACAATTGGTGGATGGCCCAGAGTCAGAAAACGCTCAA
GATTGGCTACGTTCCCGTCAATTATGTCCGGGCGCTAGAGCAGCCAGCTGCCCCGCCACCGCGCCCTGCTCCACGTCCTGCCGAG
GAGGAGTCTTCCAGGCTTGAGCGAACCGAATCCCGTCGTACGCCGGCCAGCCACCATCGACCCAAGGAACCCATGCCGGCAGGTG
CCGTCATGACACGAGCTCCGGCTCGACATCACCAAGAGGCAAAGGCACCCCCTGCACGGCCTCAGCCGACTGGGCCTTCTGCCGC
GGGTCCGATAGCCGCTGCTGCTCCGCCTCCACGTCCCAAAACATCAAAGCCCACGACCGACTTTAGTGCGCTCAAGGCTCAACTG
CAGGAGCCAATTGTTTCCAATCCCACGCCCACCTCGACGGCCCCAGAGCAGGCGGCTTTGCCCGCCGAAACCACGGCAGAAGTCT
CAACACCAACGCAGCCGCTCGCATCTGCTCCGCCCACTTCTACGGCCTCGCATGAAGCGCCGGCAAGCTCTGCCGCACCCGCTAC
TCAGCACGGCGCAGGGTCAGAGGATCACGCTACCGCTCCCGACTCGGAGCCTCCCATCACTTTGGTCCGCGCCATCACCGCCATC
ATCCCCAACGCCTATGACAATGATGCCTTGGTCTTGGAGGAAAATGATTTGGTTCACGTGCTGGATGGCGGCGAAGGCGCACTGG
AGCGGGGCCGCGTTCTCAGAACTGGACAGATCGGCTTGTATCCCTTTGCAAAGACTGAGGATATTGAGAGCCACAACTACGATGC
CGAAGTGAAGGCAGCCGTCGCTGACTACCTGAAGCGGTTTCCAGATGCATGCTAGCAAACCATAGGTGGCTATTGGCGACGGTCG
TGAGATACGT

>M_brevicollis_crka2 | KT795325 | MONBRDRAFT_25437 => unspliced (sequence data on 3
clones)
TCGCAAGCTCTCTCAAGCTCTCTCAACCAATTCGTCTCTCTTTCTATTTTTCTGAACGTGTGGGTGAGTAAGTCTCTCTCTTCAA
TTCTTTCTCTCTCTCTCTTTCTCTCTCTTTCTCTCTCTCTCTCTCTCTCTCTCTCTCTCCGTGCTCGCACACACACACACAGG
GCGGCGACGGCACTCCAGCGGTTTCCTTGGCAGCGGTGTGTATGGGCTGTGATGGACGTGAGGGCTGCTTGTGTGGGATCGTTTG
GACCAGCGCCCGGGTTGCTTTGGTTGTATCTGTGTGGGCTGGTGTTTTTTTTTTTTTTTTTTTCAGGATGGTAGCGAGCGCGTAT
GCTCACCAAGGCGGGGGGTTTGATGCTTGCTTGCCATGATGTCTGCAGGCACCTCGACTCAGCTCCTGGCCGAAATCATGGAGGA
CATCTTGACGATCCGCCGCGAGGCCTTTTCCATGGCGGGCCCTGCCCCGGCTTGGTATCATGGACAGGTCTCTCGCACAGACGCG
GACTCACGCCTGCGCGGCACTCCGGAAGGTACCTTTATCATCCGTGATAGCTCCAAGGGCGGCTCCTTTGTCCTGTCCGTCAGCG
AGGGTGGCAAGGTCAGCCACTACAAGATTGACCGCCGCAGCCCTTGCGTCTACGAAATTGGTGACGAGTCTTTTGCTAACCTGCC
GGAGATTATCGAGTTTTACAAGCGCCATATGCTCGACACCACCCCGCTCTCCGTGCCTTTGCCCCTTGAGGGCGAACTTGTGGGG
GCAGAACTTGCCGCCAACTACCTCACCGAGGCCACCGCCCTCTACAACTTTAATGCCCGTGACCCGGAAGATCTCAGCTTCCGCA
AGGGCGAGCAGCTCTTTGTTCTCGAAGTGACCGAACGCGAGTGGTGGAAGGCCCAGTCCCGCGCCACCCGCCAGATCGGCATGGT
CCCCGCCAACTACCTCAAGACGGCCAAAAACATCTTGCCAGACTTACCTGCTGATTCCGCCCCCGAAGCCCCTGCACCCGAAGCC
ATGGCCCCTGCTGCCATTGATCCCACTCCACCCCCGCTCGCCAGCCGTGGCCCCCTGCCTCCGCCTCCGGCCGAAACTCTCGCTG
CAATCGACCCCACCCCTGTCGTCACCCCGAGCTTACCCTCCCGCGAGCCCAATAACGCCCCGGCCCGTGCCCCCGCCTCATCCAG
CGTGGTTCCATCTTACTTCTACGAGCTCTCCAAAGACAAACTGCCCGACCGCCCAAACTTTGTTGTCTGCCGCGCCAAACTCGAT
CGCAATGCTAAGAGCTTTGACAAGAGTGCCCTCAGCTTCAAGGTCGGTGACAATCCCCTAACCCAAAACACCCCCTGCCTCCCAC
CACGAAATTATTATTACTTTTTTTTTTTTTTTTTCATCGAGTGTGGCTTCCGGGCACCTCATGGACGCTCTGCGGGGCCATTTTT
GAACGCCCTGATGGTGAGTCTGCTCTCACCTGCACCTTTCTTGCCTTCTCGCGTATACCTTGTGTCGACAGGCCGGGGATTTGAT
TCACGTCCTGCATCAGCCCAAAAACGGCTTGTGGCGCGGTAAACTGGTCAATGGTGACTCTGGCAAGGTTGGTCCGCCAGTCCGT
CCGAGCCCCTGAAACAGCTGCCAGCCCCTGCGACTGGACGTCCAGGCCCCAGTTTCCTCGCCCTCAGTAGTGAGTCGCTGCGTTC
TCACGGCATCATATACTTTGAATCACCTGCACAGGTTGGCCATTTTCCTTTCACACTTGTTGAACTGGTCGACGCCGGTGTCTAT
GATGATGAGCTCAATGCCATGAAGAAGGTTTTCGTTGAGGAAATGGATGCCATCTACGGCACGGCATAGAATGAGATTCCTTCT

>S_kowalevskii_crka | KT795326 | G613P6149RE5.T0-complete
GCCATCTTGGATTAGTTTACTTCCTTGAACTTTCGATGTGAGTCATCTTACTGCGTATACCGTCAATTGTTGAATTATTGTACAA
CGTAGGAATAATTTTACACCGTGATTGGCGACTACTGATGGTAAATGTGGTGATCGGGCTCAGTTGGAAGGTTGTTCATCAAACG
AAGGGCGGTTCTGTTTCATTGGACCTATTTTGACGTGAAACCTCCCAATCAGAATTCCAAGGAAATACACGTTTGGGACGAACCG
TTTGCAAAGTGGGTGTTGTTGCCTCACGTTTGCGATATGGCAGGAATGTTCGATTCGCAAGATAGAAATTCGTGGTATTTTAGAG
CATTGTCCCGCACTGAAGCTCAGCGATACCTCCTGGGTACCAGACATGGACGATTTTTGGTGCGGGATAGCAAGACATGCCCAGG
AGACTATGTGTTGTCAGTCAGTGAAAATGCGAAAGTGTCACATTATATAATCAACAAATTACAATCACAGAAATATAAAATTGGA
```

GATCAAGTGTTTGATGACTTACCAGCATTGATTGAATTTTATAAAATCCATTATTTAGATACAACAACGCTTATTGAGCCGTGTC
CGCGGGAACTCTCACCACAGCAGCCACAACCACCTCTGCTGCCACCTCCAGTAGCACCACCAACCCAACCACAAGATGACAAATA
TTACAAGGCTTTGTTTGAGTTCCTAGGAAGGGATCCAGACGACTTGCCGTTTAAAAAAAATGACATTCTATTATTGCTGAACAAA
GATGAAGAAAATTGGTGGACCATGAAGAGCCTGACATCAAACAGGACTGGGTCAGTCCCTGTGCCATACATAGAAGAGTGTGCAA
ATCCTAATGAAGTCATATATAGGCCAATTGTCCCTCATAATGGACCTGTCAAGGCAGTTGTTACACAGCAAAGAATACCCAATGC
TTATGACCCCCAGGCCCTTAAGTTGGAGGTTGGAGCTATACTGACGGTGCTTGAACAGAATATCAATGGTCAATGGAGGGGAGAG
TTGAATGGTAAAGTTGGCACTTTCCCATTCACACATGTCAGGATCTTACAAGACAGTGATATGGATAGTTAGAACTAAGACGAAC
GCAAAAATACAGAGATATTGGACTCTTGTAGTATTAATGTAGTCTGACTGCATTTCTCCAGGCACTGGATGAGACAACCTGATAG
TTGTAAATTGTCTGTAAATATGCTGTGAATACACTCTGTAATGTGATCTTAAGATAGTTAATGGTAATGCTGTTTGCAGTACCTG
TTGTGGGTTTTCATGTGATGTTGGCAGTGTTCTCTCCAGATTTTCATTGACACAGGTGAATGCAGACCACATTTATGTAAATGTA
AATGTTAATGGTTCACAGTTTATAACAACATTGGCACCTTGAGATTACCATGAATGCATTCATTGCTAGTGAAACGTAGAGGTAT
TTATGTATCAACTCCAAATGTTCAACAAGCTGATGACAGAAGGCAAGTCACACTGTGAACCTTCACAAGACAAGACCAGTTCAAG
GCATCAAATAAATTGGGAAATATATATACATCAGTCAGAAACTATTGAACTTTGAATTAATTCTAGAATGTAGCATCAACTTGAT
AACCTTGGCCGGTATGATGTATAACATTAAACGCACTGGAAAACAAGGATGCAGCAGTAAATGTTGTGTAACATGCTTAACACTA
ATCACATTTACTTACTATGACTATGTATACTGCCTGTCAAAATATGTGTAACCTCAAATTATTTGATGGAAAAAATGATACAACT
CCTACTACAGGCATTTTCTTATCAATTTAACGCTACTTCACATTTGCATTGCAGTTTGTGTAATGAATTTACATGGATCGTTTTT
TTTTTTTGCGGGGGGGGGGGGGGGTAAGGTATATTGAAACAGCTTTTAATATAATGTTACAATTTTTTTAAAGAACTATTAATT
ATTTTATTAATACTAAACTGAGTATCTAAACTAAAAACGAAGCACATTAGTCACTTTTTTTTCTCTGGGGAGAACACTGCTTTTG
CACAAATCTAATCTAATAGTGAATTGGAATGTTACTTGTCTTTACATTGTCTGTGTGGTTTTCTATTCAACTTATCAGAGTCAAT
TTCAGTCAGTAATCATTGTTTTCTTGTATGCAACTTAGAGTTAGGGGTGACTTGTTGTATTTTCACTCCGTGAGTGAGGGACAAT
CTGTAACATCTGTATGTCTTGATTGTCCATGGTTGAAATGTGTAAATACTGAATGGGCTGTTTATCAAAATCAATTTTACAGTTT
CTGAACAAGTCCTAAATTATACTTCATAATTTATCCTCTTGCAGAAGTTTCACATTTCAGTTTGGATATTTTTAGTTTTGGTTCA
GAAGAAAAGAAGACAAGTTTCTGGTCATTGAATATGATCTTTGCATGGAAACTTATTTTTCTTTTGTTTCTTTGAAAATTTTGAC
AAAAATCTTGTAAAACAATGTGACTATTAACTATTACATGTAAACCCCATAATCAGAAATATTTGAGTATTAATTCACGTTAAAT
ATCTCATGTTTCAAGCATATGAAAACTCTTTGGAAATCACACATTTTATTAATCACAGTGACTTTTAAAATCTTGTCAGTTTATG
CAGATAAGTCATATAAGAACTATGGTTTGCTCTACTTTTAACCATCTTGAATGTTTAAAACAAAGTGACAAATTGCACTACTAAA
GAGTTAATCAATTTCTATTACGGCTCTAGATTTCTTAGTAAAATATTTTTCTTGTAGAACACAAACACAAAAATTAGTGTGGATA
ATTGCTAGAACACTTGATACATAAATTCAACAAGCTAAAGAAAGATAATTTGAAGTTACAAACTAATAGTCAATATACTGGTAGG
GATTGGGTAAATAGTAACTCATAGTACACCTATCAACAAATTTTTTTTAGTGTTTTTTATTTTAAAACTACATTCATATAAGATT
AATTTATCCTCATCCAGAAATATGGAAATTGTTATATAAAAGTTATTTTTTTGTTAAAATCTATTCTCGGTTATGTTTACTTAAC
TTTGCTCACCATTTTTTGGGAGGTTTTTTTGTTTAGTGTTTTTATTCAAAGATCTTTGTAGGTTGTCATGACCCATCCCATTGCA
TTGTGGGTAGCTCTTATAGGGAGCACTAGATTTCTGCAGGTTGGATTCAATGTCTGCCTTGTACTTAATTAAAATGAACAATAGT
ACATCCATATAATTAATTCTGTACATCTTAATGCAGTGAGTGTGACAAAACTTGCCTTGCGTTTATATAGTTTTCTCATTTATAT
ACTTGCTTAAAATACCGGTATGCAAATTTGTTTTCAAACATTGGACTTTTGGAAATGTGTTTTGTTTCATGAGTGACACATTCTA
ATTAAAATCAAAATGTAGGTACTTAGTCAGTTGCCACCCACCCACATCTACACCTGACCAATTATCTATGCAAACCATGTTGTTC
TGGGAAGGGCTTTGTTTAGATTGATGGACATCTTACCATTTTGGGTTACATAAGTGAAATAAAAATGTAGTAAATTTCAAATAAA
TTATAGAAATGTAATTCATTTAATTCAAGTGAAAAAATAAAAATCGACAAGAATAGAGAATGCTGCGACTGCACTAGTGCAAAAT
CACCATTCTCTATAGATGTACAATGTTAAGTGCAGGTTGTTTTGTTTGCTTTGGTTATTTCTCAGTTATATCTGCAATAGTTAAG
AGGCATATAGCGTGTAACTGTGATCATATCAGCAAGGTATTGATCTACCTGGAACAATCATGGACTGCCTGTCACTCTGGAAAAT
TGTAACCAGAATGCTGATAGGCTGTTTATTATGAGATCTTGAAAACTGTAACCAGAGTGCTGATTGGTTGATTATGATATCTGAA
GAAAATTAGTATGGGTATTTGGTCAGTTATAGATGCATTCTTTACCACATTTTTATTTCAACCTTTTCACCACCAGACTTTTTCG
CCCAAACTCCAACAGATAAAATTTAAGACAACTTTTAGTTTTTACTCGAAATAATTTGGCTATTTTATGTCTCAATCTTCTGTGA
ATTTATCATGAAAAGATGGAACTGGTGTTGAAAAGGTTAAAATCAACCAGAGTCGCCTCCTGATTTTATTCAGATTGTGAGTAAT
CATTCTCATCTTTAAAATTGCTGAATTTCACTTTATCTGTAGCTGAGGTATTTCTGTTAACTGTTTTGACAATATACCCATTTTG
ACATTTTTGTAAATTTTAAATTTTGGTTGTTTCCAAACATTTGATTTATTGATATTTACTTAAAAAACTAATTTTATTTTATGGG
TTTTTAATTTCTTATCACATATGTGCAGTAGCCATACAGTGAATCTGTGTCATTTCTATGTTGATTTTAATTTCATTATGGTTGG
AATCATTGCTTGTAATTATGTTTATAATGTTACCCTGTATGTCATGTGTTTCTTACCAGATGTATTCAGCTTTATAGAATATACC
GGTAATATAAAACAATGTCCAATATGGAAAATCCAAACAGGTACATGTACAGCGTATCCATTGGTAAACATATGCAATGTATACA
TTAGTAATTATGCTATACACAAAAGCAAAATCTTTGGCAGTGAAGCTACCGGTACTTGCGATATGACCCTACAATTGTGATGCTG
CTATCCTCATTAATTCTGCTGTTTACTGTTCAAAGTTCAAGCATAGTGACAGTAGTGGCAACAATTTTATCATTGATGTTGATTT
CACTTTTTTAAAGAGGATTTTTTTATTGTAAAAGTATCTCTACAAATATGTCAAGTAAGCAATGTGTGTAGCCTACTATAAATAA
TTTTTCAATGGGATGAGATTTTGTTTATGAGCCACTTAAAATAAATTTATTGATTACCGGTACTAAAACTTGAGAATGTCCAAAT
TTTCTACATATTAATCATGCATACAGTCTATGCCATATCCTTATATAGTTATGGTATTACATTTATAAGTGCAAATTATGGTAAA
AAAAATGTTAAATGTTTATGCCTTGTAATTAAGTACAGACAACTCTACAGCCAGACACTCAGTAGTGTAGATGGCTGAAGTAATT
CAATTTAATCAATATTAATGTATTTCTTTTCTCATCAATATTGTACATTTCATCTAAAACTTAACTTACTAAATCTTTGATTTGC
ATAGTTTGTATTATTATTTCATTATATCAACTTCATTCTTTGTATGGATTTCTTTGCTCAGTCTTCATCTTCCATATAGTCAATT
TAATATAATAAAAAAAAA

>B_floridae_crka1 | KT795327 | bflv061c08 | Scaffold_202_Bf_v1.0
TTTGTGTGAGTCGGACTTCCTCATAATTTTGTTTCTCCGCACACCGCCCGCATATTCCCGCTATTTCCTGCCGAAAGGTGCCTTC
TTATTCATCCTTCCTTGACGGAAACTTCAAGAATTACTTCAGAGGGATACAAGCGGGTCAAGTTTGCCGAAATTCGGAGGGAATT
GGTAGAAAAACTCGAAGGGGGAGGTGTGCAAACAGAGAAAAGTTCAGTCGGCCAAGATGGCAGCAAACTTCGACTCCAGAGATAA
GAATCAGTGGTACTTTGGCGGGATAACTCGGGACGAAACGGAACGTTTGCTGTTGGGAAGGATGCACGGGTTGTTCCTGGTCAGG

GACAGCAGAACTAGACCCGGGGATTACGTCTTGTCGGTGAGTGAAAATAACAGAGTCAGTCACTACATCATCAACAAGCAGCCAA
ACGGTTTCGAAATCGGCGACCAGGATTTCGTGGACCTCCCGTCTCTCCTGGACTTCTACAAGATTCACTACCTGGACACGACCAC
GTTGGTGGAACCCTGTCCGAAGAACGCACCGGCCCCGCCCCAGATCAACACGCCCCCTCCCATCATCCCTCAGCAACCCCTGTAC
GAGAGGACGACGCCACAGCAGGAACAGGTACGCGGCATCTTCGACTTCCCCGGAAACGATCCCGAAGACCTGCCGTTCAAAAAGG
GCGACATTCTTACCATAATCAGAAAGGACGAGGAGGAGTGGTGGATGGCCCGCAACTCGACTGGACAGGAGGGTCAGATCCCCCG
GCCGTACGTCGAGCGGTACACCCCTACGAGCGGAACGGTGCGCTACGGACAGCTGAACATTCCCGTGAAGGCTCGCGTGATCCAG
CAGAGGATACCTAACGCGTACGACCAGACTGCGCTGAAGCTCGAGGTCGGAGACATTGTCACGGTCACGAAAAGAAACCTTAACG
GCCAATGGGAGGGGGAACTTGACGGCAAGGTTGGACACTTCCCCTTCACGCACGTGAAAGTCATTGACCCAAACGACCCAGACGA
CAACGAGGTACCGTCGTGAAAAACACGGACAGTTAGTCAGAAACTAAATATCAAAAGTAACTGCCACTCTCCTGACTAAGCGTCA
TGCATTTCTATTAGTAATCTACTGGTACGACTGAATTGAAAATTGCAGCAAATTTTGTATTATTAATACATCAGTCATATAATTT
TATTCATATCAATAATACTGTGAATTGAATCATTTGCAGATAACTTGTACATATGTGTTATGTAAGCCTTTTCATGATTATTTCA
AATACTGTAATATCTGATTCAAGAAAAATTGTAATATTCCACAGGTTACTTAATTCCATACCTTTATCTTATGTAAAATTGTATG
AGAAAAGAGGTAATATTTCTGTATAATTGGAATTATTATATAAGGATTTTTTTGCTACTATGAAGTATATAAAAATGTATTCTTG
GACACACTGAAAAAAAAAAAAAAAAAAA

>B_floridae_crka2 | KT795328 | bflv57k18 | Scaffold_131_Bf_v1.0
GGACTTGTCGGAACTTCCTGCTGAAAATCATGGCAAATTTCACGTTTTCTGAGACACCTTTCCCACCTTCTTCCACTCACACCGA
CTCTACAGACGCCTGAGGCCGCGTGGGAGCCGTATAGACGCCGTGTTCCTGCGATGTCCGTAGCGACAAGGGTTAGCGCGTAGGA
CTAGCCGTTGAGAGCAGAAACCAATTACGATGGCAGCATCCAACTTCAACCCCCGTGACATGGGACAATGGTACTTTGGGCCTAT
TCCCCGTGTTCCCACACAGGACTTACTGCAGGGGAAGATGCACGGGACGTTCCTGGTACGCGACAGTTCCAGCTGTCCCGGGGAC
TTCGTGCTCTCTGTCAGTGAAAACAGCAAAGTCAGCCACTATATCATCAACAGACTGCAGAAGGGCTTCCGGATTGGTGACCAGG
ATTTCGATGACCTGCCGGCTTTGATCGAGTTCTACAAGATACACTACCTTGACACGACCACGTTGATTGTTCCAGCGCAGCGGCC
GGCTGGAGGACCGCCAGAACCCCAGGCCCAGGCTCTAGCTCCCGCACATACTCCCAGTGTAATGGAGGAACGCGTTCGAGCGCTC
TACGATTTTGACGGAAACGATCCAGAGGACTTGCCCTTCAAGAAAAACGAAATACTGGTTGTCGTTAGAAAGGATGAAGACCAGT
GGTGGACTGTACGCAACAAGGACGGCAGAGAGGGTTCGATTCCCGTTCCGTACGTCCAGGTACTCCCACCTGGTCCAACCACTAC
AGGCGCTGCCCCATCTCATCCCCAGCAGCCCCACTCCCCACCCGGCTACCATGACCCACAGGCAGCTGGCATAAGAAGTGAATCA
GGATCGTCTTCTACAGCATCTGCCACATCAGCCTCCACACCCCACCCCCTGCCCAACCCACAGAACGGCCCAGTGTACGCGCGCG
TCATACAACAGAAAATCCCCAGTCCTTACGACACAACGGCATTGACGCTAGAAGTAGGCGACATAATCCGAGTAACCAAGATCAA
TGCCAGTGGGCAGTGGGAGGGAGAACTCAAGGGGAAAACGGGACATTTCCCCTTCAATCATGTTAAGATTATCGACCCTAGTAAT
CCTGAGGAAAATGATTCATCTTGGAGCTAAGCAGCACTGGTAATAGATTAAAATATACATAGAAAAGGCAAAAACAAACAAAGAA
GAAACTTGGAGGCTAACATGATGTTAAAACGTTCCATGCAAAAAAAAGTGACAGGTAAAAAGTTGGACTAACAAACTGCCAAGCG
AAGAAAGGTGGAGGGTAGGATTTTATTTTTTGTGTACATGTACTAATTGGTTCTGTGAAAAGAAAAAAAGGTAAGATACTGTGCA
GAATTGAATGAGAATAGCTTAGTCGTAAATGTAGTTAGGACGGTAAATTGGGAGAGAGTAGAGACAGATGTTTTAGAATGTAGTT
GAGAAGCCAGTGCAGCAAAGTTTAGTGGTTCATTGTGCAGATCTACCACAATCACATAGTCAAAGCTGCCTTGCTACAGCAGGTT
TTACTACAAGGCAGCATAACTACATGTACACTGTTCTACTGGTACACAAGGTTCCCTTTCTTTTGGAAGGAAATCTTGGCAATTG
TTAGAAATATACACTATTTTCAATGGTGGTTAATTACCCAGATACATGTACCTGCAATGGGTGTTAAACATCTCAGTTATAAATC
CTCTACAATCTACAGTGAGATAGCCACATGATAAAATATTGGAAATGCACTGGCAGAGCCCATGTTAAAAACTTTTTTAGGTCTG
TGTTTTATATGCATATCCTTTTTGAAAATTTTGCATTTTCTTCTACCTTACTATCTGAGATGGATGGCATATGCTGAATGAATGG
GATCTTATTTTGACCTAAATGTATGGAAAAGAATATAATGCAGTGGACTTGAAAACCAGGAATGTTAATGAAGTGGAATTTTGGC
AAGATAAACTTACCATGGGATCTGCACTTGACTTTTGTGACATAGGTGAAAACAGTTTTTGCTCTTGTCCTGCCATTGGGATTAT
GTATTGTAATAAATTTACAAGCGGTATCATATCATGCATTAGTTCCTGATGGTTTACGTTTTGTTGTGGTACAGATATACCACAA
ATTGTATAAGGTCATCTTTTGTAAGTTGAGAACAAATTTTGTTTTCTGCATTTCACTTTCTGCAAAAGTTATTTTTGATCATACT
TTCCAAAAGCTCCCTGCTGAAAGATGAGTAGAAATTATGTACATTTATGTTTTGATAATGTTGTGTCCAAACTTCTGTATTGATA
TATTATGTTAAATAATATTGTTGACTTGATTGTAAGAAAGTGTTAGCAATTATATGATATCTAAAATTTGCATTATACTTGTGTT
ACTGATATGAACAAAAGCCTCCTGGCACTTGTATTATAGCCAGCTCCTATGTAGACTAGCTAACTCATATTTGTTTTACGTACAC
CTGAAATTTCCTTTAAATCACCAGGTACAGGTCTCAGTAGATGGTAGAGTATTGTGCTGTCATTTTGCATATGTGATAACATGTA
TATCTGAGACAGATATAAAGTAACTATTGAGTATATTGGTAAAAAAATAATGTACTTTTTGTAATTTCTATTGAATTTTGTTTCA
CAGTTTTGAAAAGAACAGCTTTTGTCACAATGGTCCTAATAGGTAGCATATTATTTCTCAATGCAATTCAAACATAACATTGTGA
AAGCATTCACTGAACACAGCCTGCCTCAAACGGTGTATTTTGTAAAGCCTAAAGACTAGTTGGATTTTGTCATGTCATGCTGTAA
TACAACTCCAGAACAGTTGCTTACCACTCAACACCAGTCGCTTCAAAGTAGGACTGACAGTGCAAAATTTGAAGTTGCAATTTTT
ACATCCAACATAAACATGGCTATGGCAGAAACAAAGGTACCATTAAGCAAGTGAAGGCATGCTCTTTGAATTGATGTGCATCTCT
GGATTTAAAAAAAAAAAATTTGAAAATAATTCCCAAGCTCTTCAACATCACCAAGTTTTTTATTTCAATTGGAAGTGGCCAGTGTT
ATACCATTGTAAGGCTGAAGATTATGTCTTCACTACTATTTATACACTCCAGAGGTAAAAAAAGAAGATTGTTAGAAAAGCATAA
TATGAGGCATGATTGGCAGAGAATTATGATGGCACTGAGATGGCTGTCTTGGCCTTCCACCCTTCATTTTGGCTAGCCATGCAAT
GAATGATTGCATGGTGACGGCACCAGGTGACTGGGCACCAGGGATTTTCAAAGTCTTTGGCAAGTTTACGAGTTGAATAGAATTT
GGTCTGAAAGAAGTTTGACTACAAGGTGGCCAGGCCATGTCCCTTTTACATGAGAACGCTGCATGGTATCTTTCCTGCGTCTTAA
ATCATGTACATTACATGTTTGTGCATTTTGCCTAGTTCAAGAATTGCACGGTGCTACAGTGTGAGCTGGCAGTGTATAATGTCAA
GTGTACTAGTCATTGTAATTCATTATGGCTCTGTGGTCTTACACTGCTATAGTTGTTAATTATACACTGCTGTCATGTCACTGGT
CGCTATCAGTAAGGACAGACACTGTTATTTAGGAAATGTAATCCCCTGCTTCAGCTTGCTACTGCATAATGTAAGGCTTGACATG
TCAGTACGTGTAGGTCCATCACCATTTTGTGGATAAACATATTGACTTTGGTGTTATTGTGTACAAAACTAGTATTCTTCTTCAT
TGATACAAGATACTGGACTCAAATCACACTGAAATATGCTCCAACCACAGTAAATAATTTGCAATGTGATACAGTTTTTAAAGTG
ACATTGCAGAATGCTAAAACTGTTAGAATGTTCAACTCTTGTATCAGATTACTACCAATTATGGCAGATAATGGCAATCACCTTT
TACTTAAGTTTTAATATGATACATGTAATATCATGGGTCATGACATCTCTTACACTGAGAAACAAAAGTAAGCACTGTCGTCATG

ATGTTCCAGGCACAGAATGCAAAACATTTAGTGCTCATCTCATCAAAGACAAGGCAAAATACAGGCCATGTTTGAAACTGGTAGA
AATGCTATCACCCAAGAGTTGGGATATGTTTGCATACAAAGTTTTCAATCAATTTTCAATCAAAGTGATTGGCATTTAGGTGACA
TTGAGAAATGCACAGAGTTTGTATTACAATAGGTTGCCTTTGTAAAATTCAAAGCAGGAACAAAGTTGATACTGGGTTAGAAGAT
TCCTTAACATTTTGTGGAATGTTGTTCAATACTTGATGAAATACCAGTTGTTTCACAGTAATGCTGTAATGCGCAGAGCTTGTCT
GTATGTAGACATGATTACATACTCATAGATGTGACAGTTGCAACAGTACTAAGGTATTCAAAGACTTCATCTGTGATAGTAAAAA
GCCGTAGTGTTACAAGGTAATACACACAAAGTTTACAATGTTACATTTGAAGTTCTTGATGCATTAAAGGTTTAGGATTTCCTTT
TATGTGTCTAATATGTGATAGCTCAATATCATCCTTTTCTGTACTGTGGTCACTACAGTATGTACCTATCCTGTGAATAGATGTC
TGTTTCTTCACTCTGCTTATCTCAATGAGGTGAATGTATCACATATTCGTTACCCACCATGTTAATGAGCAAAACCAATAAAGGC
TTTTGCATGAACTGCAAAAAAAAAAAAAAAAAAA

>P_marinus_crkl | clone_3
TCCGTCCGTCGTTTTGTCGTCGTCGCCGCCGCCGCCGCTCGGTTTTCTTGTTCTCCGCCGGCGCGGGTTTCGCTCTTTCCCCTTC
CGTCGTCGGTCGCTCGCTCGGTCGCCACCACCACCACCGCCGCCACGATGGCCGGCAACTTCGACGCGCACGACCGTGATAGTTG
GTACTTCGGGCCGCTGAGCCGCGTGGAGACTCAGCGACGTCTCCAGGGCCAGCGGCACGGCACTTTCGTGGTGCGCGACTCGACC
ACGTGCCCGGGCGACTACGTGCTCTCGGTGAGCGAGAACTCCAAGGTGTCCCACTACATCGTCAACAACCTCGCCGACCGCTTCA
AGATCGGCGACCAGGAGTTCCGCGACCTGCCCGCTCTGCTCGAGTTCTACCGCATTCACTATTTGGACACCACCACGCTCATCGA
GCCCGCGGTGAAGGCCACGGGGGGTCAGGGGCAGCAGGTGCCCGCGACGCCCGCGGCTGCGGCCACGGCTCGTCACCACCTCGAC
TACCAACTTCAGGGCCAGTCGCCTTCGCCGCCAGCCTCGAAGAAACATCAGCAGGGTGGTGGCGGCGGCGGCGGCAGCGGCGGCG
GCGGCGTTGACGAGAGCGTGGAGTTTGTGCGAGCCCTCTTCGACTTCCCCGGCAACGACGAGGAGGATCTGCCCTTCAAGCGCGG
CGAGATCCTCACCATCGTCGCTAAGCCCGAGGAGCAGTGGTGGAGCGCCCGCAACAAGGAGGGTCGCACGGGAATGGTGCCCGTG
CCCTACGTGGAGAAGGCATCGCGGCCGCTTTCTTACGCCGCCTCGTCCATGGCACCGGGCAACCGCAACAGCTACGGCTCTGGCC
AGGGCGGTGACCAGGGGCCCTACGCGCACCCCCTGGCCATCGGGCAGCAGCTTCCCAACCTGCAGAACGGGCCCGTCCTCGCCAC
CGTTATCCAGAAGCGAGTGCCCAACGCCTACGACAAGACGGCGCTCGCGCTAGACATTGGTGAGGTGGTCACGGTGACGAAGATG
CACGTCAATGGGCAGTGGGAAGGGGAGGTGAATGGCCGAAGGGGCCACTTTCCCTTCACGCACGTGCGGATCATAGACCAGGCCA
ACCCGGAGAACTCCTGATGGATGCGACCCTTGCCGTCCTGGCCGTTATTGTGTTCTTTCAGCCAGACTCTAAAACCGTGACTTTC
AATGGATATTTTTTTGTTTCTAATGAAACTTAAAGGCAAGCACGAAAACACGTGGACCTAGTCAGATGTCATCTTTGGTTGAATG
TCTTTTTGCCGTGAAACCGTCGAGACCGTCCCACGTCGATGGGTGATGGACACCCATGATGCGTTCGTGTGCCCTCGGGCAGTGG
GACTCGCCGGGTAAAGGACTGCATTGGAAGAAGGAAGATGCGTTCAGAGTGCAGAACTTGTGTTCGTAGGGCTAAACATTTCTCT
TGCTGTAACTGCCATTTGTAATAATGTGGAGAGATTTAATGGTTCTTAATTATCATTATTAACGACAATCTTTTAGTTTTCTACA
ACAATTCCATTTGTTCTAAGCTTTATAAACCAAAATTACAGTCTTTGCAAGGTGAACTTTTTTTGTATAACTGATTTCTTTGGGT
TTCATAATTATTAATACTGTATCTTATTGGCAGTTTTTAACTTTTTATACCTCTGGTCAAAATGTATTTTTTAAGTGATGACTTA
ACTTGAAAGCTGACTCGTTGGTATCCTTGAGTTTTTTTTCCTTGCATTCAGTAACAACTGCAATGCGCGCATATGTGCATTAAAT
CATGCCAACAGTACTGCCGCGAATTTTTAGTGCCAGTGTGGCTGCACTGTCGTGCATTCTCCACCGCTCGCATGTACTCTCCAGG
GGTATGCGTGACAGCTTGTCCGTTACCACATTGCCAGCACACAAGCAGATATTTGAGCAGGTTAATTGGAGTGCTTTCTGTGCCT
TTAACTTATTGCTGTAAATGCAGGCTTTTCTTCTGCATTGCTGTTGATCGGGAAGACTGATGAACATTTTGACTGTCTGCAGCAT
CCGATGTGACCCTTTCGGCGGCGTATGGTACTCATTGTTGGTCTGTTCGTTTCATGTTCGTACTGACCCTGTCAGACTAGACCAA
ATAGACATAGGGGGCACAAAGATCGTTTGACCATAAGATTCTGCGGCACATGTCGCTTGCGCCACTCTAACATCGACTGTGTGCA
TATCATCCGGCTGTTTAAAGGGAACCGTCTCAGTGCTGTACCGGACCAGGTTTTTCTTTGCGAGCTGGTTGTGAGGTGTAGCTGT
CCTCTGGTAGTGCTTGTGTTTGAATGGTGGACAAGAGCCTTACAAAGCGCCATTTGCTTGTATGCCAAATCAGGTCTCTTAACTG
TTATGTACATTGTGGTAGATCATGGTTATTAATGGTACATTGTGTACATGAGTAATATAAATGCGTTCACGAGCTGCTTTTATAA
TTGGGGGCACTAAAAAGTGAAGAAAATAAACGCAAAATAAACTGGCACGTCTCAAGTTTTATAAGTTGTTTTATAAAACGCGTAG
GATTTAAGTCCATTAACAGTGACCAAATGCGGACCAAGGTGGTGGCTTCCTCAAAACAACTCTGATGGCACTTTAATATGTTGGT
GTTGCTTTCTGCGCAAGGGCGAATTCCAGCACACTGGCGGCCGTTACTAGTGGATCCGAGCTCGGTACCAAGCTTGATGCATAGC
TTGAGTATTCTATAGTGTCACCTAAATAGCTTGGCGTAATCATGGTCATAGCTGTTTCCTGTGTGAAATTGTTATCCGCTCACAA
TTCCACACAACATACGAGCCGGAAGCATAAAGTGTAAAGCCTGGGGTGCCTAATGAGTGAGCTAACTCACATTAATTGCGTTGCG
CTCACTGCCCGCTTTCCAGTCGGGAAACCTGTCGTGCCAGCTGCATTAATGAATCGGCCAACGCGCGGGGAGAGGCGGTTTGCGT
ATTGGGCGCTCTTCCGCTTCCTCGCTCACTGACTCGCTGCG

>P_marinus_crkl | KT795329 | clone_10
CCGCCGCCGCCGCTCGGTTTTCTTGTTCTCCGCCGGCGCGGGTTTCGCTCTTTCCCCTTCCGTCGTCGGTCGCTCGCTCGGTCGC
CACCACCACCACCGCCGCCACGATGGCCGGCAACTTCGACGCGCACGACCGCGATAGTTGGTACTTCGGGCCGCTGAGCCGCGTG
GAGACTCAGCGGCGTCTCCAGGGCCAGCGGCACGGCACTTTCGTGGTGCGCGACTCGACCACGTGCCCGGGCGACTACGTGCTCT
CGGTGAGCGAGAACTCCAAGGTGTCCCACTACATCGTCAACAACCTCGCCGACCGCTTCAAGATCGGCGACCAGGAGTTCCGCGG
CCTGCCCGCTCTGCTCGAGTTCTACCGCATTCACTATTTGGACACCACCACGCTCATCGAGCCCGCGGTGAAGGCCACGGGGGGT
CAGGGGCAGCAGGTGCCCGCGACGCCCGCGGCTGCGGCCACGGCTCGTCACCACCTCGACTACCAACTTCAGGGCCAGTCGCCTT
CGCCGCCAGCCTCGAAGAAACATCAGCAGGGTGGTGGCGGCGGCGGCGGCAGCGGCGGCGGCGGCGTTGACGAGAGCGTGGAGTT
TGTGCGAGCCCTCTTCGACTTCCCCGGCAACGACGAGGAGGATCTGCCCTTCAAGCGCGGCGAGATCCTCACCATCGTCGCTAAG
CCCGAGGAGCAGTGGTGGAGCGCCCGCAACAAGGAGGGTCGCACGGGAATGGTGCCCGTGCCCTACGTGGAGAAGGCGTCGCGGC
CGCTTTCTTACGCCGCCTCGTCCATGGCACCGGGCAACCGCAACAGCTACGGCTCTGGCCAGGGCGGTGACCAGGGGCCCTACGC
GCACCCCCTGGCCATCGGGCAGCAGCTTCCCAACCTGCAGAACGGGCCCGTCCTCGCCACCGTTATCCAGAAGCGAGTGCCCAAC
GCCTACGACAAGACGGCGCTCGCGCTAGACATTGGTGAGGTGGTCACGGTGACGAAGATGCACGTCAATGGGCAGTGGGAAGGGG
AGGTGAATGGCCGAAGGGGCCACTTTCCCTTCACGCACGTGCGGATCATAGACCAGGCCAACCCGGAGAACTCCTGATGGATGCG
ACCCTTGCCGTCCTGGCCGTTATTGTGTTCTTTCAGCCAGACTCTAAAACTGTGACTTTCAATGGATATTTTTTTGTTTCTAATG

AAACTTAAAGGCAAGCACGAAAACACGTGGACCTAGTCAGATGTCATCTTTGGTTGAATGTCTTTTTGCCGTGAAACCGTCGAGA
CCGTCCCACGTCGATGGGTGATGGACACCCATGATGCGTTCGTGTGCCCTCGGGCAGTGGGACTCGCCGGGTAAAGGACTGCATT
GGAAGAAGGAAGATGCGTTCAGAGTGCAGAACTTGTGTTCGTAGGGCTAAACATTTCTCTTGCTGTAACTGCCATTTGTAATAAT
GTGGAGAGATTTAATGGTTCTTAATTATCATTATTAACGACAATCTTTTAGTTTTCTACAACAATTCCATTTGTTCTAAGCTTTA
TAAACCAAAATTACAGTCTTTGCAAGGTGAACTTTTTTTGTATAACTGATTTCTTTGGGTTTCATAATTATTAATACTGTATCTT
ATTGGCAGTTTTTAACTTTTTATACCTCTGGTCAAAATGTATTTTTTAAGTGATGACTTAACTTGAAAGCTGACTCGTTGGTATC
CTTGAGTTTTTTTCCTTGCATTCAGTAACAACTGCAATGCGCGCATATGTGCATCAAATCATGCCAACAGTACTGCCGCGAATTT
TTAGTGCCAGTGTGGCTGCACTGTCGTGCATTCTCCACCGCTCGCATGTACTCTCCAGGGGTATGCGTGACAGCTTGTCCGTTAC
CACATTGCCAGCACACAAGCAGATATTTGAGCAGGTTAATTGGAGTGCTTTCTGTGCCTTTAACTTATTGCTGTAAATGCAGGCT
TTTCTTCTGCATTGCTGTTGATCGGGAAGACTGATGAACATTTTGACTGTCTGCAGCATCCGATGTGACCCTTTCGGCGGCGTAT
GGTACTCATTGTTGGTCTGTTCGTTTCATGTTCGTACTGACCCTGTCAGACTAGACCAAATAGACATAGGGGGCACAAAGATCGT
TTGACCATAAGATTCTGCGGCACATGTCGCTTGCGCCACTCTAACATCGACTGTGTGCATATCATCCGGCTGTTTAAAGGGAACC
GTCTCAGTGCTGTACCGGACCAGGTTTTTCTTTGCGAGCTGGTTGTGAGGTGTAGCTGTCCTCTGGTAGTGCTTGTGTTTGAATG
GTGGACAAGAGCCTTACAAAGCGCCATTTGCTTGTATGCCAAATCAGGTCTCTTAACTGTTATGTACATTGTGGTAGATCATGGT
TATTAATGGTACATTGTGTACATGAGTAATATAAATGCATTCACGAGCTGCTTTTATAATTGGGGGCACTAAAAAGTGAAGAAAA
TAAATGCAAAATAAACTGGCACGTCCCAAGTTTTATAAGTTGTTTTATAAAACGCGTAGGATTTAAGTCCATTAACAGTGACCAA
ATGCGGACCAAGGTGGTGGCTTCCTCAAAACAACT

>P_marinus_crkl | clone_20
TCCGTCCGTCGTTTTGTCGTCGTCGCCGCCGCCGCTCGGTTTTCTTGTTCTCCGCCGGCGCGGGTTTCGATCTTTCCCCTTCCGT
CGTCGGTCGCTCGCTCGCCACCACCACCGCCGCCACGATGGCCGGCAACTTCGACGCGCACGACCGCGATAGTTGGTACTTCGGG
CCGCTGAGCCGCGTGGAGACTCAGCGACGTCTCCAGGGCCAGCGGCACGGCACCTTCGTGGTGCGCGACTCGACCACGTGCCCGG
GCGACTACGTGCTCTCGGTGAGCGAGAACTCCAAGGTGTCCCACTACATCGTCAACAACCTCGCCGACCGCTTCAAGATCGGCGA
CCAGGAGTTCCGCGACCTGCCCGCTCTGCTCGAGTTCTACCGCATTCACTATTTGGACACCACCACGCTCATCGAGCCCGCGGTG
AAGGCCACGGGGGGTCAGGGGCAGCAGGTGCCCGCGACGCCCGCGGCTGCGGCCACGGCTCGTCACCACCTCGACTACCAACTTC
AGGGCCAGTCGCCTTCGCCGCCAGCCTCGAAGAAACATCAGCAGGGTGGTGGCGGCGGCGGTAGCGGCGGCGGCGGCGTTGACGA
GAGCGTGGAGTTTGTGCGAGCCCTCTTCGACTTCCCCGGCAACGACGAGGAGGATCTGCCCTTCAAGCGCGGCGAGATCCTCACC
ATCGTCGCTAAGCCCGAGGAGCAGTGGTGGAGCGCCCGCAACAAGGAGGGTCGCACGGGAATGGTGCCCGTGCCCTACGTGGAGA
AGGCATCGCGGCCGCTTTCTTACGCCGCCTCGTCCATGGCACCGGGCAACCGCAACAGCTACGGCTCTGGCCAGGGCGGTGACCA
GGGGCCCTACGCGCACCCCCTGGCCATCGGGCAGCAGCTTCCCAACCTGCAGAACGGGCCCGTCCTCGCCACCGTTATCCAGAAG
CGAGTGCCCAACGCCTACGACAAGACGGCGCTCGCGCTAGATATTGGTGAGGTGGTCACGGTGACGAAGATGCACGTCAATGGGC
AGTGGGAAGGGGAGGTGAATGGCCGAAGGGGCCACTTTCCCTTCACGCACGTGCGGATCATAGACCAGGCCAACCCGGAGAACTC
CTGATGGATGCGACCCTTGCCGTCCTGGCCGTTATTGTGTTCTTTCAGCCAGACTCTAAAACTGTGACTTTCAATGGATATTTTT
TTGTTTCTAATGAAACTTAAAGGCAAGCACGAAAACACGTGGACCTAGTCAGATGTCATCTTTGGTTGAATGTCTTTTTGCCGTG
AAACCGTCGAGACCGTCCCACGTCGATGGGTGATGGACACCCATGATGCGTTCGTGTGCCCTCGGGCAGTGGGACTCGCCGGGTA
AAGGACTGCATTGGAAGAAGGAAGATGCGTTCAGAGTGCAGAACTTGTGTCGTAGGGCTAAACATTTCTCTTGCTGTAACTGCCA
TTTGTAATAATGTGGAGAGATTTAATGGTTCTTAATTATCATTATTAACGACAATCTTTTAGTTTTCTACAACAATTCCATTTGT
TCTAAGCTTTATAAACCAAAATTACAGTCTTTGCAAGGTGAACTTTTTTTGTATAACTGATTTCTTTGGGTTTCATAATTATTAA
TACTGTATCTTATTGGCAGTTTTTAACTTTTTATACCTCTGGTCAAAATGTATTTTTTAAGTGATGACTTAACTTGAAAGCTGAC
TCGTTGGTATCCTTGAGTTTTTTTCCTTGCATTCAGTAACAACTGCAATGCGCGCATATGTGCATTAAATCATGCCAACAGTACT
GCCGCGAATTTTTAGTGCCAGTGTGGCTGCACTGTCGTGCATTCTCCACCGCTCGCATGTACTCTCCAGGGGTATGCGTGACAGC
TTGTCCGTTACCACATTGCCAGCACACAAGCAGATATTTGAGCAGGTTAATTGGAGTGCTTTCTGTGCCTTTAACTTATTGCTGT
AAATGCAGGCTTTTCTTCTGCATTGCTGTTGATCGGGAAGACTGATGAACATTTTGACTGTCTGCAGCATCCGATGTGACCCTTT
CGGCGGCGTATGGTACTCATTGTTGGTCTGTTCGTTTCATGTTCGTACTGACCCTGTCAGACTAGACCAAATAGACATAGGGGGC
ACAAAGATCGTTTGACCATAAGATTCTGCGGCACATGTCGCTTGCGCCACTCTAACATCGACTGTGTGCATATCATCCGGCTGTT
TAAAGGGAACCGTCTCAGTGCTGTACCGGACCAGGTTTTTCTTTGCGAGCTGGTTGTGAGGTGTAGCTGTCCTCTGGTAGTGCTT
GTGTTTGAATGGTGGACAAGAGCCTTACAAAGCGCCATTTGCTTGTATGCCAAATCAGGTCTCTTAACTGTTATGTACATTGTGG
TAGATCATGGTTATTAATGGTACATTGTGTACATGAGTAATATAAATGCATTCACGAGCTGCTTTTATAATTGGGGGCACTAAAA
AGTGAAGAAAATAAATGCAAAATAAACTGGCACGTCCCAAGTTTTATAAGTTGTTTTATAAAACGCGTAGGATTTAAGTCCATTA
ACAGTGACCAAATGCGG

>P_marinus_crkl | clone_31
TGATCTCCGTCCGTCGTTTTGTCGTCGTCGCCGCCGCCGCTCGGTTTTCTTGTTCTCCGCCGGCGCGGGTTTCGATCTTTCCCCT
TCCGTCGTCGGTCGCTCGCTCGCCACCACCACCGCCGCCACGATGGCCGGCAACTTCGACGCGCACGACCGCGATAGTTGGTACT
TCGGGCCGCTGAGCCGCGTGGAGACTCAGCGACGTCTCCAGGGCCAGCGGCACGGCACCTTCGTGGTGCGCGACTCGACCACGTG
CCCGGGCGACTACGTGCTCTCGGTGAGCGAGAACTCCAAGGTGTCCCACTACATCGTCAACAACCTCGCCGACCGCTTCAAGATC
GGCGACCAGGAGTTCCGCGACCTGCCCGCTCTGCTCGAGTTCTACCGCATTCACTATTTGGACACCACCACGCTCATCGAGCCCG
CGGTGAAGGCCACGGGGGGTCAGGGGCAGCAGGTGCCCGCGACGCCCGCGGCTGCGGCCACGGCTCGTCACCACCTCGACTACCA
ACTTCAGGGCCAGTCGCCTTCGCCGCCAGCCTCGAAGAAACATCAGCAGGGTGGTGGCGGCGGCGGTAGCGGCGGCGGCGGCGTT
GACGAGAGCGTGGAGTTTGTGCGAGCCCTCTTCGACTTCCCCGGCAACGACGAGGAGGATCTGCCCTTCAAGCGCGGCGAGATCC
TCACCATCGTCGCTAAGCCCGAGGAGCAGTGGTGGAGCGCCCGCAACAAGGAGGGTCGCACGGGAATGGTGCCCGTGCCCTACGT
GGAGAAGGCATCGCGGCCGCTTTCTTACGCCGCCTCGTCCATGGCACCGGGCAACCGCAACAGCTACGGCTCTGGCCAGGGCGGT
GACCAGGGGCCCTACGCGCACCCCCTGGCCATCGGGCAGCAGCTTCCCAACCTGCAGAACGGGCCCGTCCTCGCCACCGTTATCC

AGAAGCGAGTGCCCAACGCCTACGACAAGACGGCGCTCGCGCTAGATATTGGTGAGGTGGTCACGGTGACGAAGATGCACGTCAA
TGGGCAGTGGGAAGGGGAGGTGAATGGCCGAAGGGGCCACTTTCCCTTCACGCACGTGCGGATCATAGACCAGGCCAACCCGGAG
AACTCCTGATGGATGCGACCCTTGCCGTCCTGGCCGTTATTGTGTTCTTTCAGCCAGACTCTAAAACTGTGACTTTCAACGGATA
TTTTTTTGTTTCTAATGAAACTTAAAGGCAAGCACGAAAACACGTGGACCTAGTCAGATGTCATCTTTGGTTGAATGTCTTTTTG
CCGTGAAACCGTCGAGACCGTCCCACGTCGATGGGTGATGGACACCCATGATGCGTTCGTGTGCCCTCGGGCAGTGGGACTCGCC
GGGTAAAGGACTGCATTGGAAGAAGGAAGATGCGTTCAGAGTGCAGAACTTGTGTCCGTAGGGCTAAACATTTCTCTTGCTGTAA
CTGCCATTTGTAATAATGTGGAGAGATTTAATGGTTCTTAATTATCATTATTAACGACAATCTTTTAGTTTTCTACAACAATTCC
ATTTGTTCTAAGCTTTATAAACCAAAATTACAGTCTTTGCAAGGTGAACTTTTTTTGTGTAACTGATTTCTTTGGGTTTCATAAT
TATTAATACTGTATCTTATTGGCAGTTTTTAACTTTTTATACCTCTGGTCAAAATGTATTTTTTAAGTGATGACTTAACTTGAAA
GCTGACTCGTTGGTATCCTTGAGTTTTTTTCCTTGCATTCAGTAACAACTGCAATGCGCGCATATGTGCATTAAATCATGCCAAC
AGTACTGCCGCGAATTTTTAGTGCCAGTGTGGCTGCACTGTCGTGCATTCTCCACCGCTCGCATGTACTCTCCAGGGGTATGCGT
GACAGCTTGTCCGTTACCACATTGCCAGCACACAAGCAGATATTTGAGCAGGTTAATTGGAGTGCTTTCTGTGCCTTTAACTTAT
TGCTGTAAATGCAGGCTTTTCTTCTGCATTGCTGTTGATCGGGAAGACTGATGGACATTTTGACTGTCTGCAGCATCCGATGTGA
CCCTTTCGGCGGCGTATGGTACTCATTGTTGGTCTGTTCGTTTCATGTTCGTACTGACCCTGTCAGACTAGACCAAATAGACATA
GGGGGCACAAAGATCGTTTGACCATAAGATTCTGCGGCACATGTCGCTTGCGCCACTCTAACATCGACTGTGTGCATATCATCCG
GCTGTTTAAAGGGAACCGTCTCAGTGCTGTACCGGACCAGGTTTTTCTTTGCGAGCTGGTTGTGAGGTGTAGCTGTCCTCTGGTA
GTGCTTGTGTTTGAATGGTGGACAAGAGCCTTACAAAGCGCCATTTGCTTGTATGCCAAATCAGGTCTCTTAACTGTTATGTACA
TTGTGGTAGATCATGGTTATTAATGGTACATTGTGTACATGAGTAATATAAATGCATTCACGAGCTGCTTTTATAATTGGGGGCA
CTAAAAAGTGAAGAAAATAAATGCAAAATAAACTGGCACGTCCCAAGTTTTATAAGTTGTTTTATAAAACGCGTAGGATTTAAGT
CCATTAACAGTGACCAAATGCGGACCAAGGTGGTGGCTTCCTCAAAACAACTCTGATGGCACTTTAATATGTTGGTGTTGCTTTC
TGCGCAAGGGCGAATTCTGCAGATATCCATCACACTGGCGGCCGCTCGAGCATGCATCTAGAGGGCCCAATTCGCCCTATA

>C_intestinalis_crka | cima833d10-complete
CATTATATTCGTAACTGGTTAGTTCTAGAATAGCTTTTGCAAAAGCTGTTAACAATAATGGCTTCAGCAAATTTCAATTCAGCGG
ATGAAGATTCGTGGTATTTTGGGATGGTAAGCAGGAAAGATTCGCAAAACAAGCTACTCCACCAAAAGCATGGATCTTTTTTGGT
GCGAGACAGTACTACATGTCCCGGTGACTATGTTCTCTCTGTTAGTGAAAATAGCAAAGTGAGCCACTACATTATCAACAAACAG
GAAAATAGACTTAAAATTGGAGACCAGATGTTTGACTCAATGCCAGAGTTGCTCGACTTTTACAAAGTTCATTATTTGGACACAA
CTACTTTGATTGAGCCAATTCCTAAACCTATTAAACCACAGCAAACAGTCAATCCGATTGGATCCGGAATGGTTAACATGTCATC
CATTACCTCGCCAATCAAAGAGCCAGAAGTTGCAAAAAAGAGAGAAGGACTTCCTATGATGGTGCGTGCGCTCTTCGATTTCAAG
AGTGATGATGTAGATGACCTTCCTTTCTCAAAACACGAGATTCTGGAGGTAATAGAGAAGCCAGAAGAAAATTGGTGGAATGCGA
GGAATGCACAAGGCCGTATAGGACAGATCCCTGTTCCATATGTTGAGCCCTATGTTATGAATCGTAATTCAACCCCTGGAACATA
TCAGATGCCTGGTGCACAGTTGGTTCTTCAGCCACGACCAACTTCTATGCCTTCTAGAACAGGACCTGTGTTTGCTGAAGTAATC
ACAAGAAGAGTTCCTAATGCATACGATCCAACTGCATTGGCTTTAGAGGTTGGAGACAGAATTCAAGTGACACAGATGAATAAGA
GTGGACAGTGGGAGGGCATATGCAACAACAAACAAGGCAAATTCCCATTCACCCACGTTAAACTTATTGACTTCAATGATAGTGG
TAAATCTTGATGTACCCAATTCATGTTTTTTTCTTTTTAAAGAGCAACTATTTGCAAATAAGAGGTCGTGTTTACCGGGCCAAAT
AATACTCATTGGTAATTACAATATTTTTAAGCCATTGTGTTGCAAATTGAAATCATTGCAAATGAAGGTAACATCGTAACATCCT
GGTCCTGATGTCGACTTTTACTGTAGCATATCAACACTTAATACCTGTATTAGGTATTGACTTAACCAAATCCTATTAACACTAA
TACTGCTTGCAGATATGTTTTATTTTAACCAATTCTTTGTTTTGGTTACAAAACGATTTTAGAAGAACTAGCATAAAAGATCCAT
GCATAGGCCTATAGTTAAAAATTTTACAGTATTAGTACAATTCAAAACTAATGCGTATACAAATGTGTAAACATACAGTAATAAT
TATTTAAAGTTGTACCTACAACTGGGCAATAATGGTTTTATGACACACTTACGTGTTATACTGTTAGTTATGTAATGTTTGAATC
TAATTTTTTGACCATTCACACATTAATTCAACGGCAGTTTCCCAATTTTCATAATTGCTGTCGCTGAGCATTTTGTTGCATCGAC
TGTTTGCAAATATCCAATATTACTCGATTTTATATTTTTGTTGGACGTTTTAACAAACTCTTTAACAACTGTAGATATTCTAGTT
ATATTTATCTAATATATCGATCAGTTAAAACGCTTTTTGCTATGTTAAATTTACGGAACAGCTAATATATTGACCTACTGTATCA
AGACAGCTGTCAATTGATGCAAAATGTTCAGTTTTTTTTAATTAATTTTCCTTTCTTGTTTAATATGTGTATATATACATTTCTA
CAAAACGTTTCTTGTAATTTAACAATCAACAGTTTCAACTTTAGGTTTTTATCTTTTCAAATCACCCTCAGCATGCACTCACTAT
GTGTGGTTTGTGCTTGTGTGTGTTCGTGTGGCACACTCGCCAGTTGAACTATGTGCCTTTCACGCATTGTTCAAACCCATTGAAC
GTTCTTGTACAATACCATCTCAATAGCATAAACTTTGGTTTTAAAAAAAAAAAAAAAAAAAAAAAAA

>Trichoplax_adhaerens_crka_GENSCAN-predicted cds-only | Trichoplax adhaerens strain Grell-BS-1999 TRIADscaffold_1_Cont65, whole genome shotgun sequence|ABGP01000065
ATGAAGTTCATTACAAGCTGGTTCCATGGCCCTTTATCGCGTGGTCAAGTGGACGAGCTACTTCGAGGCTCGCCTCTCGGTAGTT
TCCTAGTTCGCTCTAGTACGACACGGCCAGGAGATTATGTTTTATGTGTTCGGGAAAGCAACATGGTGAAGCATTATATCATAGA
GAAGAAAAGTGATTCTGAGTACGAAATTGGATCACAGCCTTTTCCTGATTTATTATCGGTCATAGATTTTTATAAAATTCATGTT
CTTGATACTACTGTCCTTACCGGTGCTGTTGAGGTTGAAACAGAAACGAAGGAAACAGAAGAGAACGAATCAGAAAAGAAGGAAA
CAAGTACACCTCCCAATAATAATAGCAATTCAAACACTGCGTATAAACCAAACACTGCAGAGTCCAAAATGAAGGTTATGATGCG
CGTAATTGCGTTATACGATTTTAATGCAAGGGATGAAGAAGATCTTCCGTTTACAAAGAATGAGATATTAGACGTTATCGGGATG
CAAGAAGCCAACTGGTGGACAGGTAGAAATTCAAAAGGAGCTGTAGGAGCTGTTCCTGCTCCTTATTTACGACCACTTCCGGAAG
AGGAGAGCGAAAAGTTACCCGTTATGGCAAAAGCCATTTGTGATAGGCCAAGAAATCCTTACGATCCAACCGCTCTGTCTTATCA
ATCCGGCGATATAATTAAAGTTTTAGAGAAATCTGATACTGGCTTATGGACTGGTGAATTGAAAGGACACACTGGTAAATTTCCT
TTCACTCAGGTGGTACTACTTAGTGAACTGGATGGGTCAAATGATACATGGGAAAGTTTTACAAATTAA
